# Machine learning-guided risk stratification for long QT syndrome genetic variants with hiPSC-derived cardiomyocytes

**DOI:** 10.1093/cvr/cvag105

**Published:** 2026-05-14

**Authors:** Aleksandr Khudiakov, Manuela Mura, Federica Giannetti, Vladislav Leonov, Chiara Alberio, Marem Eskandr, Paola Adele Lonati, Maria Orietta Borghi, Paul A Brink, Lia Crotti, Massimiliano Gnecchi, Peter J Schwartz, Luca Sala

**Affiliations:** Center for Cardiac Arrhythmias of Genetic Origin and Laboratory of Cardiovascular Genetics, Istituto Auxologico Italiano IRCCS, Milan, Italy; Translational Cardiology Unit, Fondazione IRCCS Policlinico San Matteo, Pavia, Italy; Center for Cardiac Arrhythmias of Genetic Origin and Laboratory of Cardiovascular Genetics, Istituto Auxologico Italiano IRCCS, Milan, Italy; Center for Cardiac Arrhythmias of Genetic Origin and Laboratory of Cardiovascular Genetics, Istituto Auxologico Italiano IRCCS, Milan, Italy; Department of Surgery, Dentistry, Pediatrics and Gynecology, Cardiovascular Science, The University of Verona, Policlinico G.B. Rossi, Verona, Italy; Center for Cardiac Arrhythmias of Genetic Origin and Laboratory of Cardiovascular Genetics, Istituto Auxologico Italiano IRCCS, Milan, Italy; Center for Cardiac Arrhythmias of Genetic Origin and Laboratory of Cardiovascular Genetics, Istituto Auxologico Italiano IRCCS, Milan, Italy; Department of Biotechnology and Biosciences, University of Milano-Bicocca, Milan, Italy; Laboratory of ImmunoRheumatologic Researches, Istituto Auxologico Italiano IRCCS, Milan, Italy; Laboratory of ImmunoRheumatologic Researches, Istituto Auxologico Italiano IRCCS, Milan, Italy; Dipartimento di Scienze Cliniche e di Comunità, Dipartimento di Eccellenza 2023-2027, University of Milan, Milan, Italy; Department of Medicine, University of Stellenbosch, Tygerberg, South Africa; Center for Cardiac Arrhythmias of Genetic Origin and Laboratory of Cardiovascular Genetics, Istituto Auxologico Italiano IRCCS, Milan, Italy; Department of Medicine and Surgery, University of Milano-Bicocca, Milan, Italy; Translational Cardiology Unit, Fondazione IRCCS Policlinico San Matteo, Pavia, Italy; Department of Molecular Medicine, Unit of Cardiology, University of Pavia, Pavia, Italy; Center for Cardiac Arrhythmias of Genetic Origin and Laboratory of Cardiovascular Genetics, Istituto Auxologico Italiano IRCCS, Milan, Italy; Center for Cardiac Arrhythmias of Genetic Origin and Laboratory of Cardiovascular Genetics, Istituto Auxologico Italiano IRCCS, Milan, Italy; Department of Biotechnology and Biosciences, University of Milano-Bicocca, Milan, Italy

**Keywords:** Precision medicine, Long QT syndrome, Machine learning, Human-induced pluripotent stem cell-derived cardiomyocytes, Pro-arrhythmic drugs

## Abstract

**Aims:**

Long QT syndrome (LQTS) is a life-threatening genetic disorder characterized by prolonged QT intervals on electrocardiograms. Congenital forms are mostly associated with variants in the *KCNQ1* and *KCNH2* genes. Among pathogenic or likely pathogenic (P/LP) variants, some are associated with a significantly higher incidence of cardiac events compared to others. While therapies have significantly reduced mortality, some patients are unresponsive or intolerant to therapy, perpetuating their arrhythmic risk, including sudden cardiac death. Current approaches for risk stratification are insufficient, highlighting the critical need for more accurate identification and management of patients carrying high-risk genetic variants. Here, we aimed to develop a refined risk stratification model for P/LP variants by applying machine learning classification to electrophysiological data measured in patient-specific human-induced pluripotent stem cell-derived cardiomyocytes (hiPSC-CMs).

**Methods and results:**

Ten patient-specific hiPSC lines, each carrying one of six pathogenic or likely pathogenic (P/LP) variants in the *KCNQ1* or *KCNH2* genes, along with two healthy control hiPSC lines, were differentiated into hiPSC-CMs. Electrophysiological responses from multielectrode array recordings at baseline and after application of selective ion channel blockers or pro-arrhythmic compounds were used to train a machine learning model to classify variant-specific risk levels based on *in vitro* electrophysiological readouts. An independent validation cohort of two additional *KCNH2* hiPSC lines was used to test the model’s performance in predicting single-variant risk. Our findings revealed a correlation between variant risk level, hiPSC-CM electrophysiological profiles, and drug responses. The machine learning classifier, trained on multielectrode array recordings, achieved 89% accuracy in the classification of P/LP genetic variants according to the associated risk levels.

**Conclusion:**

This study demonstrates that integrating hiPSC-CM electrophysiological profiling with machine learning provides a robust method for granular variant-specific risk stratification of LQTS patients.

## Graphical Abstract

**Graphical Abstract:**
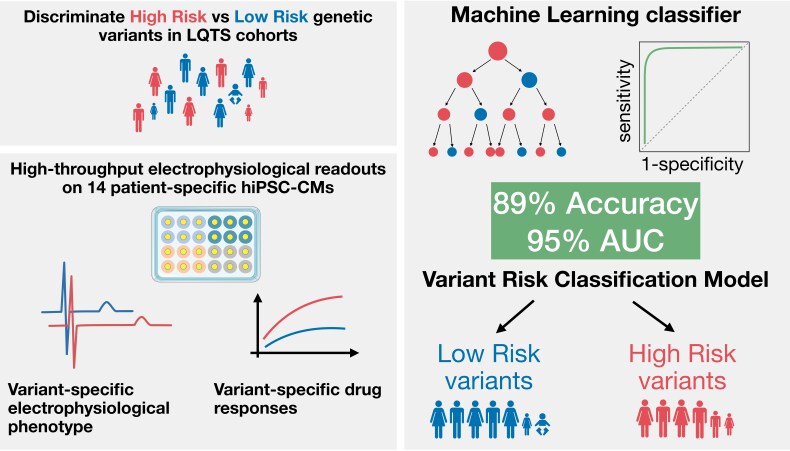
A schematic overview of the pipeline described in this study, integrating multielectrode array readouts from patient-specific hiPSC-derived cardiomyocytes with machine learning-based classification of genetic variant risk.

**Time of primary review: 58 days**

**See the editorial comment for this article ‘Human-induced pluripotent stem cell cardiomyocytes for risk stratification of patients with LQT syndrome—ready for practice?’, by T. Eschenhagen, https://doi.org/10.1093/cvr/cvag126.**

## 1. Introduction

The long QT syndrome (LQTS) is a life-threatening disease of genetic origin characterized by a prolonged QT interval on the electrocardiogram, by propensity to lethal arrhythmias, especially under stress, and by an increased sensitivity to drugs affecting cardiac repolarization through block of the I_Kr_ potassium current.^1–4^ Appropriate therapies based on beta-blockers,^5^ left cardiac sympathetic denervation,^6,7^ and sodium channel blockers^8,9^ have effectively reduced the risk for patients to develop potentially lethal ventricular arrhythmias or sudden cardiac death (SCD), limiting the use of an implantable cardioverter defibrillator (ICD) only to selected patients^5,10^; one of the main challenges for clinicians dealing with LQTS is the identification, clinical management, and protection of patients who are more at risk of developing SCD.

Advancements in next-generation sequencing have led to the discovery of numerous variants in genes encoding cardiac ion channels, subunits, or associated proteins implicated in LQTS.^11,12^ While these insights have guided gene-specific patient management,^8^ current genetic interpretation guidelines^13^ often do not help identify patients at higher risk, with a large number of variants classified as variants of uncertain significance (VUS). Even variants classified as pathogenic or likely pathogenic (P/LP) according to the American College of Medical Genetics and Genomics (ACMG) guidelines may exhibit divergent risks of life-threatening cardiac events, including SCD,^14,15^ requiring profoundly different clinical management.

Accurate clinical risk assessment for a variant requires data from many affected individuals, and this is not feasible for most of the variants, limiting precision medicine and hampering the development of variant-specific therapies.^16,17^ The classification of variant pathogenicity through *in vitro* or *in vivo* studies offers a promising solution to address this limitation.^18,19^ Functional studies using patient-specific cardiomyocytes derived from human-induced pluripotent stem cells (hiPSC-CMs) further refined this, providing high-quality functional data on the effect of the variants on action potentials, calcium transients, or contractility. HiPSC-CM-based models have been particularly useful for disease modelling of congenital or acquired cardiac disorders such as LQTS,^20–24^ Jervell and Lange-Nielsen syndrome (JLNS),^25^ Timothy syndrome,^26^ cardiomyopathies,^27–29^ congenital heart defects,^30^ drug testing,^31–33^ and drug repurposing.^34^ In the present study, using hiPSC-CMs from 10 patients, we focused on six well-characterized P/LP variants from the two most prevalent LQTS subtypes, LQT1 and LQT2, which together account for 90% of cases. These variants were identified in individuals with clinically heterogeneous presentations, from normal to prolonged QT intervals and displaying low or high incidence of life-threatening cardiac events per variant (defined here as low- and high-risk P/LP variants).

Using patient-derived hiPSC-CMs and high-throughput multielectrode arrays (MEA), we investigated (i) whether hiPSC-CMs carrying P/LP variants with differing clinical severities exhibit distinct drug responses *in vitro*, (ii) whether *in vitro* phenotypes match clinical records and could be used for a more accurate variant-associated risk stratification, and (iii) whether accurate P/LP variant risk stratification can be automated through machine learning on *in vitro* readouts.

## 2. Methods

### 2.1 Ethical statement

This study was conducted in accordance with the Declaration of Helsinki, and the ethics committee of Istituto Auxologico Italiano IRCCS gave ethical approval for this work (Approval number: 2020_10_20_07). Appropriate informed consents were obtained from all donors.

### 2.2 Patient-specific hiPSC lines included in the study

A total of 14 hiPSC lines were utilized in this study, including 12 patient-specific and 2 wild-type lines. Patient-specific hiPSC lines were derived from LQT1, JLNS, and LQT2 patients carrying a total of four *KCNQ1* variants and four *KCNH2* variants (See *Table 1* for detailed information). The lines carrying the *KCNH2* p.A561V, *KCNH2* p.T983I, and *KCNH2* p.R823W variants were provided by Joseph C. Wu, MD, PhD, at the Stanford Cardiovascular Institute. Detailed characterization of each hiPSC line, including genetic and functional properties, is provided in Supplementary material online, *Table S1* and Supplementary material online, *Figures S1*–*S5*. Two male bona fide wild-type hiPSC lines were used: the commercially available WTC-11 line obtained from the Coriell Institute for Medical Research (catalog No. GM25256) (WT1)^35^ and a second line derived from a healthy donor (WT2). Unless otherwise specified, electrophysiological data from the two wild-type lines were pooled and collectively labelled as WT for analysis purposes.

**Table 1:** Clinical characteristics of the patient cohort included in the study

Patient	Disease	Genetic variant	dbSNP record	ClinVar classification	Known modifier genes	Sex	QTc (ms)	Schwartz score	Age	Symptomatic	Clinical symptoms	Treatment
1	LQT1	*KCNQ1* p.R190W	rs199473662	P/LP		F	458	2	44	No		Propranolol
2	LQT1	*KCNQ1* p.R594Q	rs199472815	P/LP		M	448	1	47	No		
3	LQT1	*KCNQ1* p.A341V	rs12720459	P	*NOS1AP* rs16847548 homozygous minor allele and *NOS1AP* rs4657139 homozygous minor allele	F	593	6	70	Yes	Syncope with stress at 16 y.o.	
4	LQT1	*KCNQ1* p.A341V	rs12720459	P	*NOS1AP* rs16847548 heterozygous minor allele and *NOS1AP* rs4657139 heterozygous minor allele	F	501	6	50	Yes	Syncope with stress at 6 y.o.	
5	LQT1	*KCNQ1* p.A341V	rs12720459	P	*NOS1AP* rs16847548 homozygous major allele and *NOS1AP* rs4657139 homozygous major allele	M	406	1	74	No		
6	LQT1	*KCNQ1* p.A341V	rs12720459	P	*NOS1AP* rs16847548 homozygous major allele and *NOS1AP* rs4657139 homozygous major allele	F	488	4.5	59	No		
7	JLNS	*KCNQ1* p.R190W, *KCNQ1* p.R594Q	rs199473662, rs199472815	P/LP(both variants)		F	578	7.5	17	Yes	Multiple syncopal episodes since age 2, deafness.	Propranolol
8	LQT2	*KCNH2* p.R366X	rs794728364	P		F	480	5.5	54	No	Notched t-waves.	Nadolol
9	LQT2	*KCNH2* p.R366X	rs794728364	P		F	622	7.5	38	Yes	Syncope after a wake-up alarm sound at 28 y.o. No cardiac arrest history.	Nadolol, concomitant mexiletine; LCSD performed at age 30.
10	LQT2	*KCNH2* p.A561V	rs121912504	P/LP		M	480 (with nadolol)	-	25	Yes	Two cardiac arrests, no cardiac events after beta-blocker and ICD treatment.	Nadolol, ICD implanted.
**Variants used for validation**												
11	LQT2	*KCNH2* p.T983I	rs149955375	VUS		M	482 (380 with nadolol)	-	39	Yes	Palpitations, syncope during physical activity.	Nadolol
12	LQT2	*KCNH2* p.R823W	rs199473538	P/LP		F	463	-	25	Yes	Two syncopal episodes.	ICD implanted.

### 2.3 Cardiac events frequency assessment

Clinical data on documented cardiac events (syncope, sustained ventricular tachycardia, appropriate ICD shock, sudden cardiac arrest, and sudden cardiac death), QTc values, and Schwartz scores (when reported) were extracted from our internal database of LQTS patients, the Human Gene Mutation Database (HGMD),^36^ and the Variant Browser database.^37,38^ The frequency of cardiac events was calculated as the proportion of patients with a history of such events relative to the total number of patients carrying the specific variant.

### 2.4 HiPSC culture and differentiation to hiPSC-CMs

HiPSCs were cultured on multi-well plates coated with recombinant human vitronectin (Gibco) in Essential 8 Flex medium kit (Gibco). HiPSCs were plated on Matrigel-coated (Corning) plates for cardiac differentiation. HiPSCs were differentiated to hiPSC-CMs following a small molecule-based protocol,^39^ purified through glucose starvation,^40^ starting from day 7, and cryopreserved in Bambanker (Nippon Genetics) at days 9-16. For the subsequent experiments, hiPSC-CMs were thawed, replated at low density, and expanded as previously reported.^41^ The purity of hiPSC-CM differentiations was assessed using flow cytometry analysis and quantified as the percentage of live cells expressing cardiac troponin T (on average >90% cTnT+ cells, Supplementary material online, *Figure S6*). Mycoplasma testing was routinely performed to assess the absence of contamination using the N-GARDE Mycoplasma PCR Reagent Set (Euroclone). A list of main reagents used in the study is reported in the Supplementary material online, *Table S2*. Data were collected from at least three independent differentiations of each hiPSC line for each experiment.

### 2.5 Patch clamp

For patch clamp experiments, metabolic maturation of hiPSC-CMs was performed as previously described.^42^ Action potentials and I_Ks_ currents were recorded at 37°C from isolated hiPSC-CMs plated on glass coverslips as described in detail in the Supplementary material online, Methods section. Action potentials were recorded in perforated patch mode under 1 Hz pacing.

### 2.6 Multielectrode arrays

HiPSC-CMs were plated on 24-well multi-well MEAs (MultiChannel Systems) coated with bovine fibronectin (Merck) as previously described.^43,44^ The recordings were performed on spontaneously beating hiPSC-CMs at 37°C. The detailed description of drug treatments and analysis of the recordings is present in the Supplementary material online, Methods section.

### 2.7 Pro-arrhythmic compound selection

The unguided selection of pro-arrhythmic compounds was performed using the CredibleMeds® QT prolonging drugs database^45^ intersected with the OpenFDA database (https://open.fda.gov/, accessed on 17/04/2024). OpenFDA data were used to prioritize compounds based on their occurrence in the FDA Adverse Event Reporting System. The following query terms were utilized to extract the top 100 compounds from the OpenFDA database: ‘*Cardiac failure*’, ‘*Torsade de pointes*’, ‘*Electrocardiogram QT prolonged*’, ‘*Cardiac arrest*’, ‘*Ventricular extrasystoles*’, and ‘*Ventricular arrhythmia*’. The common compounds between databases were 46 (Supplementary material online, *Table S3*). We excluded compounds that were unlikely to provide pro-arrhythmic effects in a pure culture of hiPSC-CMs (such as diuretics or drugs associated with chronic cardiotoxicity) as beyond the scope of this study. Seven drugs were then selected in the highest-risk category, i.e. those contraindicated in congenital LQTS and are known to pose a significant risk for developing drug-induced Torsades de Pointes (TdP): albuterol (salbutamol), chlorpromazine, ciprofloxacin, clarithromycin, dofetilide, haloperidol, and moxifloxacin. Detailed information on the compounds is provided in the Supplementary material online, *Table S4*.

### 2.8 Data analysis and statistics

RStudio (version 2026.01.0 + 392) and R (version 4.5.2) were used for data analysis. Multiple group comparisons were conducted using the Kruskal-Wallis test, followed by Dunn’s test with Benjamini-Hochberg correction, or two-way ANOVA, followed by pairwise *t*-tests with Bonferroni correction where appropriate. Pairwise comparisons were performed using the Wilcoxon rank-sum test. Categorical variable comparisons were assessed using Fisher’s exact test. A statistical significance level of *P* ≤ 0.05 was used for all tests. Figures denote significance as *P ≤ 0.05, **P ≤ 0.01, and ***P ≤ 0.001, while exact *P*-values are reported in text and tables. Numerical data are reported as mean ± standard error of the mean (SEM), and plots display mean ± SEM, combined with scattered individual data points when relevant. Categorical data are presented as bar plots.

### 2.9 Machine learning model for variant risk prediction

The primary dataset, comprising 11109 rows from performed MEA measurements, was used as input for a random forest classification model implemented using the comprehensive *tidymodels* framework.^46^ Each row in the dataset represented a single measurement (baseline, vehicle, or drug at a given concentration) from a single well of a multi-well MEA plate. Consequently, each genetic variant was represented by multiple rows, ranging from 989 to 3487. The model was trained on the following features: *drug*, *concentration*, *raw and normalized mean field potential duration (FPD)*, *raw and normalized mean RR interval*, *raw and normalized mean peak-to-peak amplitude (PtPA)*, *mean slope*, *RR interval coefficient of variation*, *quality score*, and either *variant risk level* or *disease-causing gene*. These features enabled the model to perform dichotomous classification of variant risk level or multiclass classification of disease-causing gene. The dataset was stochastically divided into training and validation subsets with a ratio of 70:30, ensuring that drug readouts were evenly distributed between the two datasets. Variant risk level or disease-causing gene was predicted independently for each row. A 10-fold cross-validation was conducted to evaluate overall model performance.

To further evaluate the model’s performance in classifying individual variant risk, the model trained on the primary dataset was applied to predict risk of the variants in the validation cohort. In addition, using pooled MEA measurements from all hiPSC-CMs, a leave-one-out cross-validation analysis was performed. For each iteration, data corresponding to one variant were excluded, the model was trained on the remaining data, and predictions were made on the held-out subset. The accuracy of variant prediction was calculated as the percentage of correctly predicted readouts (rows) on the total number of readouts of the dataset.

## 3. Results

### 3.1 Study design and patient cohort characteristics

The study was designed around a primary experimental cohort of ten patients and an independent validation cohort of two patients, requested during the revision phase. Clinical characteristics of all patients are summarized in *Table 1*. The primary cohort included ten hiPSC lines carrying six LQTS-associated variants, along with two wild-type hiPSC lines. Two additional LQT2 hiPSC lines were used as a further independent validation dataset to assess the generalizability of the findings.

The primary study cohort comprised a LQT1 family trio (Supplementary material online, *Figure S7*), which includes an asymptomatic mother carrying the *KCNQ1* p.R190W variant, an asymptomatic father carrying the *KCNQ1* p.R594Q variant, and their daughter affected by JLNS^47^, carrying both variants in compound heterozygosity.^48^ Four patients from a large South African family carrying the *KCNQ1* p.A341V variant associated with a very severe LQT1 phenotype^49–51^; two of these patients were asymptomatic, while the other two were severely symptomatic, carrying polymorphisms in the modifier gene *NOS1AP* (one patient in a heterozygous state and one patient in a homozygous state) associated with a prolonged QT interval and with an increased risk of life-threatening events.^52^ Among the LQT2 cases, two patients carried the *KCNH2* p.R366X variant: one symptomatic and one asymptomatic. Both were treated with beta-blockers. The symptomatic patient also underwent left cardiac sympathetic denervation due to difficulties in optimizing beta-blocker therapy because of asthenia. No recurrence of symptoms was observed on therapy. A third symptomatic LQT2 patient, carrying the *de novo KCNH2* p.A561V variant, experienced cardiac arrests and was treated with beta-blockers and an ICD implantation.

### 3.2 Genetic variant risk level assignment

**Figure 1:**
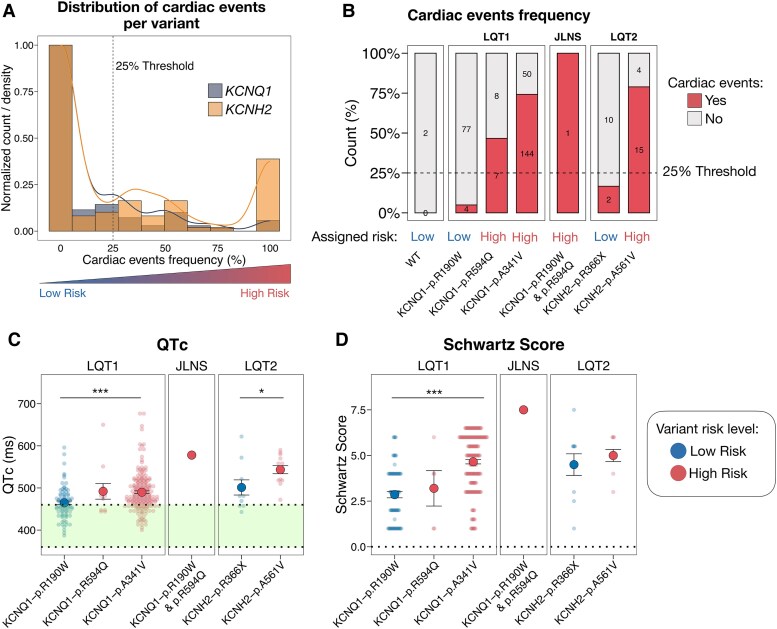
Clinical parameters and variant risk level assignment. (*A*) Frequency of cardiac events across pathogenic/likely pathogenic genetic variants in *KCNQ1* (blue) and *KCNH2* (orange) genes. (*B*) Percentage of patients with cardiac events for each variant from both our clinical cohort and literature data (*N* = 1-194), with assigned variant risk level indicated. (*C* and *D*) Baseline QTc and Schwartz score values for each variant (*N* = 1-183). Kruskal-Wallis test followed by Dunn’s multiple comparisons test with Benjamini-Hochberg correction. *P ≤ 0.05, **P ≤ 0.01, ***P ≤ 0.001.

To define the threshold for categorizing variants associated with different risks of cardiac events (low risk vs. high risk), we analyzed the frequency distribution of cardiac events across reported P/LP genetic variants in *KCNQ1* and *KCNH2* genes (1099 patients carrying 162 variants in *KCNQ1* and 623 patients carrying 175 variants in *KCNH2*) using our clinical repository of LQTS patients (*Figure 1A*). For both genes, two distinct peaks in the distribution were identified: one at 0% frequency, indicating that the majority of the variants had no reported cardiac events, and another at ≥25% frequency, indicating variants where at least 25% of carriers experienced cardiac events. Based on this analysis, a threshold of 25% was selected to differentiate these populations. Using this threshold, the variants in our study were categorized as follows: high risk (*KCNQ1* p.R594Q and *KCNQ1* p.R190W & p.R594Q, *KCNQ1* p.A341V, and *KCNH2* p.A561V) and low risk (WT, *KCNQ1* p.R190W, and *KCNH2* p.R366X) (*Figure 1B*).

Consistent with the incidence of cardiac events, carriers of high-risk variants *KCNQ1* p.A341V and *KCNH2* p.A561V had significantly longer QTc values compared to carriers of the low-risk variants *KCNQ1* p.R190W and *KCNQ1* p.R366X, respectively (*Figure 1C*, Supplementary material online, *Table S5*). In addition, the Schwartz scores of *KCNQ1* p.A341V carriers were higher than those of *KCNQ1* p.R190W (*Figure 1D*, Supplementary material online, *Table S5*). Notably, the *KCNQ1* p.R190W & p.R594Q variant, observed in a single carrier, presented with deafness and exhibited markedly prolonged QTc (578 ms) and elevated Schwartz score (7.5), consistent with the severe JLNS phenotype.

### 3.3 *In vitro* characterization of genetic variants selected for the study

**Figure 2:**
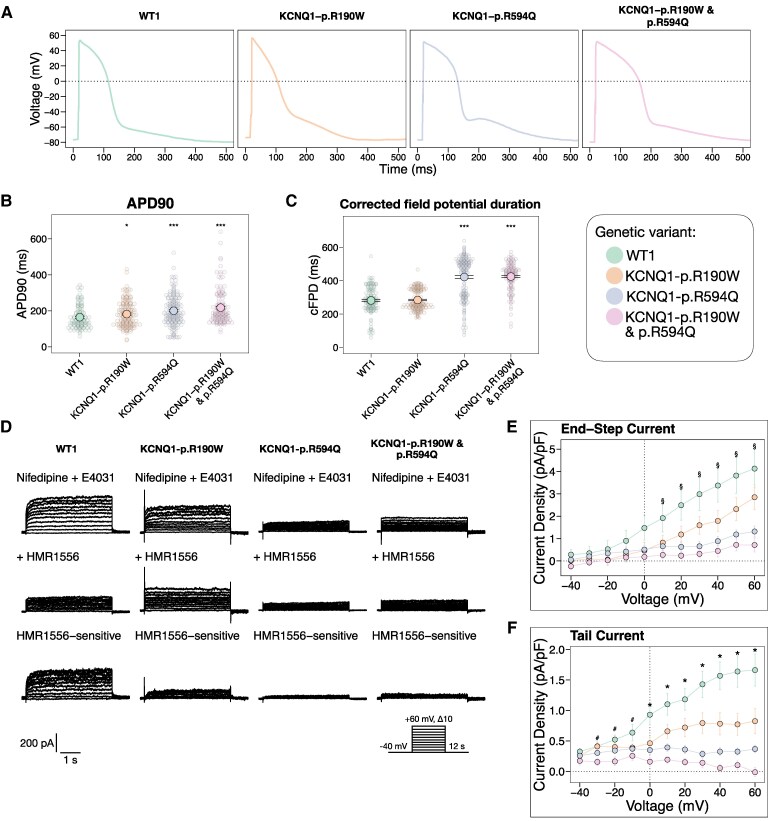
Characterization of hiPSC-CMs carrying genetic variants *KCNQ1* p.R190W and *KCNQ1* p.R594Q. (*A*) Representative action potential traces for each hiPSC-CM variant, elicited at 1 Hz stimulation. (*B*) Action potential duration at 90% repolarization (APD90). (*C*) Corrected field potential duration (cFPD) measurements in hiPSC-CMs for each variant (*N* = 157-180). Wilcoxon rank-sum test vs. WT group *P ≤ 0.05, **P ≤ 0.01, ***P ≤ 0.001. (*D*) Representative I_Ks_ current traces (lower panel), obtained by subtracting HMR1556-insensitive current (middle panel) from the total inward potassium current recording (upper panel). (*E* and *F*) End-step and tail I_Ks_ current-voltage (*I-V*) relationships. Two-way ANOVA followed by pairwise *t*-tests with Bonferroni correction * denotes *P* ≤ 0.05 for all groups vs. WT, # for WT vs. *KCNQ1* p.R190W & p.R594Q, § for WT vs. KCNQ1 p.R594Q and vs. KCNQ1 p.R190W & p.R594Q.

A detailed functional characterization had previously been reported in literature on four out of six variants using heterologous systems or hiPSC-CM models (summarized in Supplementary material online, *Table S6*). Specifically, *KCNQ1* p.R594Q, *KCNQ1* p.A341V, *KCNH2* p.R366X, and *KCNH2* p.A561V were shown to be loss-of-function variants, with *KCNQ1* p.R594Q and *KCNH2* p.A561V also being trafficking-deficient.^34,49,53–60^ In contrast, *KCNQ1* p.R190W and the compound variant *KCNQ1* p.R190W & p.R594Q had not been previously characterized. To address this gap, we performed electrophysiological characterizations using hiPSC-CMs derived from the family trio (carrying *KCNQ1* p.R190W, *KCNQ1* p.R594Q, and *KCNQ1* p.R190W & p.R594Q) along with control WT1 hiPSC-CMs (*Figure 2*).

We observed the repolarization prolongation at the single-cell level (action potential duration at 90% repolarization, APD90) and a reduced ability to follow high-frequency pacing in isolated hiPSC-CMs carrying *KCNQ1* p.R190W, *KCNQ1* p.R594Q, and *KCNQ1* p.R190W & p.R594Q compared to WT1 (*Figure 2A* and *B*; Supplementary material online, *Figure S8A* and *B*). HiPSC-CM monolayers carrying *KCNQ1* p.R594Q and *KCNQ1* p.R190W & p.R594Q demonstrated also corrected FPD (cFPD) prolongation in comparison to WT1 (422.2 ± 126.7 ms and 424.9 ± 78.7 ms vs. 281.8 ± 77.4 ms, respectively, *P* ≤ 0.001) (*Figure 2C*). Measurement of I_Ks_ with patch clamp demonstrated decreased end-step and tail current densities in hiPSC-CMs carrying *KCNQ1* p.R190W, compared to WT1, and almost absent I_Ks_ in hiPSC-CMs carrying *KCNQ1* p.R594Q and *KCNQ1* p.R190W & p.R594Q, in line with their APD prolongation (*Figure 2D-F*).

### 3.4 Baseline electrophysiology of hiPSC-CM cohort

**Figure 3:**
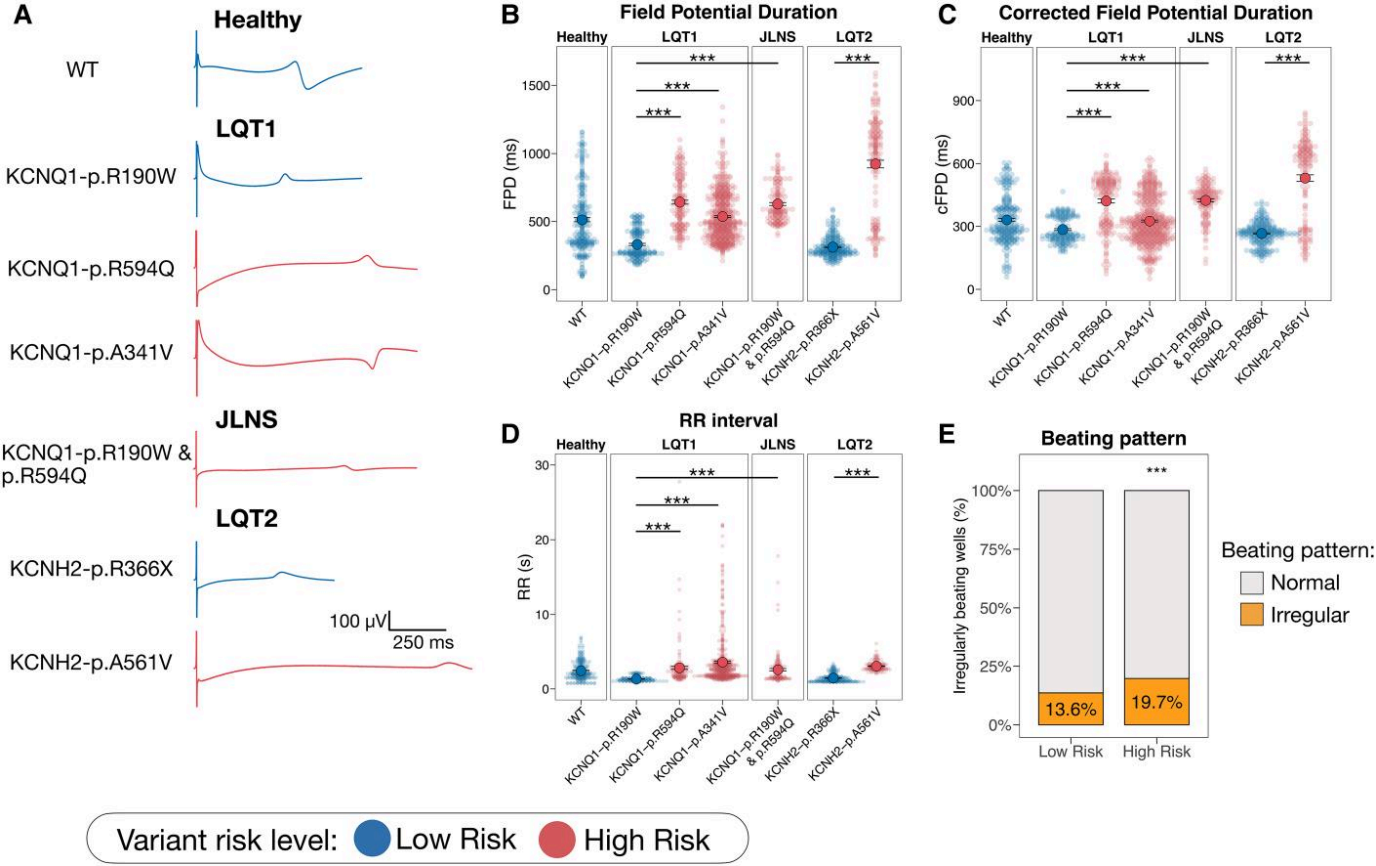
Baseline electrophysiology of hiPSC-CMs carrying high-risk and low-risk genetic variants. (*A*) Representative field potentials obtained for each variant with MEAs on spontaneously beating hiPSC-CM monolayers. (*B-D*) Baseline field potential duration (FPD), corrected FPD (cFPD), and beat-to-beat interval (RR) measurements of hiPSC-CMs for each variant. *N* = 157-547. (*E*) Incidence of regular (grey) or irregular (yellow) beating patterns for hiPSC-CMs carrying low-risk and high-risk variants (*N* = 698-1038). Kruskal-Wallis test followed by Dunn’s multiple comparisons test with Benjamin-Hochberg correction. Fisher’s exact test for categorical variables comparison. *P ≤ 0.05, **P ≤ 0.01, ***P ≤ 0.001.

For the subsequent experiments, we analysed pools of hiPSC-CMs, stratifying them by variant or variant risk level. A second wild-type line (WT2) was included to account for the known variability exhibited by bona fide wild-type hiPSCs.^61^ Baseline hiPSC-CM electrophysiology assessed with MEA measurements accurately reflected the risk level assigned to each variant. Specifically, hiPSC-CMs from the high-risk group exhibited increased baseline FPD, cFPD, and RR interval duration (*Figure 3A-D*; *Table 2*) compared to gene-matched hiPSC-CMs from the low-risk group. Field potential quality scoring (Supplementary material online, *Figure S9*) revealed an increased propensity for irregular beating patterns at baseline in the high-risk group (19.7% of wells in the high-risk group vs. 13.6% in the low-risk group, *P* ≤ 0.001) (*Figure 3E*).

**Table 2:** Baseline electrophysiological parameters obtained from MEA recording for each variant

Variant	N	FPD (ms)	cFPD (ms)	RR (s)
WT	263	512.6 ± 244.9	331 ± 112	2.43 ± 1.22
*KCNQ1*-p.R190W	180	331.3 ± 104.9	284.6 ± 60.6, *P* ≤ 0.001	1.33 ± 0.34, *P* ≤ 0.001
*KCNQ1*-p.R594Q	173	643.7 ± 190.2, *P* ≤ 0.001 vs. *KCNQ1*-p.R190W	422.2 ± 126.7, *P* ≤ 0.001 vs. *KCNQ1*-p.R190W	2.82 ± 2.6, *P* ≤ 0.001 vs. *KCNQ1*-p.R190W
*KCNQ1*-p.A341V	547	536.9 ± 173.2, *P* ≤ 0.001 vs. *KCNQ1*-p.R190W	325.1 ± 109.2, *P* ≤ 0.001 vs. *KCNQ1*-p.R190W	3.57 ± 3.71, *P* ≤ 0.001 vs. *KCNQ1*-p.R190W
*KCNQ1*-p.R190W & p.R594Q	157	629.8 ± 133, *P* ≤ 0.001 vs. *KCNQ1*-p.R190W	424.9 ± 78.7, *P* ≤ 0.001 vs. *KCNQ1*-p.R190W	2.56 ± 2.05, *P* ≤ 0.001 vs. *KCNQ1*-p.R190W
*KCNH2*-p.R366X	255	311.4 ± 74.3	265.9 ± 53.4	1.45 ± 0.56
*KCNH2*-p.A561V	161	923.1 ± 355.4, *P* ≤ 0.001 vs. *KCNH2*-p.R366X	531.1 ± 197.3, *P* ≤ 0.001 vs. *KCNH2*-p.R366X	3.04 ± 0.61, *P* ≤ 0.001 vs. *KCNH2*-p.R366X
**Variants used for validation**				
*KCNH2*-p.T983I	143	532.5 ± 69.2, *P* ≤ 0.001 vs. *KCNH2*-p.R366X	361 ± 51.7, *P* ≤ 0.001 vs. *KCNH2*-p.R366X	2.2 ± 0.27, *P* ≤ 0.001 vs. *KCNH2*-p.R366X
*KCNH2*-p.R823W	174	596.3 ± 154, *P* ≤ 0.001 vs. *KCNH2*-p.R366X	322.3 ± 172.2, *P* ≤ 0.001 vs. *KCNH2*-p.R366X	5.76 ± 4.75, *P* ≤ 0.001 vs. *KCNH2*-p.R366X

Kruskal-Wallis test followed by Dunn’s multiple comparisons test with Benjamini-Hochberg correction.

### 3.5 HiPSC-CMs from high-risk and low-risk groups demonstrate distinct drug responses to ion channel blockers

**Figure 4:**
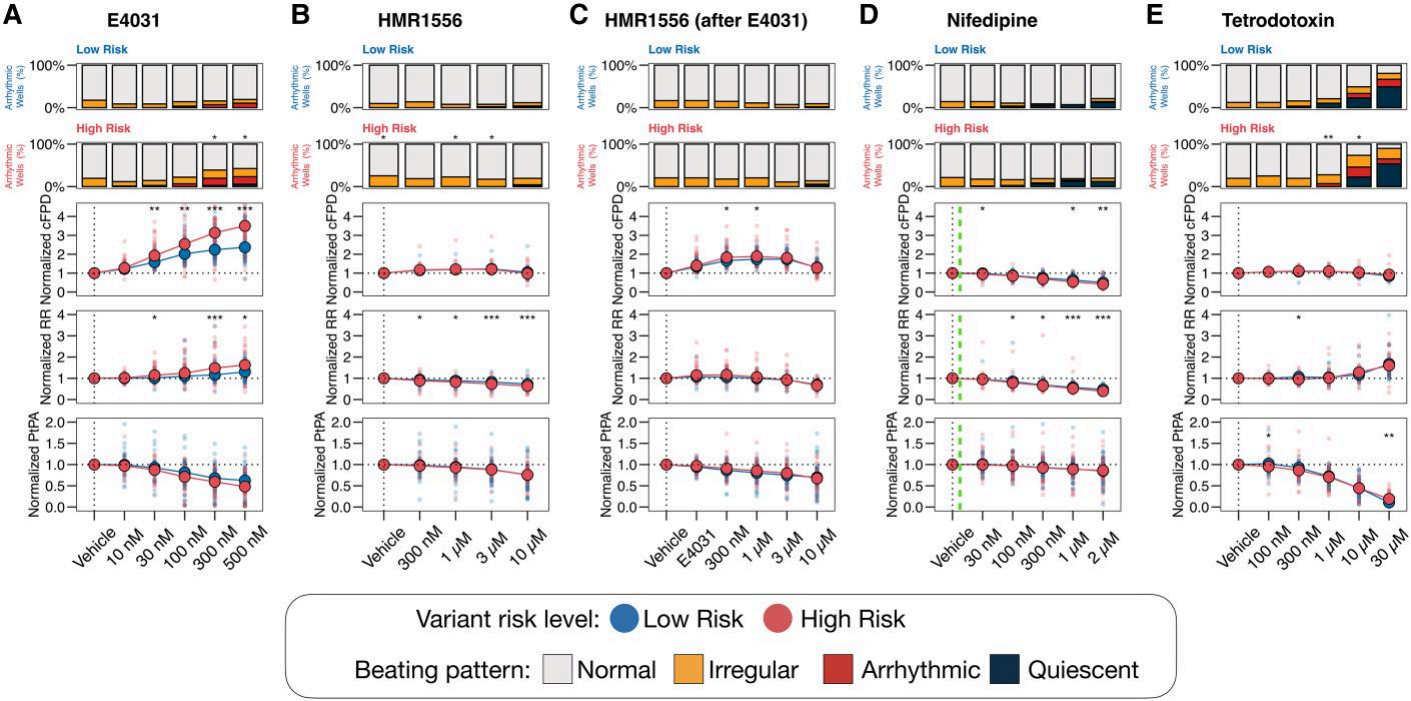
HiPSC-CMs from low-risk and high-risk groups exhibit differential responses to ion channel blockers. Readouts from spontaneously beating hiPSC-CMs corresponding to the low-risk group (*KCNQ1* p.R190W, *KCNH2* p.R366X, WT) and the high-risk group (*KCNQ1* p.R594Q, *KCNQ1* p.R190W & p.R594Q, *KCNQ1* p.A341V, *KCNH2* p.A561V) were pooled to create low-risk and high-risk cohorts. (*A-E*) Concentration-dependent response curves to ion channel blockers E4031, HMR1556, HMR1556 following E4031 pre-treatment, nifedipine, and tetrodotoxin. Responses are shown for normalized corrected field potential duration (normalized cFPD), normalized RR interval (normalized RR), and normalized peak-to-peak amplitude (normalized PtPA). Bar plots illustrate the distribution of beating pattern abnormalities. The green vertical dashed line indicates reported Cmax value for nifedipine. Asterisks indicate statistical significance of comparisons between the high-risk and low-risk groups. *N* = 51-80 per group per drug concentration. Wilcoxon rank-sum test for two groups comparison. Fisher’s exact test for categorical variables comparison. *P ≤ 0.05, **P ≤ 0.01, ***P ≤ 0.001.

**Figure 5:**
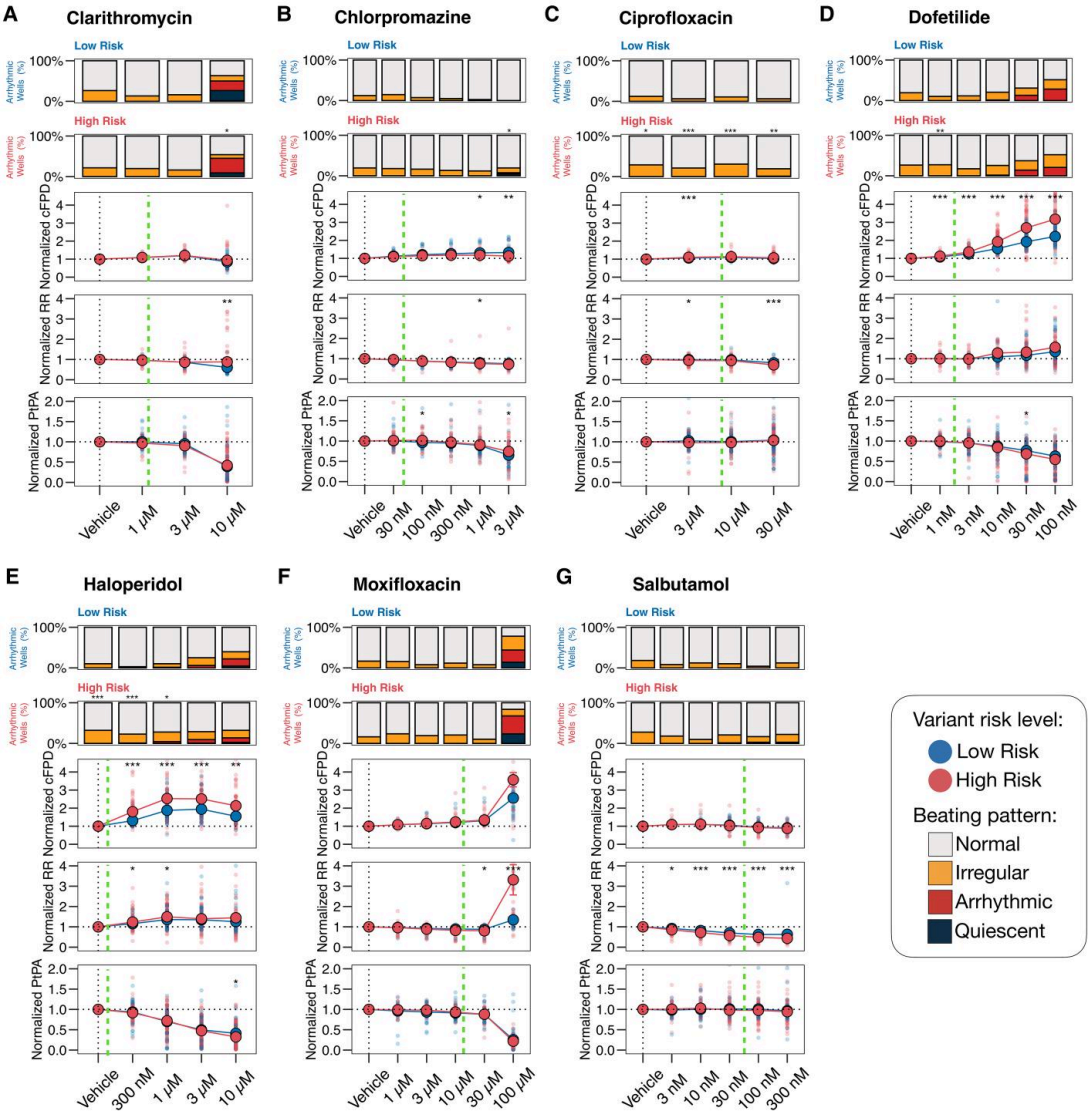
HiPSC-CMs from low-risk and high-risk groups exhibit differential responses to pro-arrhythmic drugs. Readouts from spontaneously beating hiPSC-CMs corresponding to the low-risk group (*KCNQ1* p.R190W, *KCNH2* p.R366X, WT) and the high-risk group (*KCNQ1* p.R594Q, *KCNQ1* p.R190W & p.R594Q, *KCNQ1* p.A341V, *KCNH2* p.A561V) were pooled to create low-risk and high-risk cohorts. (*A-G*) Concentration-dependent response curves for pro-arrhythmic drugs clarithromycin, chlorpromazine, ciprofloxacin, dofetilide, haloperidol, moxifloxacin, and salbutamol. Responses are presented for normalized corrected field potential duration (normalized cFPD), normalized RR interval (normalized RR), and normalized peak-to-peak amplitude (normalized PtPA). Bar plots illustrate the distribution of beating pattern abnormalities. The green vertical dashed lines indicate reported Cmax values for each drug. Asterisks indicate statistical significance of comparisons between the high-risk and low-risk groups. *N* = 41-121 per group per drug concentration. Wilcoxon rank-sum test for two groups comparison. Fisher’s exact test for categorical variables comparison. *P ≤ 0.05, **P ≤ 0.01, ***P ≤ 0.001.

To further discriminate hiPSC-CMs carrying variants with different risk levels, we obtained concentration-response curves for 11 selected drugs (*Figures 4* and *5*; Supplementary material online, *Figures S10*–*S21*; Supplementary material online, *Table S7*). Selective ion channel blockers E4031 (I_Kr_, Kvv11.1), HMR-1556 (I_Ks_, K_v_7.1), nifedipine (I_CaL_, Ca_v_1.2), and tetrodotoxin (I_Na_, Na_v_1.5) were used to assess ion channel-specific drug responses as previously proposed by the multiple ion channel effects (MICE) approach for drug testing assays.^62^ Since I_Ks_ blockers were previously reported to be more effective with reduced cardiac repolarization reserve caused by *KCNH2* genetic variants or I_Kr_ pharmacological block,^63^ we also tested the effect of HMR1556 on hiPSC-CMs pre-treated with 20 nM I_Kr_ blocker E4031.

Among pure ion channel blockers, we observed a differential response to the I_Kr_ blocker E4031 and I_Ks_ blocker HMR1556 after E4031 pre-treatment between hiPSC-CMs carrying high-risk and low-risk variants. Specifically, hiPSC-CMs with high-risk variants showed a greater concentration-dependent prolongation of cFPD following treatment with E4031 (up to 350.0 ± 183.2% vs. Vehicle for high risk and up to 236.9 ± 84.4% vs. Vehicle for low risk, *P* ≤ 0.001). Furthermore, E4031 increased the RR interval duration in hiPSC-CMs carrying high-risk variants more than in those carrying low-risk variants (*Figure 4A*). The high-risk group also demonstrated a higher incidence of beating abnormalities at high concentrations of E4031 (*Figure 4A*). HMR1556 treatment alone resulted in approximately 20% cFPD prolongation relative to baseline in both the high-risk and low-risk groups (*Figure 4B*). In contrast, the combination of E4031 pre-treatment, followed by HMR1556 concentration-response, led to a greater cFPD prolongation relative to baseline, which appeared discriminative between high-risk and low-risk groups (up to 188.4 ± 40.6% vs. Vehicle for high risk and up to 173.8 ± 30.6% vs. Vehicle for low risk, *P* = 0.05) (*Figure 4C*). Treatment with the I_CaL_ blocker nifedipine led to a cFPD and RR interval decrease (*Figure 4D*). High concentrations of the I_Na_ blocker tetrodotoxin (10 µM and 30 µM) led to a decrease in peak-to-peak amplitude and prolongation of the RR interval, along with an increased incidence of beating abnormalities (*Figure 4E*). The high-risk group exhibited significantly more beating abnormalities at 1 µM and 10 µM of tetrodotoxin compared to the low-risk group.

### 3.6 HiPSC-CMs carrying high-risk and low-risk P/LP variants demonstrate distinct drug responses to pro-arrhythmic drugs

In addition to pure ion channel blockers seven pro-arrhythmic compounds were selected using the OpenFDA and CredibleMeds databases, as described in the Methods: β2-adrenergic receptor agonist albuterol (salbutamol); the antibiotics clarithromycin, ciprofloxacin, and moxifloxacin; the antipsychotic drugs chlorpromazine and haloperidol; and the antiarrhythmic drug dofetilide. Among these compounds, dofetilide, haloperidol, and the β2-adrenergic receptor agonist salbutamol demonstrated differential concentration-response curves between high-risk and low-risk groups (*Figure 5*). Specifically, responses to the antibiotics clarithromycin and ciprofloxacin were similar between the two groups (*Figure 5A* and *C*). The highest concentration of clarithromycin tested (10 µM) led to arrhythmias and cessation of beating (*Figure 5A*). Another antibiotic, moxifloxacin, demonstrated a similar effect, with notable toxicity at a concentration of 100 µM, expressed by cFPD and RR increases, a drop in peak-to-peak amplitude, arrhythmias, and cessation of beating similar for both groups (*Figure 5F*). Treatment of hiPSC-CMs with the antipsychotic drug chlorpromazine resulted in moderate cFPD prolongation that was similar between the two groups (*Figure 5B*). hiPSC-CMs carrying high-risk variants showed a concentration-dependent increase in cFPD following treatment with the dofetilide (up to 317.5 ± 128.5% vs. Vehicle for high risk and up to 221.6 ± 65.8% vs. Vehicle for low risk, *P* ≤ 0.001) (*Figure 5D*) and haloperidol (up to 253.1 ± 134.0% vs. Vehicle for high risk and up to 194.2 ± 56% vs. Vehicle for low risk, *P* ≤ 0.001) (*Figure 5E*). Salbutamol decreased the RR interval of hiPSC-CMs carrying high-risk variants to a greater extent compared to those carrying low-risk variants (*Figure 5G*).

### 3.7 Gene-specific drug response differences between hiPSC-CMs carrying high-risk and low-risk P/LP variants

**Figure 6:**
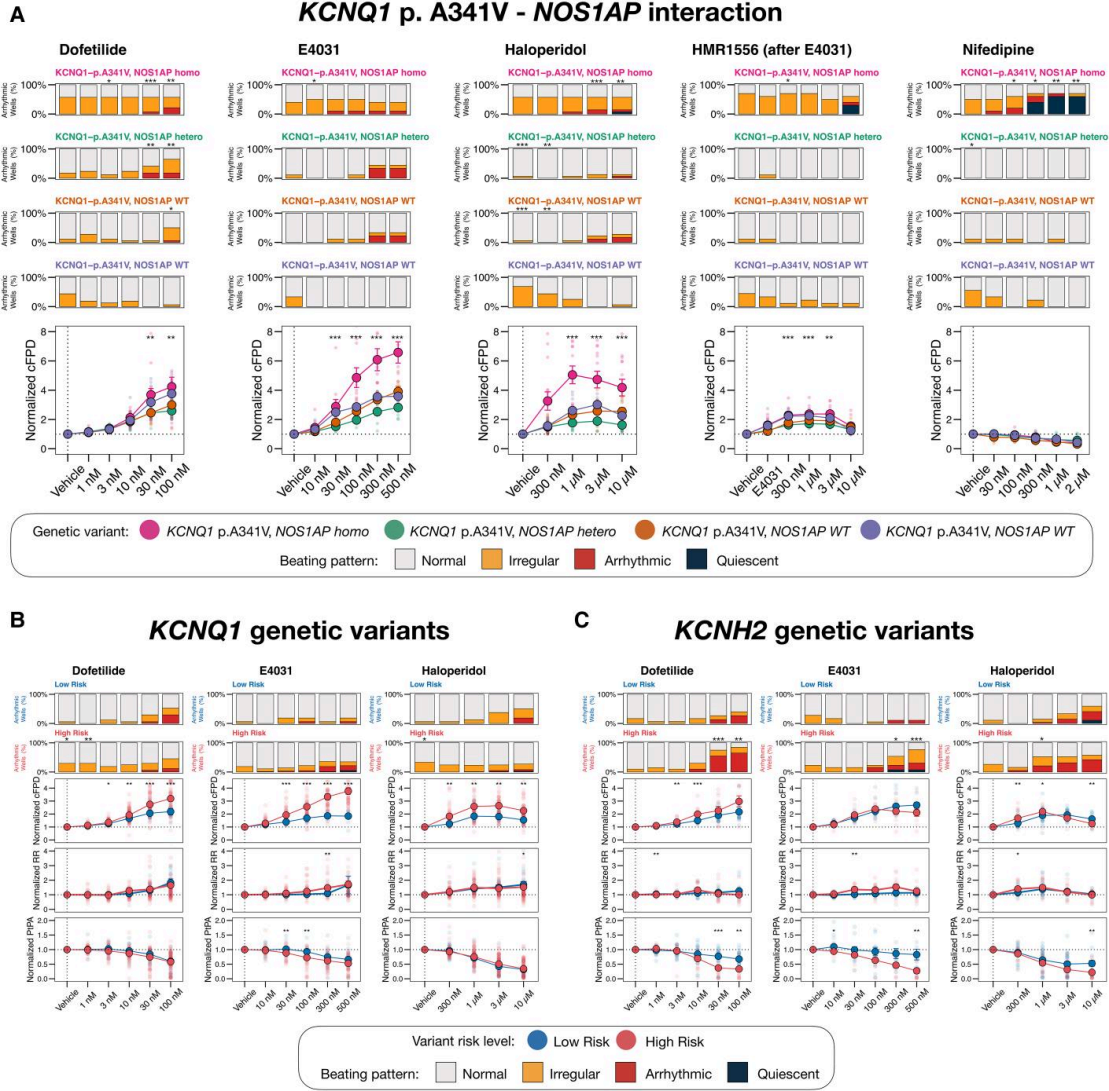
HiPSC-CMs carrying high-risk and low-risk variants demonstrate gene-specific drug responses. (*A*) Concentration-dependent response curves for dofetilide, E4031, haloperidol, HMR1556 following E4031 pre-treatment, and nifedipine in spontaneously beating hiPSC-CMs from four lines carrying the *KCNQ1* p.A341V variant in combination with either major (homozygous or heterozygous) or minor *NOS1AP* alleles. (*B*) Concentration-dependent drug effects on the electrophysiology of hiPSC-CMs carrying genetic variants in the *KCNQ1* gene. Readouts from hiPSC-CMs corresponding to the low-risk group (*KCNQ1* p.R190W) and the high-risk group (*KCNQ1* p.R594Q, *KCNQ1* p.R190W & p.R594Q, *KCNQ1* p.A341V) were pooled to create low-risk and high-risk cohorts. (*C*) Concentration-dependent drug effects on the electrophysiology of hiPSC-CMs carrying genetic variants in the *KCNH2* gene. Readouts from hiPSC-CMs corresponding to the low-risk group (*KCNH2* p.R366X) and the high-risk group (*KCNH2* p.A561V) were pooled to create low-risk and high-risk cohorts. Responses are shown for normalized corrected field potential duration (normalized cFPD), normalized RR interval (normalized RR), and normalized peak-to-peak amplitude (normalized PtPA). Bar plots illustrate the distribution of beating pattern abnormalities. Asterisks indicate statistically significant differences between cell lines/groups. *N* = 8-101 per line/group per drug concentration. Kruskal-Wallis test for multiple group comparison. Wilcoxon rank-sum test for two group comparison. Fisher’s exact test for categorical variables comparison. *P ≤ 0.05, **P ≤ 0.01, ***P ≤ 0.001.

To investigate whether gene-specific background influences drug response kinetics, we first focused on four hiPSC lines carrying the *KCNQ1* p.A341V variant, either alone or in combination with variants in the modifier gene *NOS1AP*. The lines included: (i) *KCNQ1* p.A341V, *NOS1AP* homo—*KCNQ1* p.A341V with homozygous *NOS1AP* minor allele variants (rs16847548 and rs4657139), (ii) *KCNQ1* p.A341V, *NOS1AP* hetero with heterozygous *NOS1AP* variants, and (iii and iv) *KCNQ1* p.A341V, *NOS1AP* WT homozygous for the major (wild-type) alleles of *NOS1AP*. Distinct drug response profiles were obtained among these lines (*Figure 6A*; Supplementary material online, *Table S8*). The *KCNQ1* p.A341V with homozygous *NOS1AP* minor allele variants exhibited significant cFPD prolongation and increased incidence of abnormal beating patterns following exposure to I_Kr_ and I_Ks_ blockers (dofetilide, E4031, haloperidol, HMR1556), along with increased sensitivity to the I_CaL_ blocker nifedipine. In contrast, the other three lines exhibited moderate responses to I_Kr_ and I_Ks_ blockade, and their beating pattern was not changed upon the nifedipine treatment.

In a separate analysis, we compared drug responses between high- and low-risk P/LP variants, separating hiPSC-CMs with *KCNQ1* variants (LQT1, JLNS) from those with *KCNH2* variants (LQT2). We analysed responses to I_Kr_-blocking drugs—dofetilide, E4031, and haloperidol—which demonstrated the strongest discrimination between high-risk and low-risk P/LP variants. Gene-specific features emerged in drug responses between hiPSC-CMs with high- and low-risk P/LP variants. High-risk hiPSC-CMs carrying *KCNQ1* variants showed significant FPD prolongation upon drug treatment (*Figure 6B*; Supplementary material online, *Table S9*), while, in contrast, high-risk *KCNH2* variant carriers demonstrated cFPD prolongation similar to the low-risk *KCNH2* group but exhibited a marked decrease in peak-to-peak amplitude and an increased incidence of abnormal beating patterns (*Figure 6C*; Supplementary material online, *Table S10*). Interestingly, when comparing concentration-response profiles, drug responses of WT hiPSC-CMs were similar to those observed in hiPSC-CMs carrying low-risk *KCNQ1* and *KCNH2* variants (Supplementary material online, *Figure S22*).

### 3.8 Machine learning model stratifies P/LP variant risk from hiPSC-CM multielectrode array data

**Figure 7:**
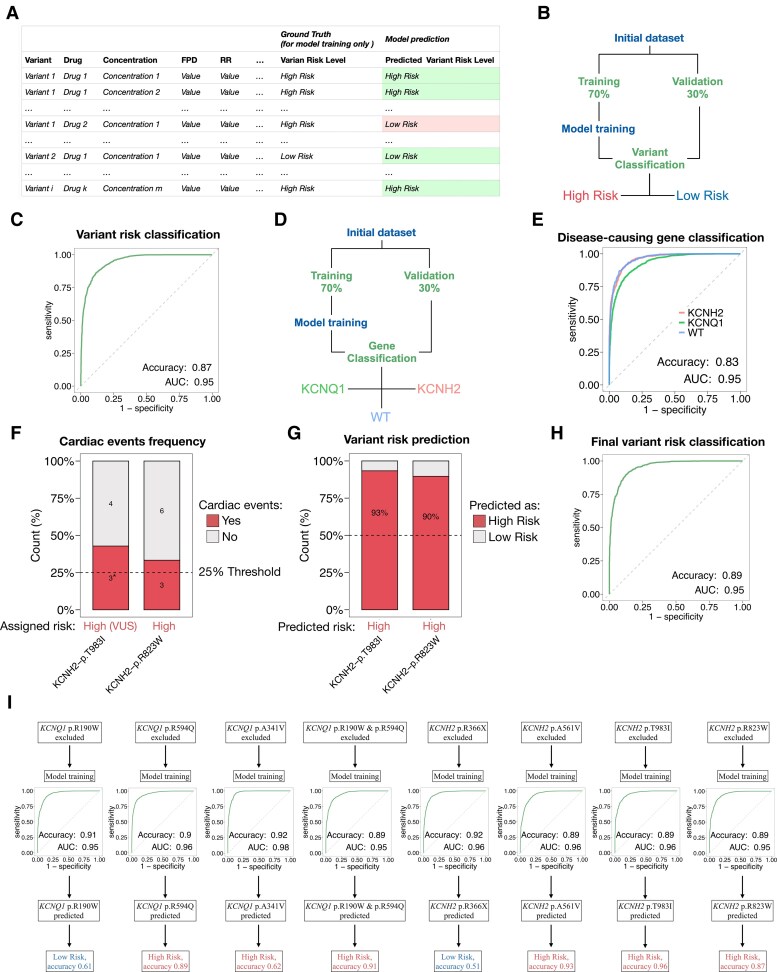
Variant risk levels and disease type prediction using MEA measurements and machine learning model. (*A*) Structure of the MEA readout dataset and example of row-by-row predictions of variant risk levels. (*B*) Variant risk prediction pipeline, indicating training and validation steps. (*C*) Receiver operating characteristic (ROC) curve illustrating the performance of a classification model for variant risk prediction on the primary dataset. (*D*) Disease-causing gene prediction pipeline. (*E*) ROC curves representing the model's performance for disease-causing gene prediction. (*F*) Percentage of patients with cardiac events for each variant in the validation cohort (*N* = 7-9), with assigned variant risk levels specified. Asterisk indicates inclusion of patients with mixed disease phenotypes. (*G*) Prediction of variant risk levels for the validation cohort using the model pretrained on the primary dataset. (*H*) ROC curve showing the performance of the classification model for variant risk prediction on the combined dataset incorporating all eight genetic variants and wild types. (*I*) Leave-one-out cross-validation results for single-variant risk level predictions. ROC curves are shown for each model following variant exclusion from the training set. The predicted risk level and individual prediction accuracy are indicated for each variant.

We curated a MEA dataset from 12 patient-specific hiPSC lines carrying 7 genetic variants, each exposed to a panel of 11 drugs. This primary dataset comprises 11 109 individual MEA readouts (*Figure 7A*). To train and evaluate a machine learning classifier for stratifying pathogenic/likely pathogenic (P/LP) variant risk based on *in vitro* drug responses, the dataset was randomly divided into training and testing subsets at a 70:30 ratio (*Figure 7B*). To ensure robust and unbiased predictions, we deliberately included all drug responses, even those from drugs that did not show apparent discrimination between high-risk and low-risk variants according to concentration-response curves. The classifier generated predictions for each individual readout (*Figure 7A*). The random forest model demonstrated robust classification performance with 87% accuracy and excellent discriminative ability, with an area under the receiver operating characteristic (ROC) curve (AUC) of 95% (*Figure 7C*). To assess whether the model could also identify gene-specific response patterns, we developed a separate classifier trained to distinguish among disease-causing genes (*Figure 7D*). This model demonstrated solid performance, with 83% classification accuracy and an AUC of 95% (*Figure 7E*).

### 3.9 Additional validation confirmed model accuracy for predicting individual variant risk

To further evaluate the generalization performance of the model and its ability to predict risk levels for previously unseen variants, we performed a series of validation tests. Because no publicly available datasets matched our experimental design, we generated an independent validation dataset using two LQT2 hiPSC lines carrying the *KCNH2* p.T983I and *KCNH2* p.R823W variants obtained from an independent hiPSC repository (Stanford Cardiovascular Institute, SCVI).

The *KCNH2* p.T983I line was derived from a symptomatic patient with a history of syncope and QTc prolongation, as well as a family history of SCD. Although this variant is classified as a variant of uncertain significance (VUS) according to ACMG criteria, its pathogenicity has been demonstrated using gene-edited iPSC-CMs.^59^ Additional reports have linked this variant to sudden cardiac death and atypical phenotypes, including mixed Andersen-Tawil syndrome and LQTS with severe arrhythmias and cardiac arrest.^64,65^ The P/LP *KCNH2* p.R823W variant line was derived from patients with a history of syncopal episodes and an ICD implanted. Given the high incidence of cardiac events associated with both variants, they were assigned to the high-risk category (*Figure 7F*).

The baseline electrophysiological properties and concentration-response profiles to I_Kr_-blocking drugs were in line with those previously observed in LQT2 hiPSC-CMs (Supplementary material online, *Figure S23*). The model was used to predict the risk level of the *KCNH2* p.T983I and *KCNH2* p.R823W variants, correctly classifying them both as high risk (*Figure 7G*). After having assessed the performance of our approach on a previously unseen cohort, data from these variants were added to the training dataset to create a model comprehensive of all the variants used in our study. The model performances were re-evaluated, demonstrating robust predictive capability with an overall classification accuracy of 89% and an area under the ROC curve (AUC) of 95% (*Figure 7H*). We also evaluated XGBoost and LightGBM as alternative machine learning algorithms to Random Forest, but both demonstrated marginally inferior predictive performance (Supplementary material online, *Figure S24*).

Finally, we performed leave-one-out cross-validation on the expanded dataset (13 133 rows), iteratively excluding each variant from training to assess model robustness and generalizability (*Figure 7I*). This approach simulates real-world classification for novel variants. Interestingly, when *KCNQ1* p.A341V or *KCNH2* p.R366X variants were excluded, the model’s accuracy, trained on the rest of the dataset, increased from 89 to 92%. This is an expected consequence of removing variants with high inter-patient variability in genetic background and disease severity.

When applied to predict the risk level of the held-out variants, the model correctly classified all eight variants into their appropriate risk categories (*Figure 7I*, *Table 3*). However, classification accuracy for the *KCNH2* p.R366X variant was 51%, indicating inconsistent predictions. To investigate this, we examined the drug-response profiles of individual *KCNH2* p.R366X hiPSC lines derived from one symptomatic and one asymptomatic patient (Supplementary material online, *Figure S25*). We observed that hiPSC-CMs from the symptomatic patient exhibited increased cFPD prolongation to I_Kr_ and I_Ks_ blockers, resembling the phenotype of hiPSC-CMs with high-risk variants. In contrast, cells from the asymptomatic patient showed a response comparable to low-risk variants.

**Table 3:** LQTS variant risk level prediction accuracy for each genetic variant included in the study

Variant	Disease	Variant risk level (ground truth)	Predicted variant risk level	Accuracy
KCNQ1-p.R190W	LQT1	Low risk	Low risk	0.61
KCNQ1-p.R594Q	LQT1	High risk	High risk	0.89
KCNQ1-p.A341V	LQT1	High risk	High risk	0.62
KCNQ1-p.R190W & p.R594Q	JLNS	High risk	High risk	0.91
KCNH2-p.R366X	LQT2	Low risk	Low risk	0.51
KCNH2-p.A561V	LQT2	High risk	High risk	0.93
KCNH2-p.T983I	LQT2	High risk	High risk	0.96
KCNH2-p.R823W	LQT2	High risk	High risk	0.87

## 4. Discussion

Risk stratification for LQTS patients remains a significant clinical challenge, particularly for carriers of ultra-rare P/LP variants or for those identified in a single proband. These shortcomings impact our ability to identify patients who are at low- or high-risk of developing cardiac events that could potentially lead to SCD. Here, we developed a machine learning pipeline to classify P/LP variant risk based on *in vitro* electrophysiological readouts from patient-specific hiPSC lines carrying genetic variants associated with varying frequencies of cardiac events (low risk or high risk).

Our findings support prior evidence demonstrating that hiPSC-CMs can effectively recapitulate the clinical phenotype of LQTS.^20–24^ This was particularly evident in hiPSC-CMs carrying severe genetic variants, such as compound heterozygous *KCNQ1* p.R190W & p.R594Q, *KCNQ1* p.A341V, and *KCNH2* p.A561V, where a clear prolongation in the repolarization duration was clearly observed *in vitro*.

We further strengthened the dataset beyond baseline measurements by challenging each variant with ion channel blockers and pro-arrhythmic compounds, thus creating variant-specific drug-response profiles not immediately obtainable in clinical practice. This is supported by previous research demonstrating excellent capacity of hiPSC-CMs to discriminate high/low responders to an acute sotalol administration^66^ or to tyrosine kinase inhibitors.^67,68^ To overcome the high baseline variability typical of hiPSC-CM measurements, often observed in cohort studies, we used relative readouts obtained from normalized drug responses to provide a more accurate discrimination between disease phenotypes. Indeed, high-risk variants were clearly discriminated from their exacerbated response to certain pro-arrhythmic drug treatments, leading to more severe changes in FPD, cFPD, and RR intervals or to a higher occurrence of beating pattern abnormalities.

The panel of pure ion channel blockers targeting four key ion channels responsible for action potential regulation revealed that pharmacological I_Kr_ blockade alone already provides effective variant risk discrimination. Conversely, treatment of hiPSC-CMs with the I_Ks_ blocker HMR1556 caused only minimal FPD prolongation.^69,70^ Reducing the repolarization reserve by pre-treating hiPSC-CMs with I_Kr_ blocker E4031^63,71^ increased the sensitivity to HMR1556 and discriminated between high-risk and low-risk groups. As expected, the I_CaL_ blocker nifedipine and the I_Na_ blocker tetrodotoxin were not effective in distinguishing high-risk from low-risk groups within our LQT1 and LQT2 cohort, which is expected since no pathogenic variants were identified in genes encoding for calcium and sodium channel subunits. These blockers could be useful for identifying Ca^2+^ or Na^+^ handling abnormalities in other LQTS subtypes beyond LQT1 and LQT2 (e.g. LQT3 or CALM-LQTS).^22,72^

Among the pro-arrhythmic drugs selected based on analyses of the OpenFDA and CredibleMeds databases, acute treatment of hiPSC-CMs with the potent I_Kr_ blocker dofetilide and the antipsychotic drug haloperidol demonstrated clear concentration-dependent cFPD prolongation, consistently distinguishing between the high-risk and low-risk groups. Acute treatment with salbutamol, a β_2_-adrenergic agonist commonly used as a bronchodilator and typically precluded to LQTS patients,^73^ differentially increased the spontaneous beating frequency in hiPSC-CMs; this allowed the discrimination of high-risk variants, which revealed a higher sensitivity, from low-risk variants and could be influenced by a difference in beating frequencies at baseline between these two groups. No arrhythmic events were observed, supporting previous studies on commercially available hiPSC-CMs.^74^ Treatment of hiPSC-CMs with ciprofloxacin, clarithromycin, and moxifloxacin within therapeutic concentration ranges resulted in moderate FPD prolongation, as similarly emerged in commercially available wild-type hiPSC-CMs.^31,75^

To investigate the influence of interactions between a disease-causing variant and a modifier gene on drug response, we included four hiPSC lines carrying the *KCNQ1* p.A341V variant, either alone or in combination with variants in the modifier gene *NOS1AP*.^52^ Previous studies reported that hiPSC-CMs carrying the *KCNQ1* p.A341V variant together with homozygous minor alleles in *NOS1AP* exhibit NOS1AP downregulation and increased peak L-type calcium current.^51^ Consistent with these findings, our study observed increased sensitivity to repolarization-targeting drugs and the L-type calcium channel block.

There is limited information available on gene-specific drug responses between LQT1 and LQT2, both *in vivo* and *in vitro*.^76–78^ In our study, three drugs with increased potency to block hERG (E4031, dofetilide, and haloperidol) well discriminated diseased hiPSC-CMs carrying high-risk variants in either *KCNQ1* or *KCNH2* genes. This observation aligns with findings from adult transgenic rabbit models, where LQT2 rabbits exhibited greater susceptibility to drug-induced TdP than LQT1 rabbits after administration of the anaesthetic propofol, with a known hERG channel blocking effect.^77^ These results are consistent with the concept of a severely compromised repolarization reserve in LQT2 cardiomyocytes, which renders them more vulnerable to pro-arrhythmic triggers.^71^

Variant risk prediction based on multiparametric readouts is a novel and rapidly developing field. A recent study combined clinical data, trafficking assays and automated patch clamp data from heterologous expression systems, to score the risk of *KCNH2* variants associated with LQT2.^79^ The use of hiPSC-CMs provides enhanced precision by addressing the limitations of heterologous systems that lack cardiac-specific context. Having demonstrated that hiPSC-CM drug responses effectively discriminate between high-risk and low-risk P/LP variants, we developed the ML model trained on *in vitro* hiPSC-CM electrophysiological readouts able to predict retrospectively the severity of variants with high precision—a methodology that, to our knowledge, has not been previously applied to LQTS. Importantly, the ML model trained for disease-causing gene discrimination predicted each gene with an accuracy of 83%. This robust performance indicates that disease-specific electrophysiological features are distinct from variant risk classification features, providing solid evidence to expand the model incorporating other LQTS subtypes.

The model successfully predicted risk levels for individual variants in an independent validation cohort not used during training, demonstrating robust validation and applicability to novel and previously unreported variants such as the *KCNH2* p.T983I or *KCNH2* p.R823W.

Incorporating a VUS into the validation dataset demonstrated the model’s versatility for potential prospective classification of patient-specific variant effects. The leave-one-out cross validations, where each individual variant was iteratively excluded from training and thus treated as never seen by the model, confirmed the robustness of the approach.

The reduced prediction consistency observed for the *KCNH2* p.R366X variant underscores the impact of patient-specific genetic backgrounds and drug responses. Although assigned as low risk due to a low cardiac event frequency (16.7%), two hiPSC lines from different LQT2 patients exhibited divergent drug response patterns. One line, derived from a patient with no cardiac events, aligned with low-risk variants, whereas the other, from a patient with documented cardiac events, markedly prolonged QTc (622 ms), and LSCD performed at age 30, with those of high risk. This highlights the importance of separating the genetic background from the primary disease-causing variant, particularly for P/LP variants that fall within the intermediate range of the risk level distribution. Indeed, in the case of high-risk *KCNQ1* p.A341V variant, despite differences in genetic background (such as *NOS1AP* genetic variants), the primary variant itself is so severe that the probability of misclassification is lower. These findings emphasize the necessity of integrating data from multiple sources for accurate variant risk classification, including multicentric *in vitro* data, clinical cohort records, structural variant pathogenicity predictions, and Bayesian penetrance estimates.^37^

In conclusion, we established a robust method for stratifying P/LP variant risk by integrating ML classification to electrophysiological recordings in patient-specific hiPSC-CMs. This approach enhances the risk stratification of LQTS variants beyond what is currently possible from clinical data alone, providing value for variants with limited clinical information.

Translational perspective *​​*Understanding which patients may be at risk of cardiac events or sudden cardiac death is crucial to implementing appropriate preventive measures. This study leverages patient-specific *in vitro* models and machine learning to improve the risk stratification of pathogenic/likely pathogenic variants associated with LQTS, better supporting clinical decisions related to risk assessment and management of LQTS patients. This scalable approach can be implemented across multiple centres, enhancing the risk stratification of LQTS variants beyond what is currently possible when clinical data are limited.

## 5. Study limitations

Our findings should be interpreted considering the following limitations. Experiments were performed on hiPSC-CMs, which exhibit an immature phenotype compared to adult cardiomyocytes.^80,81^ Although maturation protocols improved hiPSC-CM phenotypes, inherent differentiation-batch and line-to-line variability, as well as relative immaturity of ion channel function, persist; three-dimensional culture systems may further enhance maturity and electrophysiological stability and should be considered in future studies. This study did not use isogenic lines. While isogenic allelic series provide genetically normalized variant characterization, this approach may obscure the influence of each patient’s genetic background on arrhythmia susceptibility, features that contribute to incomplete penetrance and variable expressivity in LQTS and may be critical for patient-specific clinical decision-making. Indeed, the inter-individual variability in drug responses observed among carriers of identical variants in our cohort would not have been captured using isogenic models or traditional heterologous expression systems.

Although female lines were not available for all the variant combinations used in the study, sex distribution remained balanced between groups, with low-risk including three female and three male lines, and high-risk including five female and three male lines. In addition, the training dataset consisted of 3 low-risk and 4 or 6 high-risk variants, reflecting a modest class imbalance. This study focused on a limited number of genetic variants and hiPSC lines. Although our dataset represents one of the largest functional datasets derived from hiPSC-CMs to date and effectively demonstrates a proof of principle for retrospective variant risk stratification, further studies are needed to generalize this approach across a broader spectrum of variants and LQTS subtypes.

Another potential limitation of this study is that MEAs do not measure APD, as it would have been possible with other technologies. Although we recently observed strong correlations between FPD measured with MEAs and APD measured using organic transistors,^82,83^ this approach may not capture effects of genetic variants or drugs acting on parameters such as resting membrane potential or fractional APDs

Finally, while our model simplifies clinical risk into a binary classification, further evolutions of this approach could consider incorporating multiple classification states to accommodate intermediate disease phenotypes based on both qualitative and quantitative cardiac events associated with each variant.

## Supplementary Material

cvag105_Supplementary_Data

## Data Availability

The datasets, models and R code used for the analyses are available in the lab’s GitHub repository: https://github.com/invitroheart and on Zenodo: 10.5281/zenodo.20271907.
