# Supplementary material for: Machine learning-guided risk stratification for long QT syndrome genetic variants with hiPSC-derived cardiomyocytes
Source: Cardiovasc Res. 2026 May 14;122(10):1374–90. doi: 10.1093/cvr/cvag105 (PMC13355840; doi:10.1093/cvr/cvag105)
Supplement: cvag105_Supplementary_Data [file cvag105_supplementary_data.pdf]

Machine learning-guided risk stratification for Long QT Syndrome genetic variants with hiPSC-derived cardiomyocytes

Aleksandr Khudiakov PhD[a], Manuela Mura PhD[b], Federica Giannetti PhD[a], Vladislav Leonov PhD[a,c], Chiara Alberio[a], Marem Eskandr[a,d], Paola Adele Lonati[e], Maria Orietta Borghi[e,f], Paul A Brink MD[g], Lia Crotti MD, PhD[a,h], Massimiliano Gnecchi MD, PhD[b,i], Peter J Schwartz MD[a], Luca Sala PhD*[a,d]

Supplemental Appendix

Supplemental methods

A list of main reagents used in the study is reported in Supplemental Table 2.

hiPSC characterization

Karyotyping

HiPSCs were arrested in metaphase with colcemid (FUJIFILM) for 2 h, subjected to hypotonic treatment with 75 mM KCl, and fixed in fresh Carnoy's fixative (methanol:glacial acetic acid, 3:1). Chromosome identification was performed using Giemsa trypsin G-banding (GTG-banding), and karyotype description was made in accordance with the International System for Chromosome Nomenclature (ISCN, 2024).

Genetic variant analysis

Genomic DNA was extracted from hiPSCs with the MasterPure CompleteTM DNA & RNA purification kit (LGC Biosearch Technologies). The DNA regions of interest were amplified by PCR using primers listed in Supplemental Table 11 and Phire Green Hot Start II PCR Master Mix (Thermo Fisher Scientific), following manufacturer's instructions, purified with

the Expin Combo GP kit (GeneAll), and sequenced by Eurofins Genomics (LightRun service).

Immunofluorescence for pluripotency markers

HiPSCs seeded on vitronectin-coated coverslips, were fixed for 15 minutes with 4% paraformaldehyde in 1× DPBS, permeabilized for 5 minutes with 0.1% Triton X-100, and blocked for 1 hour at room temperature (RT) in 1% BSA. Primary antibodies (Supplemental Table 12) were incubated overnight at 4°C. Then cells were washed three times in DPBS, and incubated with secondary antibodies for 1 h at RT. Nuclei were counterstained with Hoechst 33258 (1 µg/mL) and coverslips were mounted with ProLong Glass Antifade Mountant (Thermo Fisher Scientific). Images were acquired on a Zeiss Axio Observer.Z1 microscope equipped with the ApoTome system using AxioVision 6.0.

Alkaline phosphatase colorimetric assay

Alkaline phosphatase (AP) activity was evaluated with the Alkaline Phosphatase Staining Kit II (Stemgent), following manufacturer's instructions.

HiPSC cardiac differentiation

Cardiac differentiation was started 2-4 days after hiPSC plating, when hiPSCs reached 70-90% confluency. At this stage (Day 0), the culture medium was switched to RBins- medium, which consists of RPMI1640 supplemented with 1% of B27 supplement without insulin (Gibco), and 6 µM CHIR99021 (Selleckchem) was added to induce the differentiation. For the next two days, fresh RBins- medium was added (⅔ of the initial volume on day 1 and ⅓ on day 2). On day 3, the medium was switched to RBins- medium supplemented with 5 µM IWR1 (Merck). After two days of treatment with IWR1, the medium was replaced with RBK medium, consisting of RPMI1640 supplemented with 1% B27 Supplement (Gibco) and 1% Knock-Out Serum Replacement (Gibco). Starting from day

7 hiPSC-CMs were purified through glucose starvation [1] and cryopreserved in Bambanker (Nippon Genetics) at days 9-16. For the subsequent experiments hiPSC-CMs were thawed, replated at low density and expanded as previously reported [2]. Data were collected from at least three independent differentiations of each hiPSC line for each experiment.

Flow cytometry quantification of hiPSC-CM purity

To quantify the percentage of cardiac troponin T (cTnT)-positive cells, expanded cardiomyocytes were dissociated with TrypLE Select Enzyme (10X) (Gibco) and diluted to $1 * 10^6$ cells/mL in DPBS containing 5% fetal bovine serum (FBS). To identify dead cells, cardiomyocytes were incubated in the dark for 15 minutes at room temperature with Viobility fixable dye 488/520 (Miltenyi Biotec). Cells were then fixed using Cytofix solution (BD Biosciences) and permeabilized with Perm/Wash buffer (BD Biosciences) according to the manufacturer's instructions. Subsequently, cells were stained in the dark with VioBlue anti-human cTnT antibody (Miltenyi Biotec) for 10 minutes at room temperature. Prior to acquisition, cardiomyocytes were resuspended in a PEB buffer and filtered through a 100 µm cell strainer. Data acquisition was performed using a FACSLyric flow cytometer (BD Biosciences), and analysis was carried out with FACSuite software (BD Biosciences). A total of 10,000 live cells were acquired at a medium flow rate. The percentage of cTnT-positive cells was calculated by subtracting the background fluorescence of unstained control viable cells from the percentage of viable cTnT-positive cells.

Patch-clamp current-clamp and voltage-clamp recordings

Expanded hiPSC-CMs were dissociated with TrypLE Select Enzyme (10X) (Gibco) and plated in monolayers at a density $3 * 10^5/cm^2$ on 12-well plates coated with Matrigel

(Corning) in RBK medium. The next day, RBK was replaced with a lipid-rich maturation medium (MM) [3]. Metabolic maturation was performed for two weeks and MM was refreshed twice a week. For action potential (AP) recordings, hiPSC-CMs were dissociated with TrypLE Select 10X (Gibco) and plated on Matrigel (Corning) coated glass coverslips as single cells with a density of 8.4 - 10.5 * $10^3/cm^2$.

Action potential recording

APs were recorded in perforated patch mode 3-10 days after hiPSC-CMs dissociation. The extracellular solution was based on modified Tyrode's solution and contained (mM): NaCl 154, KCl 5.4, CaCl2 1.8, HEPES-NaOH 5, D-Glucose 5.5. pH was set to 7.35 with NaOH. The intracellular solution contained (mM): K-Aspartate 125, KCl 20, NaCl 10, Na2-ATP 5, HEPES 10. pH was set to 7.3 with KOH. Amphotericin B 0.22 mM, dissolved in DMSO, was added to the intracellular solution to record APs.

Current clamp protocol to record APs was performed with a Molecular Devices digidata 1440A and a Molecular Devices Axopatch 200B amplifier at physiological temperature (37 °C) and under 1 Hz pacing. Current pulses with duration of 1.8-2.5 ms and amplitudes of 0.3-1.2 nA were used to elicit APs. After achieving a gigaOhm seal and allowing access resistance to stabilize within 15-20 MΩ, spontaneous electrical activity was recorded, when present. Electrical pacing at the slowest frequency is then initiated by stimulating the cell through the patch clamp pipette with a square current pulse of 1.8 - 2.5 ms of duration and 0.3 - 1.2 nA of amplitude. Once a steady state is reached at a certain frequency, we switch to a faster one, eventually adjusting the stimulus parameters as needed to be compliant with cell adaptation. APs were digitized at 5 kHz and filtered at 2 kHz with a low-pass Bessel filter. Liquid junction potential was calculated according to the stationary Nernst-Planck equation using LJPcalc (Harden, SW and Brogioli, D (2020). LJPcalc [Online]. Available:

https://swharden.com/software/LJPcalc, Accessed on 03/08/2021). The calculated LJP was 13.922 mV. The measured LJP was -11.2 ± 0.9 mV.

Slow delayed rectifier potassium current recording

The slow delayed rectifier potassium current (I_{Ks}) was recorded with the patch clamp technique in isolated hiPSC-CMs. Signals were digitized at 2 kHz and filtered at 1 kHz through a Molecular Devices 1440A Digidata connected to a Molecular Devices 200B amplifier. Currents were recorded at physiological temperature (37 °C). The extracellular solution contained (mM): NaCl 154, KCl 5.4, CaCl2 1.8, HEPES-NaOH 5, D-Glucose 5.5; pH was set to 7.35 with NaOH. The intracellular solutions for I_{Ks} recordings contained (mM): K-Aspartate 110, KCl 23, MgCl2 3, EGTA-KOH 5, HEPES-KOH 5, Guanosine-5'-triphosphate disodium salt 0.4, Adenosine-5'-triphosphate disodium salt 5, Phosphatidylcholine sodium salt 5, and CaCl2 2. Currents were evoked from a holding potential of -40 mV, with depolarizing voltage steps of 4000 ms every 10 mV up to +60 mV, followed by 8000 ms at -40 mV. I_{Ks} was isolated as 1 µM HMR-1556 (Selleckchem) sensitive current, in the presence of 1 µM of the I_{Kr} blocker E4031 dihydrochloride (Selleckchem) and 1 µM of the L-type calcium channel blocker nifedipine (Merck). pClamp 10.4 was used to record the traces and Clampfit 10 was used for the analyses.

MultiElectrode Arrays

Multiwell MEAs (24 wells, MultiChannel Systems) were coated with 40 µg/mL bovine fibronectin (Merck) for 1h at 37 °C and processed as previously described [4,5]. Expanded hiPSC-CMs were dissociated with TrypLE Select Enzyme 10X (Gibco) and plated on the electrodes of Multiwell MEAs at a density of 5 * 10^4 hiPSC-CMs per well in small drops. RBK medium supplemented with Revitacell (Gibco) was used for plating. The next day, and

thereafter twice a week, half of the medium was refreshed with RBK medium. After two weeks, baseline recordings were performed followed by acute drug treatment and cumulative concentration-response measurements.

Quality control

Quality control of multi-electrode array (MEA) recordings was performed over multiple days by assessing the presence of detectable electrical activity starting as early as two days after hiPSC-CM seeding and continuing until the day of recording. Following plating, MEA plates were monitored twice per week to verify the consistency and progressive improvement of electrical signals over time, including signal amplitude, signal-to-noise ratio, and beating frequency. Wells exhibiting field potential signals with a peak-to-peak amplitude of less than 30 µV were excluded from further analysis. In addition, wells were excluded if key field potential (FP) parameters could not be reliably detected or if the beat rate was extremely slow (RR interval > 30 s).

Drug testing

Two weeks after hiPSC-CM seeding, baseline recordings were obtained, followed by acute drug exposure and cumulative concentration-response measurements. On the day preceding the experiment, the medium volume in each well of the MEA plate was measured and adjusted to 1 mL.The drug testing protocol included the following steps: 1) recording of baseline Field Potentials (FP), 2) addition of vehicle (RBK) medium to mimic temperature and mechanical impact of drug addiction 3) addition of cumulative concentrations of compounds . All the recordings simulated acute drug exposure and consisted in 1 minute of wash-in followed by two minutes of recording. Timelines of drug exposures and drug administration were controlled by a software tool integrated in the MEA software suite (Multichannel Systems Multiwell Screen). Drug additions were performed by two operators, each responsible for half of the plate (12 wells), using 20 µL injections with annually

calibrated Eppendorf pipettes. Aliquots of medium, vehicle, and drugs were maintained under controlled incubator conditions (37 °C, 5% CO_2) until use. Incremental drug concentrations were calculated accounting for the cumulative increase in well volume due to successive 20 µL additions. The following drugs were tested: chlorpromazine (Selleckchem), ciprofloxacin hydrochloride hydrate (Selleckchem), clarithromycin (Selleckchem), dofetilide (Selleckchem), E4031 dihydrochloride (Selleckchem), haloperidol (Selleckchem), HMR-1556 (Selleckchem), moxifloxacin (Selleckchem), nifedipine (Merck), salbutamol sulfate (Selleckchem), tetrodotoxin citrate (Tocris).

Field Potential analysis

MEA recordings were analyzed using Multiwell-Analyzer v2.0.6.0 (Multichannel Systems) and the following parameters were extracted: field potential duration (FPD), beat-to-beat interval (RR), peak-to-peak amplitude (PtPA), mean slope of depolarization (Mean slope), RR interval coefficient of variation (RRCV). The analysis of FPs was performed as previously described [4] and automated through custom R scripts (R v4.3.1). FPD was corrected for beating frequency (cFPD) using Bazett's formula.

Field Potential quality assessment

The quality of the FP was assessed with a scoring system as previously described [5] and then further stratified to four categories based on the beating patterns: normal beating (scores 3-5), irregular beating (score 2), arrhythmic beating (score 1), and quiescence (score 0). This was implemented with the rationale of separating drug effects on the quantitative properties of the FP (e.g. a FP prolongation) from the potential detrimental effects of the drug treatment on FP quality as often no FPD was measured from recordings scored as 1 and 0. The representative examples of each beating pattern are presented in Supplemental Figure 5.

Supplemental results

Generation and characterization of hiPSC lines

The wild-type line S34Ec16 was generated from PBMCs using the CytoTune-iPS 2.0 Sendai Reprogramming Kit (Thermo Fisher Scientific), according to the manufacturer's recommendations. Lines SA14.19 and SA15.14, carrying the *KCNQ1* A341V genetic variant, were generated from dermal fibroblasts via retroviral infection with *OCT4*, *SOX2*, *KLF4*, and *cMYC* and fully characterized around passage 10, following the procedure described previously [6,7]. Line 26.1, carrying the *KCNH2* R366X genetic variant, was generated from dermal fibroblasts through transfection with two oriP/EBNA1-based episomal plasmids and fully characterized too around passage 10, following the previously described procedure [8]. All the other ten hiPSCs were already available. Their generation/characterization is reported in the references of the Supplemental Table 1.

Before starting cardiac differentiation, all the hiPSC lines studied in the present study have been characterized again in order to confirm genetic stability and maintenance of pluripotency (Supplemental Figures 1-5). All the 14 hiPSC lines presented a diploid karyotype: 46;XY for WTC-11, SC34Ec16 (Supplemental Figure 1A), 28.44 (Supplemental Figure 2A), SA15.14 (Supplemental Figure 3A), SCVI498c1 and SCVI209c1 (Supplemental Figure 5A), and 46;XX for 29.7, 30.3 (Supplemental Figure 2A), SA13.5, SA14.19, Sa6.27 (Supplemental Figure 3A), 26.1, 27.2 (Supplemental Figure 4A), SCVI209c1 (Supplemental Figure 5A).

The two wild-type lines WTC-11 and SC34Ec16 do not carry any of the 8 LQTS-causing variants included in this study (Supplemental Figure 1B). 28.44 hiPSCs carry the *KCNQ1* 1781 G/A variant in heterozygosis, whereas 29.7 hiPSCs carry the *KCNQ1* 568 C/T variant in heterozygosis. 30.3 hiPSCs from the JLNS patient carry both the *KCNQ1* 568 C/T and 1781

G/A variants in heterozygosis (Supplemental Figure 2B). All the four South African LQT1 family hiPSC lines SA13.5, SA6.27, SA14.19, SA15.14 carry the *KCNQ1* 1022 C/T variant in heterozygosis (Supplemental Figure 3B). SA13.5 hiPSCs carry the *NOS1AP* rs16847548 minor allele in homozygosis, whereas SA14.19 hiPSCs are heterozygous for the minor allele. SA6.27 and SA15.14 hiPSCs are both homozygous for *NOS1AP* rs16847548 major allele (Supplemental Figure 3B).

26.1 and 27.2 LQT2 hiPSCs carry the *KCNH2* 1096 C/T variant in heterozygosis (Supplemental Figure 4B). Also the other three LQT2 hiPSCs carry the disease-causing variants in heterozygosis: *KCNH2* 1682 C/T variant in SCVI498c1 hiPSCs, *KCNH2* 2467 C/T in SCV507ci hiPSC, and *KCNH2* 2948 C/T variant in SCVI209c1 hiPSCs (Supplemental Figure 5B).

All the 14 hiPSC lines uniformly express the human embryonic stem cell surface antigens Tumor Related Antigen-1-60 (TRA-1-60), Stage Specific Embryonic Antigen-3 and -4 (SSEA-3, SSEA-4), and the pluripotent nuclear transcription factors NANOG, OCT4 and SOX2 (Supplemental Figures 1C, 2C, 3C, 4C and 5C). They also show alkaline phosphatase (AP) activity (Supplemental Figures 1D, 2D, 3D, 4D and 5D).

Flow cytometry quantification of hiPSC-CM purity

To confirm hiPSC-CM purity, flow cytometry analysis was performed to evaluate the percentage of cardiac troponin T (cTnT)-positive cells. Across six independent differentiations of three randomly selected hiPSC-CM, the mean percentage of cTnT-positive cells was >90% (Supplemental Figure 6).

Vehicle effects on electrophysiological readouts

To confirm that vehicle exposure doesn't affect electrophysiological measurements, we evaluated the impact of cumulative treatment with DMSO and ethanol in wild-type (WT1) and *KCNH2* p.A561V hiPSC-CMs (Supplemental Figure 26). Concentration-response analyses showed that vehicle controls induced minimal changes in normalized field potential duration (FPD), RR interval, and peak-to-peak amplitude (PtPA). Statistically significant differences were observed between drug-treated and corresponding vehicle-treated groups, indicating that the electrophysiological effects were driven by drug-induced effects rather than vehicle exposure.

Time-dependent stability of electrophysiological measurements

We next assessed potential time-dependent effects on electrophysiological readouts using time-course experiments analyzed at baseline, three minutes, and ten minutes after drug injection (Supplemental Figure 27). Across all tested conditions, including untreated controls, medium control (RBK), ethanol, and DMSO, electrophysiological parameters remained largely stable over time in both wild-type (WT1) and *KCNH2* p.A561V hiPSC-CMs. A modest increase in beating frequency (decrease in the RR interval) was observed during the measurement period, likely related to the time the MEA plate spent outside the CO_2 incubator environment. Comparisons between drugs and their respective vehicles revealed minimal time-dependent changes, supporting the temporal stability of the recorded electrophysiological signals.

Absence of well-specific effects in multiwell MEA recordings

To evaluate whether well position influenced electrophysiological measurements, we analyzed field potential duration (FPD), RR interval, and corrected FPD (cFPD) across

individual wells of the 24-well multiwell MEA (Supplemental Figure 28). Measurements were consistent across wells, with no systematic differences observed between inner and outer well positions. Aggregated analyses confirmed the absence of biologically meaningful well-specific effects, although cFPD values showed statistically significant differences likely due to the high number of measurements, these were small and not biologically relevant. Together these results indicate that well location did not confound electrophysiological readouts in the experimental setup.

Supplemental tables

Supplemental Table 1. List of hiPSC lines used in the study and their characterization status.

	Genetic variant	Known modifier gene	Disease	Line name	Line ID	Reference
1	Wild-type (WT1)		Healthy	WTC-11		[9], Supplemental Figure 1
2	Wild-type (WT2)		Healthy	S34Ec16		Supplemental Figure 1
3	*KCNQ1* p.R190W		LQT1	29.7	PSMi005-A	[10], Supplemental Figure 2
4	*KCNQ1* p.R594Q		LQT1	28.44	PSMi004-A	[11], Supplemental Figure 2
5	*KCNQ1* p.A341V		LQT1	SA6.27	PSMi001-A	[12], Supplemental Figure 3
6	*KCNQ1* p.A341V		LQT1	SA15.14		Supplemental Figure 3
7	*KCNQ*1 p.A341V	*NOS1AP* rs16847548 and rs4657139 heterozygous minor alleles	LQT1	SA14.19		Supplemental Figure 3
8	*KCNQ1* p.A341V	*NOS1AP* rs16847548 and rs4657139 homozygous minor alleles	LQT1	SA13.5	PSMi007-A	[7,12], Supplemental Figure 3
9	*KCNQ1* p.R190W & *KCNQ1* p.R594Q		JLNS	30.3	PSMi002-A	[13], Supplemental Figure 2
10	*KCNH2* p.R366X		LQT2	26.1		Supplemental Figure 4
11	*KCNH2* p.R366X		LQT2	27.2		[8], Supplemental Figure 4
12	*KCNH2* p.A561V		LQT2	SCVI498c1		[14], Supplemental Figure 5
13	*KCNH2* p.T983I		LQT2	SCVI209c1		Supplemental Figure 5
14	*KCNH2* p.R823W		LQT2	SCVI507c1		Supplemental Figure 5

Supplemental Table 2. List of main reagents used in the study.

Reagent	Company	Product code
B-27™ Supplement	Thermo Fisher Scientific [GIBCO]	17504044
B-27™ Supplement, minus insulin	Thermo Fisher Scientific [GIBCO]	A1895601
Bambanker	Nippon Genetics	BB03
CHIR-99021 HCl	Selleckchem	S2924
Chlorpromazine	SelleckChem	#S5749
Ciprofloxacin hydrochloride hydrate	SelleckChem	#S5208
Clarithromycin	SelleckChem	#S2555
Dofetilide	SelleckChem	#S1658
DPBS, no calcium, no magnesium	Thermo Fisher Scientific [GIBCO]	14190094
E4031 dihydrochloride	Tocris	#1808
EDTA (0.5 M), pH 8.0, RNase-free	Thermo Fisher Scientific [Invitrogen]	AM9262
Essential 8™ Flex Medium Kit	Thermo Fisher Scientific [GIBCO]	A2858501
Fibronectin bovine plasma	Merck [Sigma-Aldrich]	F1141
Haloperidol	SelleckChem	#S1920
HMR1556	Tocris	#5011
IWR-1	Merck [Sigma-Aldrich]	I0161
KOSR	Thermo Fisher Scientific [GIBCO]	10828028
Lactic acid	Merck	27714
Matrigel® Corning® hESC-Qualified Matrix, LDEV-free	Corning	354277
Moxifloxacin	SelleckChem	#S5535
Nifedipine	Merck	#N7634
RevitaCell™ Supplement	Thermo Fisher Scientific [GIBCO]	A2644501
RPMI 1640 Medium, no glucose	Thermo Fisher Scientific [GIBCO]	11879020
RPMI 1640 w/ L-Glutamine	Euroclone	ECB2000
Salbutamol	SelleckChem	#S2507
Tetrodotoxin citrate	Tocris	#1069
TrypLE™ Select Enzyme	Thermo Fisher Scientific [GIBCO]	A1217701
Vitronectin (VTN-N) Recombinant Human Protein, Truncated	Thermo Fisher Scientific [GIBCO]	A14700

Supplemental Table 3. List of pro-arrhythmic compounds in the CredibleMeds® database associated with specific OpenFDA queries.

Drug	Drug Class	Therapeutic Use	Classification on CredibleMeds
albuterol (salbutamol)	Bronchodilator	Asthma	Avoid in congenital long QT
amiodarone	Antiarrhythmic	Arrhythmia	Risk of TdPAnd Avoid in congenital long QT
amitriptyline	Antidepressant, Tricyclic	Depression	Conditional Risk of TdPAnd Avoid in congenital long QT
amphotericin b	Antifungal	Fungal infection	Conditional Risk of TdPAnd Avoid in congenital long QT
aripiprazole	Antipsychotic, atypical	Schizophrenia, depression (adjunct)	Possible Risk of TdPAnd Avoid in congenital long QT
chlorpromazine	Antipsychotic, antiemetic	Schizophrenia, bipolar disorder, acute psychosis	Risk of TdPAnd Avoid in congenital long QT
cimetidine	H2-receptor antagonist	Gastric hyperacidity, GERD	Conditional Risk of TdPAnd Avoid in congenital long QT
ciprofloxacin	Antibiotic	Bacterial infection	Risk of TdPAnd Avoid in congenital long QT
clarithromycin	Antibiotic	Bacterial infection	Risk of TdPAnd Avoid in congenital long QT
clofazimine	Antibiotic	Leprosy	Possible Risk of TdPAnd Avoid in congenital long QT
clozapine	Antipsychotic, atypical	Schizophrenia	Possible Risk of TdPAnd Avoid in congenital long QT
diphenhydramine	Antihistamine	Allergic rhinitis, insomnia	Conditional Risk of TdPAnd Avoid in congenital long QT
dofetilide	Antiarrhythmic	Arrhythmia	Risk of TdPAnd Avoid in congenital long QT

donepezil	Cholinesterase inhibitor	Dementia (Alzheimer's Disease)	Risk of TdPAnd Avoid in congenital long QT
famotidine	H2-receptor antagonist	Gastric hyperacidity, GERD	Conditional Risk of TdPAnd Avoid in congenital long QT
flecainide	Antiarrhythmic	Arrhythmia	Risk of TdPAnd Avoid in congenital long QT
fluconazole	Antifungal	Fungal infection	Risk of TdPAnd Avoid in congenital long QT
fluorouracil	Anti-cancer	Cancer	Possible Risk of TdPAnd Avoid in congenital long QT
furosemide	Diuretic	Hypertension, diuresis	Conditional Risk of TdPAnd Avoid in congenital long QT
haloperidol	Antipsychotic	Schizophrenia, agitation	Risk of TdPAnd Avoid in congenital long QT
hydrochlorothiazide	Diuretic	Hypertension, diuresis	Conditional Risk of TdPAnd Avoid in congenital long QT
hydroxychloroquine	Antimalarial, Anti-inflammatory	Malaria, SLE, rheumatoid arthritis	Conditional Risk of TdPAnd Avoid in congenital long QT
indapamide	Diuretic	Hypertension, diuresis	Conditional Risk of TdPAnd Avoid in congenital long QT
ivabradine	Antianginal	Angina Pectoris (heart pain)	Conditional Risk of TdPAnd Avoid in congenital long QT
lansoprazole	Proton Pump Inhibitor	Gastric hyperacidity, GERD	Conditional Risk of TdPAnd Avoid in congenital long QT
levofloxacin	Antibiotic	Bacterial infection	Risk of TdPAnd Avoid in congenital long QT
loperamide	Opiate	Diarrhea	Conditional Risk of TdPAnd Avoid in congenital long QT
metoclopramide	Antiemetic	Nausea, vomiting	Conditional Risk of TdPAnd Avoid in congenital long QT

metronidazole	Antibiotic	Trichomoniasis, amebiasis, bacterial infection	Conditional Risk of TdPAnd Avoid in congenital long QT
mirtazapine	Antidepressant, Tetracyclic	Depression	Possible Risk of TdPAnd Avoid in congenital long QT
moxifloxacin	Antibiotic	Bacterial infection	Risk of TdPAnd Avoid in congenital long QT
nilotinib	Anti-cancer	Cancer (leukemia)	Possible Risk of TdPAnd Avoid in congenital long QT
olanzapine	Antipsychotic, atypical	Schizophrenia, bipolar disorder	Conditional Risk of TdPAnd Avoid in congenital long QT
omeprazole	Proton Pump Inhibitor	Gastric hyperacidity, GERD	Conditional Risk of TdPAnd Avoid in congenital long QT
ondansetron	Antiemetic	Nausea, vomiting	Risk of TdPAnd Avoid in congenital long QT
pantoprazole	Proton Pump Inhibitor	Gastric hyperacidity, GERD	Conditional Risk of TdPAnd Avoid in congenital long QT
propofol	Anesthetic, general	Anesthesia	Risk of TdPAnd Avoid in congenital long QT
quetiapine	Antipsychotic, atypical	Schizophrenia	Conditional Risk of TdPAnd Avoid in congenital long QT
risperidone	Antipsychotic, atypical	Schizophrenia	Possible Risk of TdPAnd Avoid in congenital long QT
sotalol	Antiarrhythmic	Arrhythmia	Risk of TdPAnd Avoid in congenital long QT
sulpiride	Antipsychotic, atypical	Schizophrenia	Risk of TdPAnd Avoid in congenital long QT
tacrolimus	Immunosuppressant	Immune suppression	Possible Risk of TdPAnd Avoid in congenital long QT
torsemide	Diuretic	Hypertension, diuresis	Possible Risk of TdPAnd Avoid in congenital long QT

tramadol	Analgesic	Pain	Possible Risk of TdPAnd Avoid in congenital long QT
voriconazole	Antifungal	Fungal infection	Conditional Risk of TdPAnd Avoid in congenital long QT

Compounds selected for the study are highlighted in pink.

Supplemental Table 4. Ion channel blockers and pro-arrhythmic compounds used in the study.

Compound	Drug class	Therapeutic use	IC50 HERG	Cmax	Tested concentration range	Stock concentration	Vehicle
Chlorpromazine	Antipsychotic, antiemetic	Schizophrenia, bipolar disorder, acute psychosis	21.6 µM [15]	0.0345 µM [16]	0.03 - 3 µM	100 mM	DMSO
Ciprofloxacin	Antibiotic	Bacterial infection	966 µM [17]	8.87 µM [18]	3 - 30 µM	15 mM	DMSO
Clarithromycin	Antibiotic	Bacterial infection	45.7 µM [19]	1.206 µM [16]	1 - 10 µM	6 mM	DMSO
Dofetilide	Antiarrhythmic	Arrhythmia	0.007 µM [20]	0.0023 µM [16]	1 - 100 nM	50 mM	DMSO
E4031	Selective I_{Kr} blocker	-	0.0077 µM [21]	-	10 - 500 nM	10 mM	Water
Haloperidol	Antipsychotic	Schizophrenia, agitation	1 µM [22]	0.079 µM [23]	0.3 - 10 µM	150 mM	DMSO
HMR1556	Selective I_{Ks} blocker	-	-	-	0.3 - 10 µM	10 mM	DMSO
Moxifloxacin	Antibiotic	Bacterial infection	41.2 µM [24]	11.7 µM [25]	1 - 100 µM	100 mM	DMSO
Nifedipine	Selective I_{CaL} blocker	Angina, high blood pressure	-	0.0077 µM [16]	0.03 - 2 µM	10 mM	Ethanol, 100%
Salbutamol (albuterol)	Bronchodilator	Asthma	-	0.059 µM [26]	0.003 - 0.3 µM	100 mM	Water
Tetrodotoxin	Selective I_{Na} blocker	-	-	-	0.1 - 30 µM	3.13 mM	Water

Supplemental Table 5. Mean patients' QTc values and mean Schwartz scores for each genetic variant included in the study.

Variant	N	QTc (ms)	Schwartz Score
KCNQ1-p.R190W	102	464.7 ± 38	2.9 ± 1.5
KCNQ1-p.R594Q	16	491.9 ± 61.3	3.2 ± 2.2
KCNQ1-p.R190W & p.R594Q	1	578	7.5
KCNQ1-p.A341V	183	489.9 ± 45.8	4.7 ± 1.6
KCNH2-p.R366X	12	501.2 ± 56.9	4.5 ± 1.9
KCNH2-p.A561V	13	543.4 ± 34.6	5.0 ± 1.0
KCNH2-p.T983I	-	-	-
KCNH2-p.R823W	5	492.7 ± 44.8	5.3 ± 2.1

Data are presented as mean ± standard deviation. N indicates the number of patients included in the analysis.

Supplemental Table 6. Genetic variants selected for the study and their functional characterisation status.

Genetic variant	Disease	dbSNP record	Protein localization	ClinVar classification	Functional characterization	Reference
KCNQ1 p.R190W	LQT1	rs199473662	S2-S3 loop	Pathogenic/Likely pathogenic	This study	
KCNQ1 p.R594Q	LQT1	rs199472815	C-terminal domain	Pathogenic/Likely pathogenic	loss of function, trafficking defect	27
KCNQ1 p.A341V	LQT1	rs12720459	S6 segment	Pathogenic	loss of function	28–30
KCNQ1 p.R190W & *KCNQ1* p.R594Q	JLNS	rs199473662, rs199472815	S2-S3 loop and C-terminus	-	This study	
KCNH2 p.R366X	LQT2	rs794728364	N-terminal domain	Pathogenic	FPD prolongation (iPSC-CMs)	8
KCNH2 p.A561V	LQT2	rs121912504	S5 segment	Pathogenic/Likely pathogenic	loss of function, trafficking defect	14,31–34
KCNH2 p.T983I	LQT2	rs149955375	C-terminal domain	VUS	loss of function	35
KCNH2 p.R823W	LQT2	rs199473538	C-terminal domain	Pathogenic/Likely pathogenic	loss of function, trafficking defect	32,36–38

Supplemental Table 7. Differential response of Low Risk and High Risk hiPSC-CMs to ion channel blockers and proarrhythmic drugs.

Variant Risk Level	Drug	Concentration	N	Normalized FPD (% of Vehicle)	Normalized cFPD (% of Vehicle)	Normalized RR (% of Vehicle)	Normalized PtPA (% of Vehicle)
Low Risk	Chlorpromazine	30 nM	41	107.8 ± 6.7, p = 0.07	112.2 ± 14.8, p = 0.86	94.3 ± 12.8, p = 0.96	100.8 ± 16.2, p = 0.36
Low Risk	Chlorpromazine	100 nM	41	111.2 ± 10.4, p = 0.12	120.3 ± 22.5, p = 0.71	88.7 ± 14.7, p = 0.28	96.7 ± 18.6, p = 0.04
Low Risk	Chlorpromazine	300 nM	41	112.9 ± 13, p = 0.009	125.7 ± 25.6, p = 0.3	83.7 ± 14.3, p = 0.19	95 ± 21.9, p = 0.19
Low Risk	Chlorpromazine	1 µM	41	114.3 ± 14.8, p ≤ 0.001	130.2 ± 26.9, p = 0.02	79.8 ± 13.2, p = 0.02	89.2 ± 33.6, p = 0.14
Low Risk	Chlorpromazine	3 µM	41	112.5 ± 19.5, p ≤ 0.001	132.6 ± 33.8, p = 0.002	75.4 ± 14.4, p = 0.09	65.3 ± 31.5, p = 0.04
Low Risk	Ciprofloxacin	3 µM	65	105.6 ± 4.8, p = 0.48	106.3 ± 5.7, p ≤ 0.001	99.2 ± 8.2, p = 0.03	102.4 ± 20.3, p = 0.29
Low Risk	Ciprofloxacin	10 µM	65	107.9 ± 7.9, p = 0.67	109.5 ± 9.6, p = 0.12	98.2 ± 13.4, p = 0.29	100.6 ± 19.3, p = 0.43
Low Risk	Ciprofloxacin	30 µM	65	93.9 ± 9.8, p = 0.009	103.3 ± 10, p = 0.5	83.4 ± 11.8, p ≤ 0.001	104.2 ± 43.5, p = 0.3
Low Risk	Clarithromycin	1 µM	68	106.6 ± 6.6, p = 0.93	108.6 ± 6.7, p = 0.23	96.8 ± 8.9, p = 0.96	100.2 ± 13.4, p = 0.15
Low Risk	Clarithromycin	3 µM	68	110.4 ± 11.4, p = 0.82	119.3 ± 11, p = 0.6	86.3 ± 12.4, p = 0.99	95 ± 16.2, p = 0.1
Low Risk	Clarithromycin	10 µM	68	60.6 ± 26.2, p = 1	85 ± 31.6, p = 0.49	60.7 ± 59.3, p = 0.007	39.1 ± 41.2, p = 0.45
Low Risk	Dofetilide	1 nM	78	108.3 ± 4.7, p = 0.009	108 ± 4.4, p ≤ 0.001	100.7 ± 8.2, p = 0.37	98.4 ± 11.1, p = 0.4
Low Risk	Dofetilide	3 nM	78	124.3 ± 13.2, p ≤ 0.001	124.9 ± 12.5, p ≤ 0.001	99.5 ± 11.2, p = 0.28	95.3 ± 16.4, p = 0.58
Low Risk	Dofetilide	10 nM	78	157.3 ± 26.3, p ≤ 0.001	154.4 ± 21.8, p ≤ 0.001	109.1 ± 37.5, p = 0.37	87.2 ± 23.4, p = 0.26
Low Risk	Dofetilide	30 nM	78	203.5 ± 61.5, p ≤ 0.001	192.4 ± 41.7, p ≤ 0.001	116.8 ± 34.6, p = 0.26	76.5 ± 27.7, p = 0.05
Low Risk	Dofetilide	100 nM	78	236.9 ± 93.7, p ≤ 0.001	221.6 ± 65.8, p ≤ 0.001	134.9 ± 69.7, p = 0.49	62.8 ± 28.2, p = 0.08
Low Risk	E4031	10 nM	57	122.4 ± 17, p = 0.63	122.5 ± 14.9, p = 0.96	100.2 ± 10.1, p = 0.71	99.4 ± 22.2, p = 0.36
Low Risk	E4031	30 nM	57	158.6 ± 29, p ≤ 0.001	158 ± 26.8, p = 0.002	101.6 ± 16.1, p = 0.02	92.4 ± 30.2, p = 0.64
Low Risk	E4031	100 nM	57	212.2 ± 74.4, p ≤ 0.001	202.1 ± 54, p = 0.002	110.1 ± 34.2, p = 0.06	81.6 ± 40, p = 0.23
Low Risk	E4031	300 nM	57	239.4 ± 88.8, p ≤ 0.001	223.8 ± 64.9, p ≤ 0.001	116 ± 46, p ≤ 0.001	67.8 ± 46.9, p = 0.43
Low Risk	E4031	500 nM	57	255.9 ± 110.8, p ≤ 0.001	236.9 ± 84.4, p ≤ 0.001	130.4 ± 135.2, p = 0.01	62.3 ± 48.9, p = 0.11
Low Risk	HMR1556	300 nM	51	110.1 ± 6.2, p = 0.07	115.2 ± 13.8, p = 0.42	93.2 ± 12.3, p = 0.02	98.6 ± 23.3, p = 0.57
Low Risk	HMR1556	1 µM	51	110.9 ± 8.2, p = 0.003	119.3 ± 15.4, p = 0.98	88.2 ± 12.1, p = 0.02	94.3 ± 26.2, p = 0.57

Low Risk	HMR1556	3 µM	51	110.1 ± 10.9, p ≤ 0.001	122.3 ± 18.2, p = 0.11	82.9 ± 13.5, p ≤ 0.001	88.7 ± 25.5, p = 0.69
Low Risk	HMR1556	10 µM	51	87.4 ± 19.3, p = 0.001	104.3 ± 26.8, p = 0.15	72.5 ± 16.9, p ≤ 0.001	75.5 ± 43.2, p = 0.24
Low Risk	HMR1556 (after E4031)	E4031	54	137.2 ± 22.5, p = 0.59	133.7 ± 21.8, p = 0.46	107.2 ± 19.2, p = 0.64	94.6 ± 10.2, p = 0.08
Low Risk	HMR1556 (after E4031)	300 nM	54	169.4 ± 29.9, p = 0.004	166.2 ± 29.1, p = 0.03	105.5 ± 17.3, p = 0.06	86.7 ± 17.3, p = 0.31
Low Risk	HMR1556 (after E4031)	1 µM	54	172 ± 30.2, p = 0.02	173.8 ± 30.6, p = 0.05	99.6 ± 16.8, p = 0.64	80.4 ± 20, p = 0.28
Low Risk	HMR1556 (after E4031)	3 µM	54	165.1 ± 24.9, p = 0.53	173.8 ± 28.6, p = 0.65	92.1 ± 15.3, p = 0.27	74.9 ± 20.2, p = 0.17
Low Risk	HMR1556 (after E4031)	10 µM	54	108.1 ± 22.1, p = 0.13	130.3 ± 25.9, p = 0.51	69.7 ± 12.5, p = 0.11	69.8 ± 32.4, p = 0.74
Low Risk	Haloperidol	300 nM	68	140.8 ± 34.8, p ≤ 0.001	130.9 ± 28, p ≤ 0.001	115.5 ± 18.9, p = 0.05	93.5 ± 20.1, p = 0.17
Low Risk	Haloperidol	1 µM	68	219.5 ± 85, p ≤ 0.001	187.7 ± 55.9, p ≤ 0.001	135.9 ± 42.1, p = 0.05	70.3 ± 28.2, p = 0.93
Low Risk	Haloperidol	3 µM	68	227.8 ± 92.7, p ≤ 0.001	194.2 ± 56, p ≤ 0.001	134.9 ± 45.6, p = 0.47	49.6 ± 29.7, p = 0.81
Low Risk	Haloperidol	10 µM	63	169.8 ± 49.1, p = 0.02	154.9 ± 32.7, p = 0.003	125.5 ± 59.1, p = 0.95	41.1 ± 28.4, p = 0.04
Low Risk	Moxifloxacin	1 µM	51	105.8 ± 4.7, p = 0.23	108 ± 7.6, p = 0.88	97.1 ± 11.6, p = 0.7	96.3 ± 17.5, p = 0.82
Low Risk	Moxifloxacin	3 µM	51	108.3 ± 8.3, p = 0.21	113.9 ± 14.2, p = 0.55	92.2 ± 13, p = 0.24	93.7 ± 16.5, p = 0.15
Low Risk	Moxifloxacin	10 µM	51	112.3 ± 10.7, p = 0.07	120.7 ± 21.3, p = 0.73	89.1 ± 13.1, p = 0.08	91.3 ± 16.2, p = 0.23
Low Risk	Moxifloxacin	30 µM	51	120.5 ± 13.1, p = 0.12	130.6 ± 25.8, p = 0.75	88 ± 14.1, p = 0.03	88.1 ± 15.7, p = 0.9
Low Risk	Moxifloxacin	100 µM	50	282.2 ± 115.8, p = 0.008	256 ± 104.1, p = 0.08	134.4 ± 56.7, p ≤ 0.001	25.5 ± 33.6, p = 0.25
Low Risk	Nifedipine	30 nM	56	95.9 ± 9.1, p = 0.09	98.7 ± 9.6, p = 0.04	95.1 ± 12.9, p = 0.48	100.9 ± 15.4, p = 0.69
Low Risk	Nifedipine	100 nM	56	79.1 ± 17.5, p = 0.28	87.5 ± 16.8, p = 0.52	84.8 ± 27.5, p = 0.04	97.3 ± 20.8, p = 0.88
Low Risk	Nifedipine	300 nM	56	62.1 ± 19.8, p = 0.07	74.5 ± 19.8, p = 0.1	68.3 ± 12.7, p = 0.03	93 ± 23, p = 0.97
Low Risk	Nifedipine	1 µM	56	49.2 ± 20.6, p = 0.006	63.7 ± 21.3, p = 0.01	57.4 ± 11.8, p ≤ 0.001	89.5 ± 23, p = 0.83
Low Risk	Nifedipine	2 µM	56	34.6 ± 16.1, p ≤ 0.001	49.6 ± 18.5, p = 0.003	46.9 ± 11.3, p ≤ 0.001	85 ± 30.8, p = 0.85
Low Risk	Salbutamol	3 nM	49	103.9 ± 4.8, p ≤ 0.001	108.9 ± 7.9, p = 0.63	91.9 ± 10, p = 0.01	98.4 ± 13.8, p = 0.73
Low Risk	Salbutamol	10 nM	49	98.6 ± 9.1, p ≤ 0.001	110 ± 14.5, p = 0.64	81.7 ± 11.2, p ≤ 0.001	100.3 ± 15.7, p = 0.45
Low Risk	Salbutamol	30 nM	49	86.8 ± 10.9, p ≤ 0.001	105.5 ± 19, p = 0.62	69.9 ± 12.9, p ≤ 0.001	101.1 ± 20.8, p = 0.35
Low Risk	Salbutamol	100 nM	49	73.3 ± 10.2, p ≤ 0.001	94.6 ± 18, p = 0.83	62.2 ± 11.9, p ≤ 0.001	100.9 ± 28, p = 0.59

Low Risk	Salbutamol	300 nM	49	68.6 ± 7.5, p ≤ 0.001	90.8 ± 16.5, p = 0.4	63.1 ± 38.5, p ≤ 0.001	96.7 ± 25.4, p = 0.61
Low Risk	Tetrodotoxin	100 nM	57	106.3 ± 5.3, p = 0.13	106.5 ± 5.6, p = 0.57	100.2 ± 8.6, p = 0.63	102.2 ± 39.1, p = 0.04
Low Risk	Tetrodotoxin	300 nM	57	108.9 ± 7.4, p = 0.31	108.5 ± 11.4, p = 0.37	106.1 ± 49.5, p = 0.01	92.6 ± 35.3, p = 0.25
Low Risk	Tetrodotoxin	1 µM	57	109.9 ± 8.1, p = 0.58	108.7 ± 11.1, p = 0.96	104 ± 18, p = 0.1	72.4 ± 28.6, p = 0.69
Low Risk	Tetrodotoxin	10 µM	57	108.2 ± 9.4, p = 0.59	101.7 ± 10.9, p = 0.64	116.8 ± 30.8, p = 0.69	44.6 ± 21.8, p = 0.83
Low Risk	Tetrodotoxin	30 µM	57	100.6 ± 8.1, p = 0.01	84.7 ± 13.1, p = 0.58	167.6 ± 94.5, p = 0.39	10.9 ± 8.7, p = 0.009
High Risk	Chlorpromazine	30 nM	67	105.8 ± 4.3, p = 0.07	108.9 ± 7.8, p = 0.86	95.4 ± 12, p = 0.96	101.5 ± 16.1, p = 0.36
High Risk	Chlorpromazine	100 nM	67	106.8 ± 6.3, p = 0.12	114.9 ± 9.9, p = 0.71	87.9 ± 13.3, p = 0.28	101.7 ± 20.7, p = 0.04
High Risk	Chlorpromazine	300 nM	67	105.6 ± 8.6, p = 0.009	117.7 ± 12.6, p = 0.3	82.7 ± 18.6, p = 0.19	97.1 ± 24, p = 0.19
High Risk	Chlorpromazine	1 µM	67	101 ± 12.6, p ≤ 0.001	117.7 ± 16.3, p = 0.02	76 ± 21.6, p = 0.02	90.9 ± 24.2, p = 0.14
High Risk	Chlorpromazine	3 µM	67	93.7 ± 17.6, p ≤ 0.001	112.9 ± 23, p = 0.002	72.2 ± 27, p = 0.09	74.2 ± 29.1, p = 0.04
High Risk	Ciprofloxacin	3 µM	104	106.6 ± 5.5, p = 0.48	110.1 ± 9.5, p ≤ 0.001	95.3 ± 14.2, p = 0.03	98.2 ± 16.3, p = 0.29
High Risk	Ciprofloxacin	10 µM	104	108.8 ± 7.9, p = 0.67	113.5 ± 13.3, p = 0.12	94.3 ± 16.7, p = 0.29	98.1 ± 22.9, p = 0.43
High Risk	Ciprofloxacin	30 µM	104	90.2 ± 10.5, p = 0.009	107.1 ± 16.7, p = 0.5	73.2 ± 14.5, p ≤ 0.001	103.3 ± 27.7, p = 0.3
High Risk	Clarithromycin	1 µM	111	106.6 ± 6.3, p = 0.93	109.4 ± 7.3, p = 0.23	95.9 ± 12.8, p = 0.96	96.8 ± 9.3, p = 0.15
High Risk	Clarithromycin	3 µM	111	110.3 ± 11.4, p = 0.82	120.6 ± 15.8, p = 0.6	86.3 ± 19.4, p = 0.99	90.3 ± 14.6, p = 0.1
High Risk	Clarithromycin	10 µM	111	74.8 ± 79.5, p = 1	93.2 ± 78.8, p = 0.49	88.3 ± 136.7, p = 0.007	41.8 ± 36.1, p = 0.45
High Risk	Dofetilide	1 nM	121	111.1 ± 7, p = 0.009	111.9 ± 9.9, p ≤ 0.001	100.2 ± 16.3, p = 0.37	99.7 ± 8.8, p = 0.4
High Risk	Dofetilide	3 nM	121	133.2 ± 19.3, p ≤ 0.001	135.4 ± 17.5, p ≤ 0.001	98.1 ± 18.3, p = 0.28	95.5 ± 15.6, p = 0.58
High Risk	Dofetilide	10 nM	121	202.4 ± 55.8, p ≤ 0.001	192.1 ± 45.3, p ≤ 0.001	128.2 ± 193.3, p = 0.37	84.3 ± 21.5, p = 0.26
High Risk	Dofetilide	30 nM	121	298.9 ± 114.8, p ≤ 0.001	269.6 ± 101.7, p ≤ 0.001	132.4 ± 80.5, p = 0.26	68.5 ± 30.6, p = 0.05
High Risk	Dofetilide	100 nM	121	374.1 ± 153.7, p ≤ 0.001	317.5 ± 128.5, p ≤ 0.001	156.4 ± 142.2, p = 0.49	54.6 ± 32.4, p = 0.08
High Risk	E4031	10 nM	77	128.3 ± 35, p = 0.63	126.8 ± 28.4, p = 0.96	101.8 ± 13.8, p = 0.71	97 ± 12.6, p = 0.36
High Risk	E4031	30 nM	77	204 ± 80.4, p ≤ 0.001	192.5 ± 78.7, p = 0.002	114.2 ± 28.1, p = 0.02	87.5 ± 16.3, p = 0.64
High Risk	E4031	100 nM	77	290.4 ± 186.5, p ≤ 0.001	253.8 ± 129.3, p = 0.002	124.4 ± 46.6, p = 0.06	71.1 ± 27.4, p = 0.23

High Risk	E4031	300 nM	77	374.1 ± 215.6, p ≤ 0.001	314 ± 166.4, p ≤ 0.001	148 ± 73, p ≤ 0.001	59.4 ± 25, p = 0.43
High Risk	E4031	500 nM	73	413.6 ± 245.1, p ≤ 0.001	350 ± 183.2, p ≤ 0.001	162.6 ± 166.1, p = 0.01	48.3 ± 29.8, p = 0.11
High Risk	HMR1556	300 nM	75	107.8 ± 7.6, p = 0.07	116.9 ± 17.5, p = 0.42	88.5 ± 18.6, p = 0.02	96.9 ± 11.8, p = 0.57
High Risk	HMR1556	1 µM	75	106.2 ± 8.1, p = 0.003	120.1 ± 21.5, p = 0.98	82.1 ± 17.7, p = 0.02	92.7 ± 15.7, p = 0.57
High Risk	HMR1556	3 µM	75	99.4 ± 9.6, p ≤ 0.001	120 ± 25.1, p = 0.11	73.2 ± 18.9, p ≤ 0.001	88.1 ± 16.2, p = 0.69
High Risk	HMR1556	10 µM	72	74 ± 18.7, p = 0.001	97.5 ± 26.2, p = 0.15	63.8 ± 46.5, p ≤ 0.001	75.9 ± 21.5, p = 0.24
High Risk	HMR1556 (after E4031)	E4031	73	151.1 ± 61.1, p = 0.59	140.2 ± 41, p = 0.46	114.5 ± 31.2, p = 0.64	96.7 ± 10.5, p = 0.08
High Risk	HMR1556 (after E4031)	300 nM	73	196.5 ± 53.4, p = 0.004	183.2 ± 42.6, p = 0.03	116.4 ± 30.1, p = 0.06	90.8 ± 17.6, p = 0.31
High Risk	HMR1556 (after E4031)	1 µM	73	190.6 ± 43.8, p = 0.02	188.4 ± 40.6, p = 0.05	105.2 ± 28.7, p = 0.64	85.2 ± 18.4, p = 0.28
High Risk	HMR1556 (after E4031)	3 µM	73	169.5 ± 34.4, p = 0.53	180.2 ± 42.7, p = 0.65	92.8 ± 26.7, p = 0.27	79.6 ± 20.8, p = 0.17
High Risk	HMR1556 (after E4031)	10 µM	73	102.6 ± 29.4, p = 0.13	128.1 ± 35.9, p = 0.51	66.3 ± 18.4, p = 0.11	67.3 ± 22.1, p = 0.74
High Risk	Haloperidol	300 nM	118	203.4 ± 143.1, p ≤ 0.001	180.1 ± 109, p ≤ 0.001	123.5 ± 33.3, p = 0.05	91.8 ± 16.1, p = 0.17
High Risk	Haloperidol	1 µM	118	306.9 ± 180.3, p ≤ 0.001	253.1 ± 134, p ≤ 0.001	149.2 ± 65.7, p = 0.05	71.9 ± 26.1, p = 0.93
High Risk	Haloperidol	3 µM	118	287.2 ± 148.6, p ≤ 0.001	251 ± 125.2, p ≤ 0.001	139.8 ± 59.9, p = 0.47	47.4 ± 28.1, p = 0.81
High Risk	Haloperidol	10 µM	118	236.9 ± 169.1, p = 0.02	212.9 ± 126, p = 0.003	144.6 ± 128.6, p = 0.95	32.4 ± 26.3, p = 0.04
High Risk	Moxifloxacin	1 µM	68	104.8 ± 4.9, p = 0.23	108.2 ± 9.2, p = 0.88	96 ± 18.6, p = 0.7	99 ± 8.9, p = 0.82
High Risk	Moxifloxacin	3 µM	68	106.5 ± 7.8, p = 0.21	114.8 ± 13.8, p = 0.55	88.7 ± 16.6, p = 0.24	98.1 ± 9, p = 0.15
High Risk	Moxifloxacin	10 µM	68	108.8 ± 11.1, p = 0.07	123.4 ± 26.5, p = 0.73	82.7 ± 19.3, p = 0.08	93.4 ± 10.4, p = 0.23
High Risk	Moxifloxacin	30 µM	68	116.6 ± 18, p = 0.12	134.3 ± 32.8, p = 0.75	80.5 ± 20, p = 0.03	88.1 ± 21.5, p = 0.9
High Risk	Moxifloxacin	100 µM	68	480.7 ± 277.4, p = 0.008	357.1 ± 199.6, p = 0.08	331.4 ± 452.4, p ≤ 0.001	21.8 ± 14.9, p = 0.25
High Risk	Nifedipine	30 nM	80	91.5 ± 17.1, p = 0.09	94.1 ± 13.8, p = 0.04	94.8 ± 23.7, p = 0.48	99.5 ± 16.9, p = 0.69
High Risk	Nifedipine	100 nM	80	75.2 ± 17.3, p = 0.28	85.4 ± 17.6, p = 0.52	78.5 ± 22.5, p = 0.04	97 ± 20.7, p = 0.88
High Risk	Nifedipine	300 nM	80	55.5 ± 19.4, p = 0.07	68.7 ± 20.5, p = 0.1	65.9 ± 30.9, p = 0.03	92.3 ± 22.6, p = 0.97
High Risk	Nifedipine	1 µM	80	39.4 ± 17.9, p = 0.006	54.1 ± 19.1, p = 0.01	52 ± 22.9, p ≤ 0.001	89 ± 24.8, p = 0.83
High Risk	Nifedipine	2 µM	76	26.2 ± 13.7, p ≤ 0.001	41.1 ± 19.2, p = 0.003	39.2 ± 8, p ≤ 0.001	86 ± 24.9, p = 0.85

High Risk	Salbutamol	3 nM	72	99.5 ± 8.9, p ≤ 0.001	109.2 ± 14.7, p = 0.63	85.4 ± 14.9, p = 0.01	100.7 ± 14.3, p = 0.73
High Risk	Salbutamol	10 nM	72	91.6 ± 11.2, p ≤ 0.001	110.9 ± 21.5, p = 0.64	71.9 ± 16.5, p ≤ 0.001	103.1 ± 17.4, p = 0.45
High Risk	Salbutamol	30 nM	72	76.2 ± 16, p ≤ 0.001	102.7 ± 24.4, p = 0.62	57.6 ± 14.7, p ≤ 0.001	98.2 ± 20.5, p = 0.35
High Risk	Salbutamol	100 nM	72	62.9 ± 15.7, p ≤ 0.001	92.6 ± 24.3, p = 0.83	48.3 ± 13.3, p ≤ 0.001	97.6 ± 26, p = 0.59
High Risk	Salbutamol	300 nM	72	56.5 ± 13.5, p ≤ 0.001	87.8 ± 20.9, p = 0.4	43.5 ± 12, p ≤ 0.001	94.4 ± 26.1, p = 0.61
High Risk	Tetrodotoxin	100 nM	72	105 ± 4, p = 0.13	106.4 ± 7.8, p = 0.57	98.6 ± 13.9, p = 0.63	95.5 ± 18.3, p = 0.04
High Risk	Tetrodotoxin	300 nM	72	107.5 ± 7, p = 0.31	111.1 ± 10.9, p = 0.37	95.3 ± 16.4, p = 0.01	86.4 ± 19.8, p = 0.25
High Risk	Tetrodotoxin	1 µM	72	108.1 ± 8.5, p = 0.58	109.6 ± 13.2, p = 0.96	101.6 ± 28.8, p = 0.1	70.6 ± 20.3, p = 0.69
High Risk	Tetrodotoxin	10 µM	72	108.7 ± 9.3, p = 0.59	103.6 ± 18.6, p = 0.64	127.9 ± 92.6, p = 0.69	44.5 ± 19, p = 0.83
High Risk	Tetrodotoxin	30 µM	69	108.7 ± 12.2, p = 0.01	92 ± 27.1, p = 0.58	160.4 ± 60.5, p = 0.39	19.2 ± 13.5, p = 0.009

Changes of field potential duration (FPD) corrected field potential duration (cFPD), RR interval (RR), and peak-to-peak amplitude (PtPA) are presented as percentage of the Vehicle treatment response. Values are presented as mean ± standard deviation. N indicates the number of hiPSC-CMs monolayers included in the analysis. Wilcoxon rank-sum test p-values are provided.

Supplemental Table 8. Differential drug-concentration responses to dofetilide, E4031, haloperidol, HMR1556 following E4031 pre-treatment, and nifedipine in *KCNQ1* p.A341V carrying hiPSC-CM lines.

Cell Line	Drug	Concentration	N	Normalized cFPD (% of Vehicle)
KCNQ1-p.A341V (SA6.27 line)	Dofetilide	1 nM	16	113.7 ± 11.4, p = 0.45
KCNQ1-p.A341V (SA6.27 line)	Dofetilide	3 nM	16	133.6 ± 14.6, p = 0.44
KCNQ1-p.A341V (SA6.27 line)	Dofetilide	10 nM	16	193.7 ± 52.9, p = 0.33
KCNQ1-p.A341V (SA6.27 line)	Dofetilide	30 nM	16	317.9 ± 91.3, p = 0.005
KCNQ1-p.A341V (SA6.27 line)	Dofetilide	100 nM	16	376.5 ± 93.8, p = 0.005
KCNQ1-p.A341V (SA6.27 line)	E4031	10 nM	9	133.1 ± 30.6, p = 0.63
KCNQ1-p.A341V (SA6.27 line)	E4031	30 nM	9	249.7 ± 38.2, p ≤ 0.001
KCNQ1-p.A341V (SA6.27 line)	E4031	100 nM	9	286.1 ± 21.1, p ≤ 0.001
KCNQ1-p.A341V (SA6.27 line)	E4031	300 nM	9	354.7 ± 30.4, p ≤ 0.001
KCNQ1-p.A341V (SA6.27 line)	E4031	500 nM	9	359 ± 31.7, p ≤ 0.001
KCNQ1-p.A341V (SA6.27 line)	HMR1556 (after E4031)	E4031 20 nM	9	157.8 ± 51.1, p = 0.08
KCNQ1-p.A341V (SA6.27 line)	HMR1556 (after E4031)	300 nM	9	222.8 ± 22.7, p ≤ 0.001
KCNQ1-p.A341V (SA6.27 line)	HMR1556 (after E4031)	1 µM	9	225.1 ± 22.4, p ≤ 0.001
KCNQ1-p.A341V (SA6.27 line)	HMR1556 (after E4031)	3 µM	9	210.4 ± 25.9, p = 0.005
KCNQ1-p.A341V (SA6.27 line)	HMR1556 (after E4031)	10 µM	9	124.6 ± 21.3, p = 0.69
KCNQ1-p.A341V (SA6.27 line)	Haloperidol	300 nM	16	155.6 ± 48.9, p = 0.08
KCNQ1-p.A341V (SA6.27 line)	Haloperidol	1 µM	16	261 ± 62.7, p ≤ 0.001
KCNQ1-p.A341V (SA6.27 line)	Haloperidol	3 µM	16	301.3 ± 62, p ≤ 0.001
KCNQ1-p.A341V (SA6.27 line)	Haloperidol	10 µM	16	226.6 ± 51.3, p ≤ 0.001
KCNQ1-p.A341V (SA6.27 line)	Nifedipine	30 nM	9	101.5 ± 7.5, p = 0.11
KCNQ1-p.A341V (SA6.27 line)	Nifedipine	100 nM	9	91.9 ± 10.8, p = 0.49
KCNQ1-p.A341V (SA6.27 line)	Nifedipine	300 nM	9	72.6 ± 16.3, p = 0.44
KCNQ1-p.A341V (SA6.27 line)	Nifedipine	1 µM	9	65.5 ± 13.4, p = 0.12
KCNQ1-p.A341V (SA6.27 line)	Nifedipine	2 µM	9	43.2 ± 12.3, p = 0.24
KCNQ1-p.A341V (SA15.14 line)	Dofetilide	1 nM	18	110.8 ± 6.8, p = 0.45
KCNQ1-p.A341V (SA15.14 line)	Dofetilide	3 nM	18	131.3 ± 14.6, p = 0.44
KCNQ1-p.A341V (SA15.14 line)	Dofetilide	10 nM	18	187.2 ± 30.4, p = 0.33
KCNQ1-p.A341V (SA15.14 line)	Dofetilide	30 nM	18	245.8 ± 60.3, p = 0.005
KCNQ1-p.A341V (SA15.14 line)	Dofetilide	100 nM	18	299.5 ± 71.9, p = 0.005
KCNQ1-p.A341V (SA15.14 line)	E4031	10 nM	9	120.1 ± 16.7, p = 0.63
KCNQ1-p.A341V (SA15.14 line)	E4031	30 nM	9	180.2 ± 34.3, p ≤ 0.001
KCNQ1-p.A341V (SA15.14 line)	E4031	100 nM	9	255.7 ± 42.2, p ≤ 0.001
KCNQ1-p.A341V (SA15.14 line)	E4031	300 nM	9	336.6 ± 73.3, p ≤ 0.001

KCNQ1-p.A341V (SA15.14 line)	E4031	500 nM	9	390.9 ± 95, p ≤ 0.001
KCNQ1-p.A341V (SA15.14 line)	HMR1556 (after E4031)	E4031 20 nM	9	119.9 ± 13.1, p = 0.08
KCNQ1-p.A341V (SA15.14 line)	HMR1556 (after E4031)	300 nM	9	177.1 ± 24.2, p ≤ 0.001
KCNQ1-p.A341V (SA15.14 line)	HMR1556 (after E4031)	1 µM	9	194.7 ± 10.2, p ≤ 0.001
KCNQ1-p.A341V (SA15.14 line)	HMR1556 (after E4031)	3 µM	9	190.5 ± 20.6, p = 0.005
KCNQ1-p.A341V (SA15.14 line)	HMR1556 (after E4031)	10 µM	9	145.7 ± 37.5, p = 0.69
KCNQ1-p.A341V (SA15.14 line)	Haloperidol	300 nM	18	152.8 ± 42.4, p = 0.08
KCNQ1-p.A341V (SA15.14 line)	Haloperidol	1 µM	18	232.7 ± 41.9, p ≤ 0.001
KCNQ1-p.A341V (SA15.14 line)	Haloperidol	3 µM	18	257.4 ± 77.2, p ≤ 0.001
KCNQ1-p.A341V (SA15.14 line)	Haloperidol	10 µM	18	255 ± 92.8, p ≤ 0.001
KCNQ1-p.A341V (SA15.14 line)	Nifedipine	30 nM	9	79.9 ± 25, p = 0.11
KCNQ1-p.A341V (SA15.14 line)	Nifedipine	100 nM	9	76.3 ± 23.3, p = 0.49
KCNQ1-p.A341V (SA15.14 line)	Nifedipine	300 nM	9	58 ± 21, p = 0.44
KCNQ1-p.A341V (SA15.14 line)	Nifedipine	1 µM	9	47.3 ± 16.9, p = 0.12
KCNQ1-p.A341V (SA15.14 line)	Nifedipine	2 µM	9	32.4 ± 15.1, p = 0.24
KCNQ1-p.A341V, NOS1AP hetero (SA14.19 line)	Dofetilide	1 nM	17	115.8 ± 9.2, p = 0.45
KCNQ1-p.A341V, NOS1AP hetero (SA14.19 line)	Dofetilide	3 nM	17	139.3 ± 16.9, p = 0.44
KCNQ1-p.A341V, NOS1AP hetero (SA14.19 line)	Dofetilide	10 nM	17	184.9 ± 38.2, p = 0.33
KCNQ1-p.A341V, NOS1AP hetero (SA14.19 line)	Dofetilide	30 nM	17	245.8 ± 40.3, p = 0.005
KCNQ1-p.A341V, NOS1AP hetero (SA14.19 line)	Dofetilide	100 nM	17	259.3 ± 47.1, p = 0.005
KCNQ1-p.A341V, NOS1AP hetero (SA14.19 line)	E4031	10 nM	9	117.7 ± 12, p = 0.63
KCNQ1-p.A341V, NOS1AP hetero (SA14.19 line)	E4031	30 nM	9	152.3 ± 23.7, p ≤ 0.001
KCNQ1-p.A341V, NOS1AP hetero (SA14.19 line)	E4031	100 nM	9	196.4 ± 26.5, p ≤ 0.001
KCNQ1-p.A341V, NOS1AP hetero (SA14.19 line)	E4031	300 nM	9	253.4 ± 78.9, p ≤ 0.001
KCNQ1-p.A341V, NOS1AP hetero (SA14.19 line)	E4031	500 nM	9	281.8 ± 70.6, p ≤ 0.001
KCNQ1-p.A341V, NOS1AP hetero (SA14.19 line)	HMR1556 (after E4031)	E4031 20 nM	9	125.6 ± 19.4, p = 0.08
KCNQ1-p.A341V, NOS1AP hetero (SA14.19 line)	HMR1556 (after E4031)	300 nM	9	163.5 ± 20.3, p ≤ 0.001
KCNQ1-p.A341V, NOS1AP hetero (SA14.19 line)	HMR1556 (after E4031)	1 µM	9	171.2 ± 20.8, p ≤ 0.001
KCNQ1-p.A341V, NOS1AP hetero (SA14.19 line)	HMR1556 (after E4031)	3 µM	9	168 ± 16.5, p = 0.005
KCNQ1-p.A341V, NOS1AP hetero (SA14.19 line)	HMR1556 (after E4031)	10 µM	9	129.5 ± 24.2, p = 0.69
KCNQ1-p.A341V, NOS1AP hetero (SA14.19 line)	Haloperidol	300 nM	16	149 ± 38.1, p = 0.08
KCNQ1-p.A341V, NOS1AP hetero (SA14.19 line)	Haloperidol	1 µM	16	177.4 ± 37, p ≤ 0.001
KCNQ1-p.A341V, NOS1AP hetero (SA14.19 line)	Haloperidol	3 µM	16	189.3 ± 42.7, p ≤ 0.001
KCNQ1-p.A341V, NOS1AP hetero (SA14.19 line)	Haloperidol	10 µM	16	162.1 ± 42.5, p ≤ 0.001
KCNQ1-p.A341V, NOS1AP hetero (SA14.19 line)	Nifedipine	30 nM	9	96.8 ± 11.3, p = 0.11
KCNQ1-p.A341V, NOS1AP hetero (SA14.19 line)	Nifedipine	100 nM	9	84.6 ± 18.3, p = 0.49
KCNQ1-p.A341V, NOS1AP hetero (SA14.19 line)	Nifedipine	300 nM	9	70.9 ± 28.5, p = 0.44
KCNQ1-p.A341V, NOS1AP hetero (SA14.19 line)	Nifedipine	1 µM	9	62.3 ± 29.5, p = 0.12
KCNQ1-p.A341V, NOS1AP hetero (SA14.19 line)	Nifedipine	2 µM	9	56.8 ± 32.2, p = 0.24

KCNQ1-p.A341V, NOS1AP homo (SA13.5 line)	Dofetilide	1 nM	14	114.4 ± 15.1, p = 0.45
KCNQ1-p.A341V, NOS1AP homo (SA13.5 line)	Dofetilide	3 nM	14	136.1 ± 11.6, p = 0.44
KCNQ1-p.A341V, NOS1AP homo (SA13.5 line)	Dofetilide	10 nM	14	212.4 ± 41.4, p = 0.33
KCNQ1-p.A341V, NOS1AP homo (SA13.5 line)	Dofetilide	30 nM	14	368.7 ± 159.5, p = 0.005
KCNQ1-p.A341V, NOS1AP homo (SA13.5 line)	Dofetilide	100 nM	14	423.6 ± 234.8, p = 0.005
KCNQ1-p.A341V, NOS1AP homo (SA13.5 line)	E4031	10 nM	10	144.2 ± 52, p = 0.63
KCNQ1-p.A341V, NOS1AP homo (SA13.5 line)	E4031	30 nM	10	287.7 ± 155.6, p ≤ 0.001
KCNQ1-p.A341V, NOS1AP homo (SA13.5 line)	E4031	100 nM	10	485.8 ± 212.9, p ≤ 0.001
KCNQ1-p.A341V, NOS1AP homo (SA13.5 line)	E4031	300 nM	10	608.4 ± 238.2, p ≤ 0.001
KCNQ1-p.A341V, NOS1AP homo (SA13.5 line)	E4031	500 nM	10	657.9 ± 230, p ≤ 0.001
KCNQ1-p.A341V, NOS1AP homo (SA13.5 line)	HMR1556 (after E4031)	E4031 20 nM	10	161.4 ± 49.5, p = 0.08
KCNQ1-p.A341V, NOS1AP homo (SA13.5 line)	HMR1556 (after E4031)	300 nM	10	229.1 ± 58.7, p ≤ 0.001
KCNQ1-p.A341V, NOS1AP homo (SA13.5 line)	HMR1556 (after E4031)	1 µM	10	237.1 ± 54.2, p ≤ 0.001
KCNQ1-p.A341V, NOS1AP homo (SA13.5 line)	HMR1556 (after E4031)	3 µM	10	237.7 ± 62.8, p = 0.005
KCNQ1-p.A341V, NOS1AP homo (SA13.5 line)	HMR1556 (after E4031)	10 µM	10	154.8 ± 64.7, p = 0.69
KCNQ1-p.A341V, NOS1AP homo (SA13.5 line)	Haloperidol	300 nM	14	326.3 ± 233.9, p = 0.08
KCNQ1-p.A341V, NOS1AP homo (SA13.5 line)	Haloperidol	1 µM	14	504.7 ± 226.3, p ≤ 0.001
KCNQ1-p.A341V, NOS1AP homo (SA13.5 line)	Haloperidol	3 µM	14	472.7 ± 205.8, p ≤ 0.001
KCNQ1-p.A341V, NOS1AP homo (SA13.5 line)	Haloperidol	10 µM	14	417.9 ± 213, p ≤ 0.001
KCNQ1-p.A341V, NOS1AP homo (SA13.5 line)	Nifedipine	30 nM	10	97.9 ± 14.5, p = 0.11
KCNQ1-p.A341V, NOS1AP homo (SA13.5 line)	Nifedipine	100 nM	10	94.4 ± 25.8, p = 0.49
KCNQ1-p.A341V, NOS1AP homo (SA13.5 line)	Nifedipine	300 nM	10	79.6 ± 29.4, p = 0.44
KCNQ1-p.A341V, NOS1AP homo (SA13.5 line)	Nifedipine	1 µM	10	45.7 ± 11.5, p = 0.12
KCNQ1-p.A341V, NOS1AP homo (SA13.5 line)	Nifedipine	2 µM	10	39.6 ± 23.5, p = 0.24

Changes of corrected field potential duration (cFPD) are presented as percentage of the Vehicle treatment response. Values are presented as mean ± standard deviation. N indicates the number of hiPSC-CMs monolayers included in the analysis. Kruskal-Wallis test for multiple group comparison p-values are provided.

Supplemental Table 9. Differential drug-concentration responses to dofetilide, E4031, and haloperidol in High Risk and Low Risk *KCNQ1* hiPSC-CMs.

Variant Risk Level	Drug	Concentration	N	Normalized FPD (% of Vehicle)	Normalized cFPD (% of Vehicle)	Normalized RR (% of Vehicle)	Normalized PtPA (% of Vehicle)
Low Risk	Dofetilide	1 nM	17	108.8 ± 2.6, p = 0.08	109.2 ± 2.8, p = 0.1	99.3 ± 4.2, p = 0.7	100.4 ± 16.8, p = 0.87
Low Risk	Dofetilide	3 nM	17	125.9 ± 7.2, p = 0.09	126.2 ± 7.3, p = 0.02	99.7 ± 5.4, p = 0.43	102.1 ± 16.6, p = 0.06
Low Risk	Dofetilide	10 nM	17	172.1 ± 23.8, p = 0.05	166.1 ± 21.7, p = 0.01	107.4 ± 7.4, p = 0.71	95.3 ± 22.4, p = 0.07
Low Risk	Dofetilide	30 nM	17	233.3 ± 71.9, p = 0.005	206.2 ± 40, p ≤ 0.001	129.8 ± 37.6, p = 0.85	84.2 ± 23.5, p = 0.25
Low Risk	Dofetilide	100 nM	17	251.5 ± 159.5, p ≤ 0.001	218.7 ± 88.9, p ≤ 0.001	182.7 ± 108.9, p = 0.33	59.9 ± 33.2, p = 0.66
High Risk	Dofetilide	1 nM	101	111.3 ± 7, p = 0.08	112.3 ± 10.3, p = 0.1	100.1 ± 17, p = 0.7	99 ± 8.5, p = 0.87
High Risk	Dofetilide	3 nM	101	131.5 ± 15.4, p = 0.09	134.8 ± 16.7, p = 0.02	96.9 ± 17.9, p = 0.43	95.5 ± 13.9, p = 0.06
High Risk	Dofetilide	10 nM	101	197.5 ± 51.4, p = 0.05	191 ± 44.6, p = 0.01	127.5 ± 211, p = 0.71	87.2 ± 18.6, p = 0.07
High Risk	Dofetilide	30 nM	101	303.8 ± 110.5, p = 0.005	274.4 ± 100, p ≤ 0.001	137.4 ± 84.4, p = 0.85	74.6 ± 25.8, p = 0.25
High Risk	Dofetilide	100 nM	101	377.8 ± 154.6, p ≤ 0.001	319.2 ± 129.6, p ≤ 0.001	165.4 ± 150.2, p = 0.33	58 ± 31, p = 0.66
Low Risk	E4031	10 nM	16	121.1 ± 18, p = 0.69	119.6 ± 15.1, p = 0.44	102.4 ± 8, p = 0.62	98.7 ± 16.3, p = 0.3
Low Risk	E4031	30 nM	16	139.9 ± 22.9, p ≤ 0.001	139 ± 18, p ≤ 0.001	100.7 ± 8.6, p = 0.19	101.8 ± 18.2, p = 0.002
Low Risk	E4031	100 nM	16	172.4 ± 32.9, p ≤ 0.001	168.7 ± 23.9, p ≤ 0.001	103.5 ± 11.3, p = 0.13	92.9 ± 16.1, p = 0.004
Low Risk	E4031	300 nM	16	193.6 ± 30.9, p ≤ 0.001	185.9 ± 19.1, p ≤ 0.001	108.9 ± 23.9, p = 0.002	74.7 ± 33.3, p = 0.07
Low Risk	E4031	500 nM	16	199.4 ± 24.6, p ≤ 0.001	184.1 ± 37.4, p ≤ 0.001	167.4 ± 241, p = 0.08	66.2 ± 34.1, p = 0.15
High Risk	E4031	10 nM	64	129.6 ± 35.8, p = 0.69	128.6 ± 29, p = 0.44	101.1 ± 14, p = 0.62	97.6 ± 13.3, p = 0.3
High Risk	E4031	30 nM	64	199.4 ± 80.1, p ≤ 0.001	192.6 ± 82.8, p ≤ 0.001	109.8 ± 25.5, p = 0.19	88.7 ± 15.1, p = 0.002
High Risk	E4031	100 nM	64	291.1 ± 200.1, p ≤ 0.001	256.5 ± 138.6, p ≤ 0.001	122.8 ± 46.5, p = 0.13	72.6 ± 26.4, p = 0.004
High Risk	E4031	300 nM	64	394.6 ± 225.9, p ≤ 0.001	333 ± 173, p ≤ 0.001	147 ± 74, p = 0.002	61.9 ± 22.7, p = 0.07
High Risk	E4031	500 nM	60	447.1 ± 250.4, p ≤ 0.001	377.2 ± 184.9, p ≤ 0.001	171 ± 180.2, p = 0.08	52.9 ± 27.4, p = 0.15
Low Risk	Haloperidol	300 nM	16	134.6 ± 30.9, p = 0.009	124 ± 24.7, p = 0.002	117.7 ± 19.9, p = 0.56	96.5 ± 25.1, p = 0.84
Low Risk	Haloperidol	1 µM	16	220.2 ± 87.5, p = 0.006	183.4 ± 50.7, p = 0.002	142.1 ± 52.2, p = 0.33	71.1 ± 24.6, p = 0.39
Low Risk	Haloperidol	3 µM	16	221.6 ± 75.8, p = 0.008	180.4 ± 39.1, p = 0.001	151.1 ± 61.8, p = 0.69	42.6 ± 30.4, p = 0.2
Low Risk	Haloperidol	10 µM	16	192.5 ± 54.1, p = 0.39	154.4 ± 25.6, p = 0.007	169.6 ± 87, p = 0.04	31.7 ± 26.2, p = 0.7

High Risk	Haloperidol	300 nM	99	203.3 ± 151.2, p = 0.009	182.5 ± 116.6, p = 0.002	120.3 ± 31.4, p = 0.56	92.8 ± 14.5, p = 0.84
High Risk	Haloperidol	1 µM	99	312 ± 192.3, p = 0.006	258.7 ± 142.7, p = 0.002	149 ± 68.3, p = 0.33	75.3 ± 23.9, p = 0.39
High Risk	Haloperidol	3 µM	99	301.9 ± 154.3, p = 0.008	264 ± 129.6, p = 0.001	143.8 ± 62.2, p = 0.69	50.5 ± 27.1, p = 0.2
High Risk	Haloperidol	10 µM	99	252.6 ± 175.7, p = 0.39	226 ± 129.7, p = 0.007	153.4 ± 138.4, p = 0.04	34.5 ± 26.5, p = 0.7

Changes of field potential duration (FPD) corrected field potential duration (cFPD), RR interval (RR), and peak-to-peak amplitude (PtPA) are presented as percentage of the Vehicle treatment response. Values are presented as mean ± standard deviation. N indicates the number of hiPSC-CMs monolayers included in the analysis. Wilcoxon rank-sum test p-values are provided.

Supplemental Table 10. Differential drug-concentration responses to dofetilide, E4031, and haloperidol in High Risk and Low Risk *KCNH2* hiPSC-CMs.

Variant Risk Level	Drug	Concentration	N	Normalized FPD (% of Vehicle)	Normalized cFPD (% of Vehicle)	Normalized RR (% of Vehicle)	Normalized PtPA (% of Vehicle)
Low Risk	Dofetilide	1 nM	30	110.1 ± 3.8, p = 0.59	108.1 ± 5, p = 0.4	104.3 ± 8.2, p = 0.009	97.8 ± 10.7, p = 0.09
Low Risk	Dofetilide	3 nM	30	125.3 ± 8.2, p = 0.18	123.1 ± 7.8, p = 0.004	104.2 ± 11, p = 0.59	94.9 ± 17.8, p = 0.25
Low Risk	Dofetilide	10 nM	30	157.5 ± 20.8, p ≤ 0.001	150.5 ± 17.9, p ≤ 0.001	111.3 ± 23.3, p = 0.27	84.5 ± 27.9, p = 0.08
Low Risk	Dofetilide	30 nM	30	202.4 ± 46.1, p = 0.71	189.6 ± 41.6, p = 0.71	114.9 ± 18.3, p = 0.09	77.1 ± 26.6, p ≤ 0.001
Low Risk	Dofetilide	100 nM	30	236.3 ± 77.2, p = 0.09	216.6 ± 64.7, p = 0.1	126.2 ± 41.8, p = 0.07	67.6 ± 23.6, p = 0.002
High Risk	Dofetilide	1 nM	20	110.4 ± 6.7, p = 0.59	110.2 ± 7.3, p = 0.4	101.1 ± 12.1, p = 0.009	103.2 ± 9.6, p = 0.09
High Risk	Dofetilide	3 nM	20	141.7 ± 31.9, p = 0.18	138.3 ± 21.4, p = 0.004	104.3 ± 19.3, p = 0.59	95.7 ± 22.8, p = 0.25
High Risk	Dofetilide	10 nM	20	231.9 ± 71.8, p ≤ 0.001	198.9 ± 50.1, p ≤ 0.001	131.7 ± 38.3, p = 0.27	69.4 ± 28.5, p = 0.08
High Risk	Dofetilide	30 nM	20	255.1 ± 146.9, p = 0.71	226.1 ± 111.5, p = 0.71	107 ± 51.3, p = 0.09	37.2 ± 34.1, p ≤ 0.001
High Risk	Dofetilide	100 nM	20	331.7 ± 146, p = 0.09	297.4 ± 121.2, p = 0.1	100.2 ± 47.6, p = 0.07	33.5 ± 33.6, p = 0.002
Low Risk	E4031	10 nM	18	123.1 ± 19.5, p = 0.39	124.4 ± 16.5, p = 0.31	98.3 ± 13, p = 0.17	109.9 ± 28.7, p = 0.02
Low Risk	E4031	30 nM	18	172.7 ± 25.7, p = 0.13	171.2 ± 26.1, p = 0.39	103.1 ± 17.2, p = 0.004	98.9 ± 45.6, p = 0.42
Low Risk	E4031	100 nM	18	228.4 ± 54.9, p = 0.03	222.2 ± 58.6, p = 0.08	107.5 ± 17.1, p = 0.15	93.4 ± 60.6, p = 0.17
Low Risk	E4031	300 nM	18	274.1 ± 75.2, p = 0.45	259.9 ± 72, p = 0.37	112.7 ± 18.5, p = 0.05	86.5 ± 68.6, p = 0.05
Low Risk	E4031	500 nM	18	283.1 ± 83.6, p = 0.78	269 ± 83.5, p = 0.13	113.1 ± 21.3, p = 0.7	82.9 ± 73.1, p = 0.002
High Risk	E4031	10 nM	13	122 ± 31, p = 0.39	118 ± 24.2, p = 0.31	105.5 ± 12.6, p = 0.17	94.3 ± 8.1, p = 0.02
High Risk	E4031	30 nM	13	226.5 ± 80.8, p = 0.13	192.2 ± 56.2, p = 0.39	135.9 ± 31, p = 0.004	81.6 ± 20.9, p = 0.42
High Risk	E4031	100 nM	13	286.7 ± 70.5, p = 0.03	238.6 ± 49.5, p = 0.08	132.2 ± 47.8, p = 0.15	63.5 ± 31.9, p = 0.17
High Risk	E4031	300 nM	13	275.3 ± 118.8, p = 0.45	221.9 ± 86.1, p = 0.37	153.1 ± 70.5, p = 0.05	46.5 ± 32.6, p = 0.05
High Risk	E4031	500 nM	13	242.8 ± 114.3, p = 0.78	211 ± 90.7, p = 0.13	123.8 ± 64.3, p = 0.7	27.3 ± 32.4, p = 0.002
Low Risk	Haloperidol	300 nM	27	139 ± 38.3, p = 0.02	129.6 ± 31.9, p = 0.009	115.2 ± 22.6, p = 0.01	89.9 ± 24.1, p = 0.34
Low Risk	Haloperidol	1 µM	27	220.8 ± 78.3, p = 0.005	189.3 ± 57.4, p = 0.04	139.3 ± 43.4, p = 0.41	64.6 ± 37.5, p = 0.29
Low Risk	Haloperidol	3 µM	27	212.9 ± 56.9, p = 0.45	192.8 ± 39.2, p = 0.11	122.4 ± 29.5, p = 0.82	50.5 ± 38.5, p = 0.1
Low Risk	Haloperidol	10 µM	27	163.8 ± 38, p = 0.02	161.7 ± 39.4, p = 0.009	104.1 ± 21.5, p = 1	52.8 ± 37.9, p = 0.003

High Risk	Haloperidol	300 nM	19	203.8 ± 92.8, p = 0.02	167.8 ± 55.3, p = 0.009	140 ± 38.6, p = 0.01	86 ± 22.4, p = 0.34
High Risk	Haloperidol	1 µM	19	275.8 ± 64.8, p = 0.005	218.2 ± 43.7, p = 0.04	150.2 ± 51, p = 0.41	54.4 ± 30.7, p = 0.29
High Risk	Haloperidol	3 µM	19	194.7 ± 38.3, p = 0.45	169.8 ± 32.8, p = 0.11	118.9 ± 40.9, p = 0.82	31.7 ± 28.5, p = 0.1
High Risk	Haloperidol	10 µM	19	132.9 ± 33.5, p = 0.02	125.4 ± 29.3, p = 0.009	99.5 ± 31, p = 1	21.7 ± 23.3, p = 0.003

Changes of field potential duration (FPD) corrected field potential duration (cFPD), RR interval (RR), and peak-to-peak amplitude (PtPA) are presented as percentage of the Vehicle treatment response. Values are presented as mean ± standard deviation. N indicates the number of hiPSC-CMs monolayers included in the analysis. Wilcoxon rank-sum test p-values are provided.

Supplemental Table 11. List of primers used for genetic variant analysis in hiPSCs

Target sequence	Forward/Reverse primer (5′-3′)
KCNQ1 Exon 3	Fw: 5’- gttcaaacaggttgcagggtctga -3’<br>Rev: 5’- ccaggtttccagaccaggaag -3’
KCNQ1 Exon 6	Fw: 5’- tggctgaccactgtccctct -3’<br>Rev: 5’- ccccaggaccccagctgtccaa -3’
KCNQ1 Exon 15	Fw: 5’-ctacctcccccagccctac-3’<br>Rev: 5’-caactcccaagaggggcc-3’
NOS1AP rs16847548	Fw: 5’- aggggaacttaaaccgtgcc -3’<br>Rev: 5’- agcgccctctatcaccaatg -3’
KCNH2 exon 5	Fw: 5’- ccatggactcctgactgtg-3’<br>Rev: 5’-tggaatagattgccaacccc -3’
KCNH2 exon 7	Fw: 5’-cgtgctgttcttgctcatgt-3’<br>Rev: 5’-agcagcctcagtttcctcc-3’
KCNH2 exon 10	Fw: 5’-cagctgaggggacatgctc-3’<br>Rev.: 5’-accatttgacagacaggga-3’
KCNH2 exon 12	Fw: 5’-ttcctgcccagtcctctctc-3’<br>Rev: 5’-gacactcctgagaaggcgc-3’

Supplemental Table 12. List of antibodies used for immunofluorescence staining of pluripotency markers

	Antibody	Dilution	Company and Cat #
Primary	Rabbit anti Nanog	1:200	Stemgent Cat# 09-0020,
	Mouse anti Oct3/4 (C-10)	1:500	SCBT Cat# sc-5279
	Rabbit anti Sox2	1:200	Stemgent Cat# 09-0024
	Mouse anti TRA-1-60	1:200	Invitrogen Cat# MA1-023
	Rat anti SSEA-3 (631)	1:100	SCBT Cat# sc-21703
	Mouse anti SSEA-4 (MC-813-70)	1:200	Invitrogen Cat# 414000,
Secondary	Alexa-Fluor® 488 Goat anti-rat IgM	1:500	ThermoFisher Cat# A21212
	Alexa-Fluor® 488 Goat anti-mouse IgG	1:500	ThermoFisher Cat# A11001
	Alexa-Fluor® 546 Goat anti-mouse IgG	1:500	ThermoFisher Cat# A11003
	Alexa-Fluor® 546 Goat anti-rabbit IgG	1:500	ThermoFisher Cat# A11010

Supplemental figures

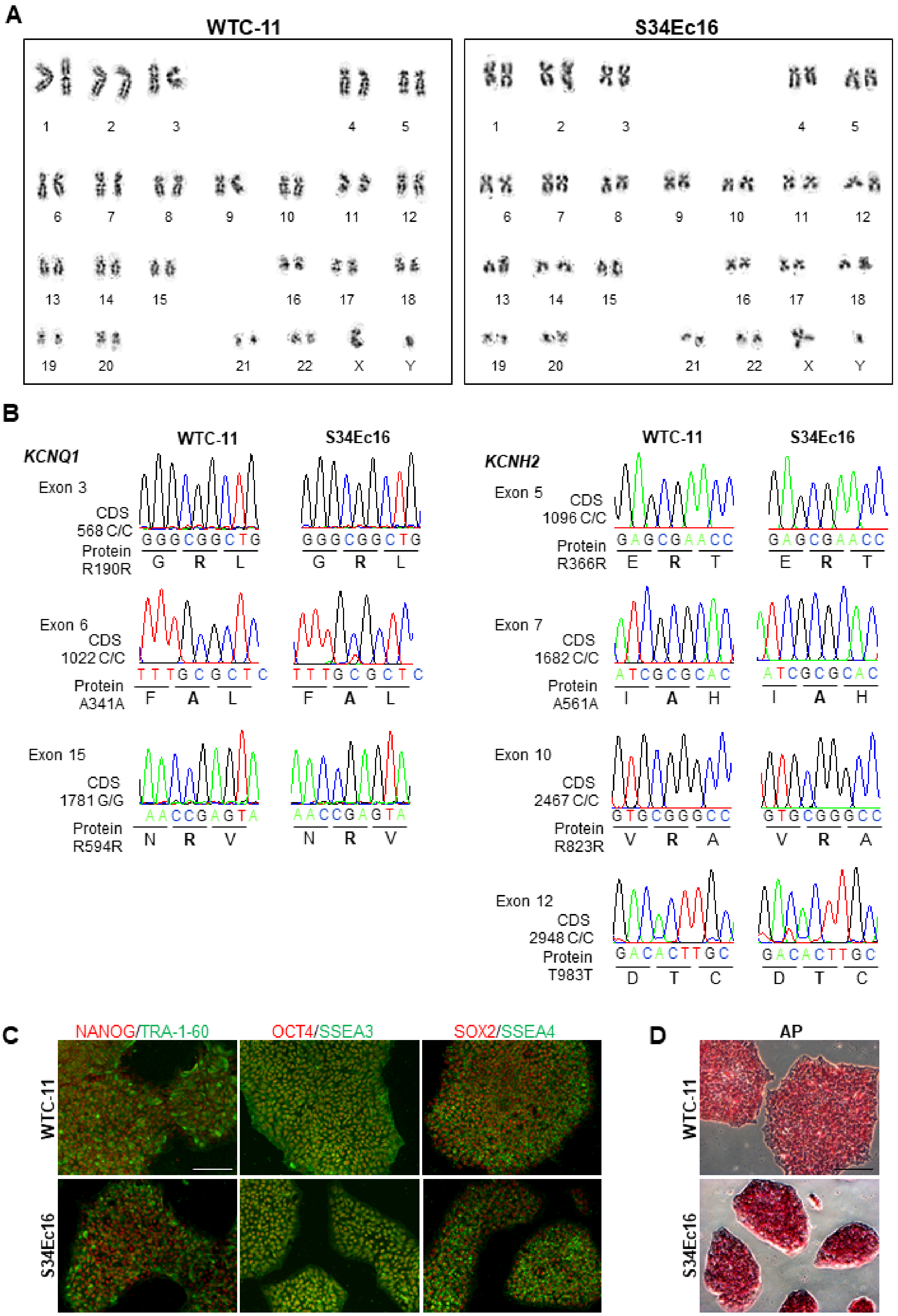

Supplemental Figure 1. Characterization of wild-type hiPSC lines WTC-11 and S34Ec16.

A. DNA karyotyping performed using 300 G-banding chromosome analysis, showing male karyotype (46, XY) in both hiPSC lines. B. DNA sequencing results of Exons 3, 6 and 15 of the *KCNQ1* gene (left), and Exons 5, 7, 10 and 12 of the *KCNH2* gene (right), showing a WT genotype in both WTC-11 and S34Ec16 hiPSC genomic DNA. C. Immunofluorescence stainings showing uniform expression of the indicated markers of pluripotency in WTC-11 and S34Ec16 hiPSCs. Nuclear transcription factors are in red, membrane antigens are in green. Scale bars 100 µm. D. Alkaline phosphatase colorimetric staining (AP). Scale bars 100 µm.

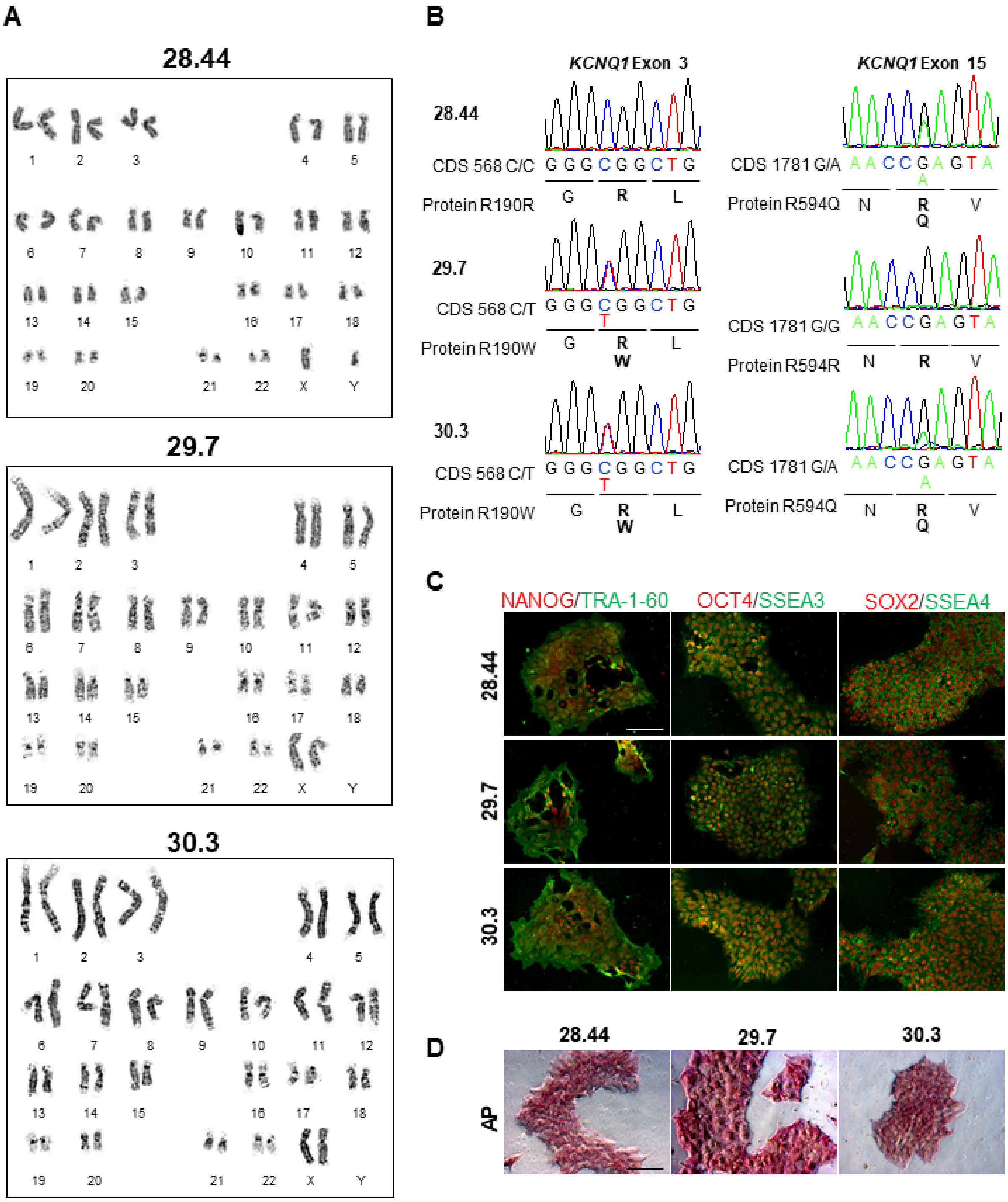

Supplemental Figure 2. Characterization of hiPSC lines 28.44, 29.7 and 30.3 from the LQT1 family trio carrying *KCNQ1* p.R594Q, *KCNQ1* p.R190W, and *KCNQ1* p.R190W & p.R594Q variants. A. DNA karyotyping performed using 300 G-banding chromosome analysis, showing male karyotype (46, XY) in 28.44 hiPSC, and female (46, XX) karyotype in 29.7 and 30.3 hiPSCs. B. DNA sequencing results showing the heterozygous variant 568

C/T in *KCNQ1* Exon 3 of 29.7 and 30.3 hiPSCs genomic DNA, and the heterozygous variant 1781 G/A in *KCNQ1* Exon 15 of 28.44 and 30.3 hiPSCs genomic DNA. C. Immunofluorescence stainings showing uniform expression of the indicated markers of pluripotency in hiPSCs. Nuclear transcription factors are in red, membrane antigens are in green. Scale bars 100 μm. D. Alkaline phosphatase colorimetric staining (AP). Scale bars 100 μm.

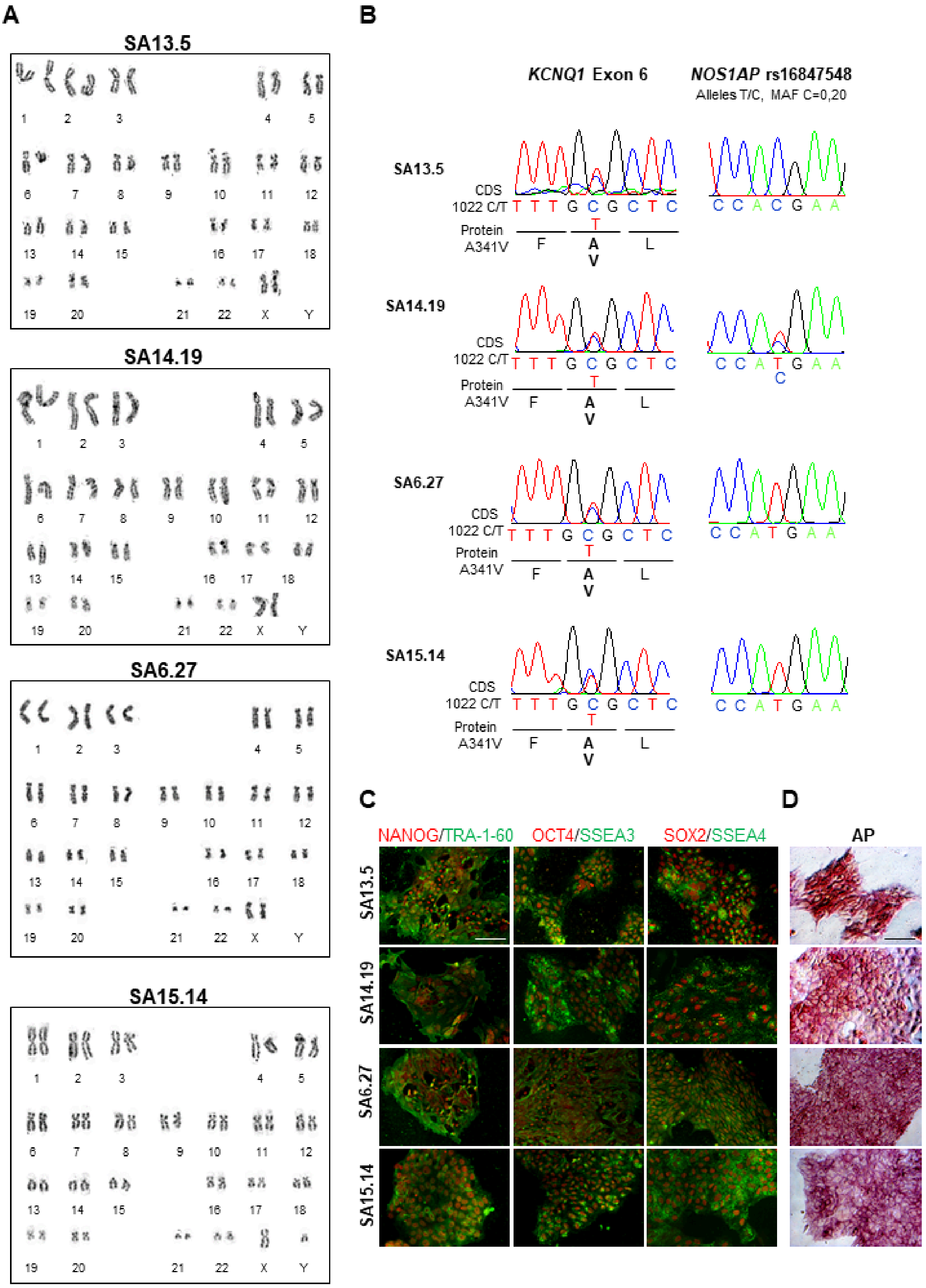
A
SA13.5
SA14.19
SA6.27
SA15.14
B
KCNQ1 Exon 6
NOS1AP rs16847548
Alleles T/C, MAF C=0,20
CDS
1022 C/T
Protein
A341V
C
NANOG/TRA-1-60
OCT4/SSEA3
SOX2/SSEA4
D
AP

Supplemental Figure 3. Characterization of LQT1 hiPSC lines SA13.5, SA6.27, SA14.19, and SA15.14 from the South African family carrying *KCNQ1* p.A341V variant. A. DNA karyotyping performed using 300 G-banding chromosome analysis, showing female (46, XX) karyotype in SA13.5, SA6.27 and SA14.19 hiPSCs, and male karyotype (46, XY) in SA15.14 hiPSC. B. DNA sequencing results: on the left the heterozygous 1022 C/T variant in *KCNQ1* Exon 6 of SA13.5, SA14.19, SA6.27, and SA15.14 hiPSCs genomic DNA; on the right the *NOS1AP* rs16847548 minor allele in homozygosis in SA13.5 hiPSC and in heterozygosis in SA14.19 hiPSC, and the rs16847548 major allele in homozygosis in SA6.27 and SA15.14 hiPSCs genomic DNA. MAF is the minor allele frequency in the South African founder population. C. Immunofluorescence stainings showing uniform expression of the indicated markers of pluripotency in hiPSCs. Nuclear transcription factors are in red, membrane antigens are in green. Scale bars 100 μm. D. Alkaline phosphatase colorimetric staining (AP). Scale bars 100 μm.

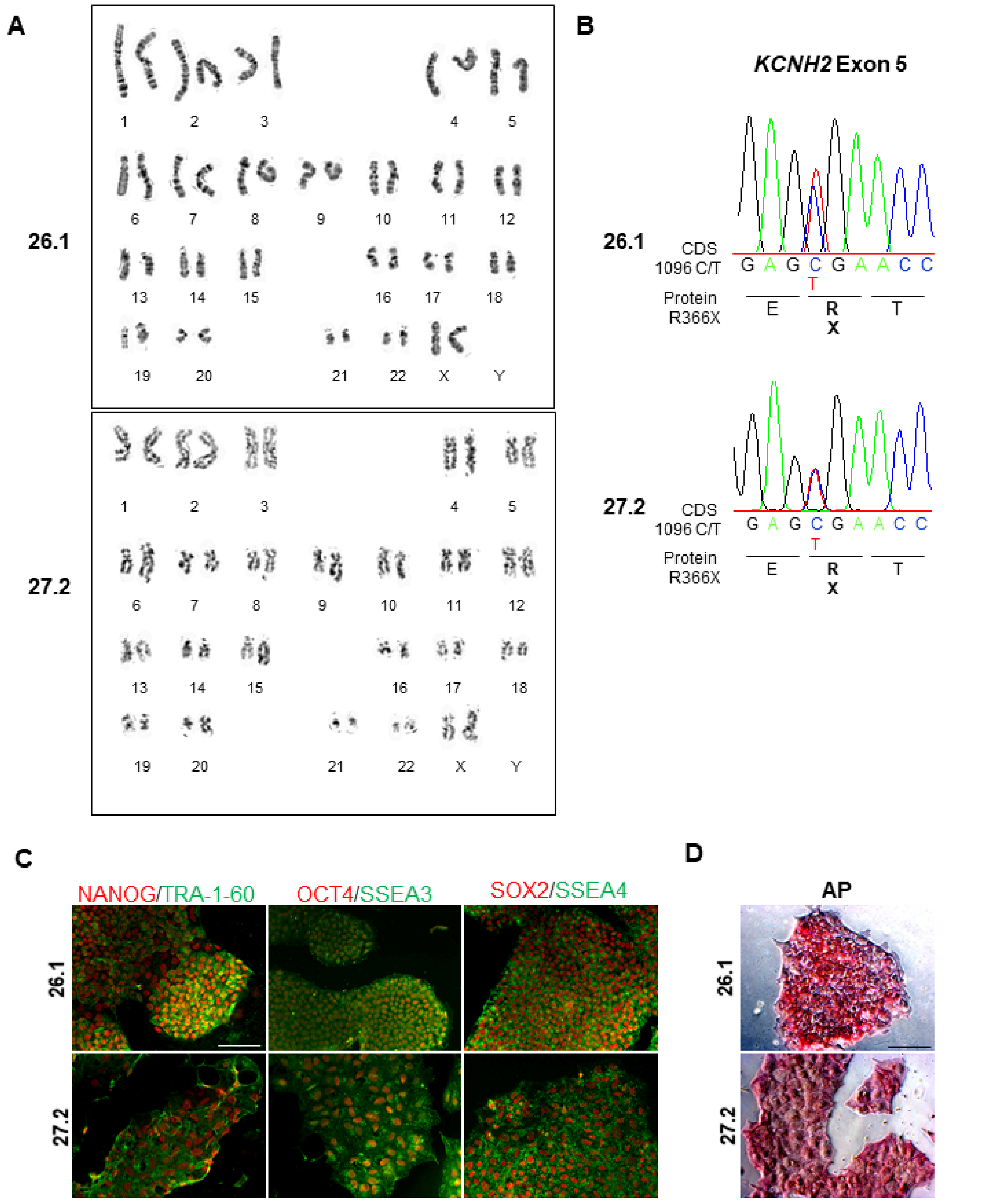

Supplemental Figure 4. Characterization of LQT2 hiPSC lines 26.1 and 27.2, carrying the *KCNH2* R366X variant. A. DNA karyotyping performed using 300 G-banding chromosome analysis, showing female (46, XX) karyotype in both 26.1 and 27.2 hiPSCs. B. DNA sequencing results showing the variant 1096 C/T in the *KCNH2* exon 5 in heterozygosis in 26.1 and 27.2 hiPSC DNA. C. Immunofluorescence stainings showing

uniform expression of the indicated markers of pluripotency in the 26.1 and 27.2 hiPSCs. Nuclear transcription factors are in red, membrane antigens are in green. Scale bars 100 μm. D. Alkaline phosphatase colorimetric staining (AP). Scale bars 100 μm.

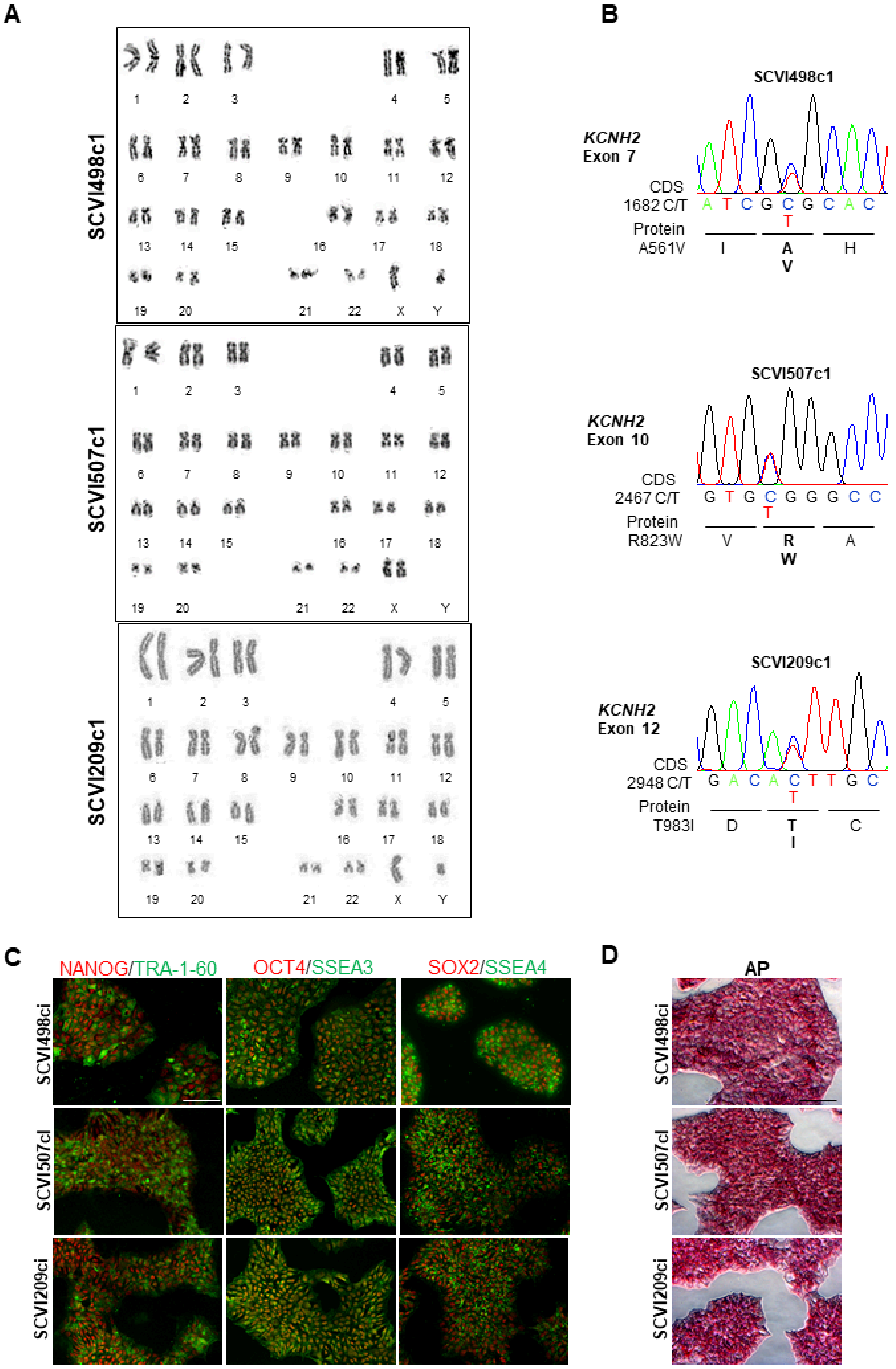
A
SCVI498c1
SCVI507c1
SCVI209c1
1 2 3 4 5 6 7 8 9 10 11 12 13 14 15 16 17 18 19 20 21 22 X Y
B
SCVI498c1
KCNH2
Exon 7
CDS
1682 C/T
Protein
A561V
SCVI507c1
KCNH2
Exon 10
CDS
2467 C/T
Protein
R823W
SCVI209c1
KCNH2
Exon 12
CDS
2948 C/T
Protein
T983I
C
NANOG/TRA-1-60
OCT4/SSEA3
SOX2/SSEA4
SCVI498ci
SCVI507ci
SCVI209ci
D
AP

Supplemental Figure 5. Characterization of LQT2 hiPSC lines SCVI498c1 (*KCNH2* A561V), SCVI507c1 (*KCNH2* R823W), and SCVI209c1 (*KCNH2* T983I). A. DNA karyotyping performed using 300 G-banding chromosome analysis, showing male karyotype (46, XY) in SCVI498ci and SCVI209ci hiPSCs, and female karyotype (46, XX) in SCVI507ci hiPSCs. B. DNA sequencing results showing the following heterozygous variants: 1682 C/T in the *KCNH2* exon 7 of SCVI498c1 hiPSC genomic DNA, 2467 C/T in the *KCNH2* exon 10 of SCVI507c1 hiPSC genomic DNA, and 2948 C/T in the *KCNH2* exon 12 of SCVI209c1 hiPSC genomic DNA. C. Immunofluorescence stainings showing uniform expression of the indicated markers of pluripotency in hiPSCs. Nuclear transcription factors are in red, membrane antigens are in green. Scale bars 100 µm. D. Alkaline phosphatase colorimetric staining (AP). Scale bars 100 µm.

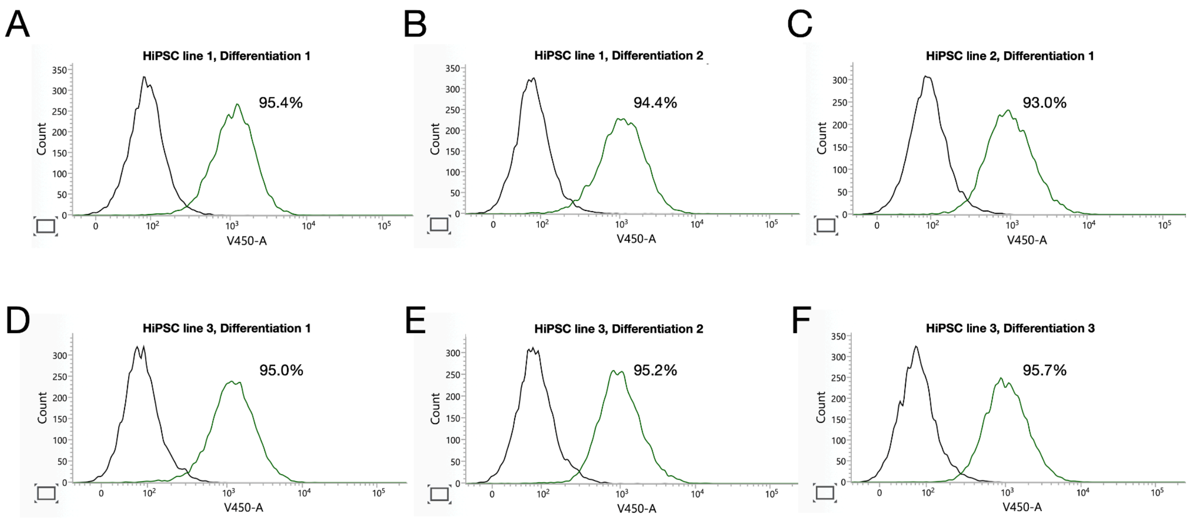

Supplemental Figure 6. Flow cytometry quantification of hiPSC-CM purity. A-F. The percentage of cardiac troponin T (cTnT)-positive cells was analyzed across six independent differentiations of three randomly selected hiPSC-CM (indicated next to each histogram). Histograms represent the distribution of fluorescence intensity for anti-cTnT V450 antibody stained cells (green line) compared with unstained controls (black line).

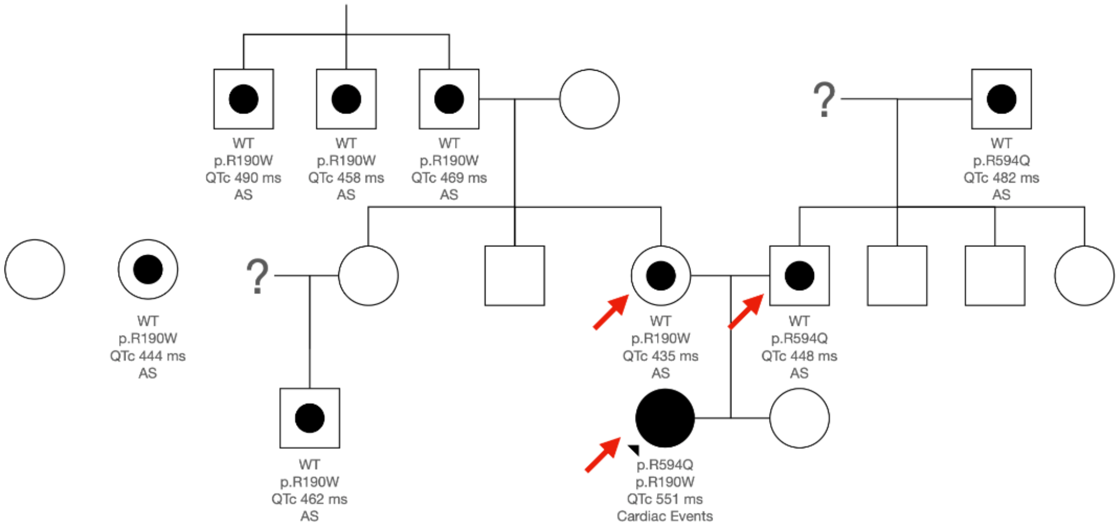

Supplemental Figure 7. Pedigree of LQT1-affected family, with the studied family. Trio carrying genetic variants *KCNQ1* p.R190W and *KCNQ1* p.R594Q is indicated by red arrows.

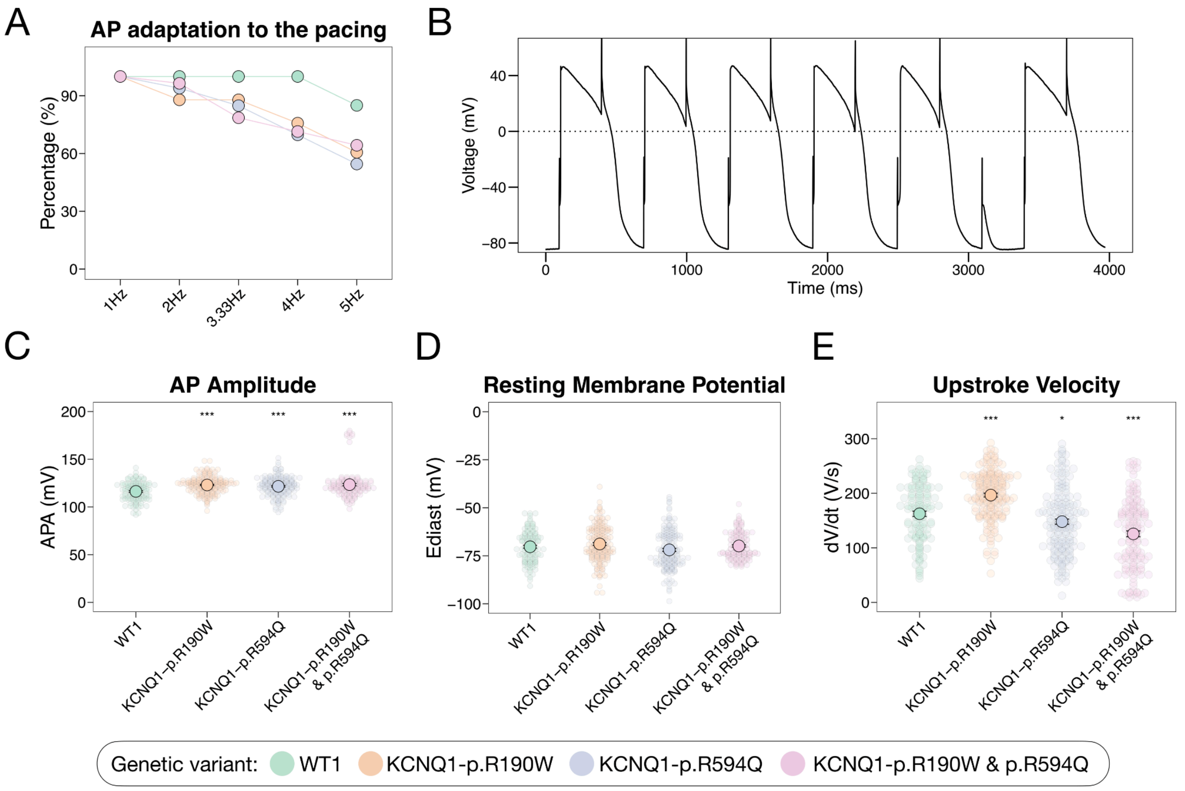

Supplemental Figure 8. Characterization of hiPSC-CMs derived from family trio carrying genetic variants *KCNQ1* p.R190W and *KCNQ1* p.R594Q. Color defines hiPSC-CMs carrying different genetic variants: green – wild-type (WT1), orange – *KCNQ1* p.R190W, blue – *KCNQ1* p.R594Q, and pink – *KCNQ1* p.R190W & p.R594Q. A. Percentage of hiPSC-CMs adapted to the increasing frequency pacing rate. B. Example of AP recording from hiPSC-CM carrying *KCNQ1* p.R190W & p.R594Q genetic variants not able to follow the 3 Hz pacing. C. hiPSC-CM action potential amplitude (APA). D. hiPSC-CM resting membrane potential. F. hiPSC-CM upstroke velocity. Wilcoxon rank-sum test vs WT1 group $^*p \leq 0.05$, $^{**}p \leq 0.01$, $^{***}p \leq 0.001$.

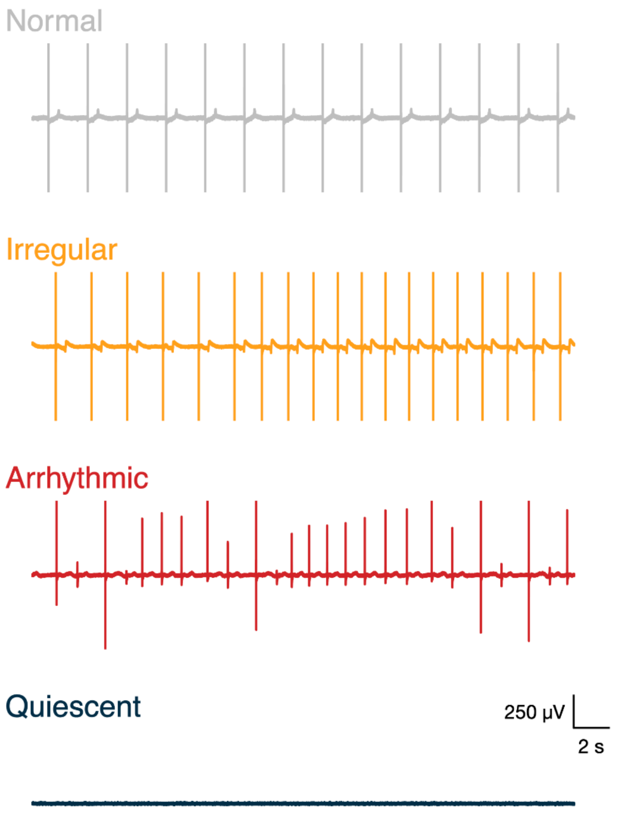

Supplemental Figure 9. Representative field potential recordings corresponding to different beating pattern abnormalities. Color defines following beating patterns: Normal (gray), Irregular (yellow), Arrhythmic (red), Quiescent (black).

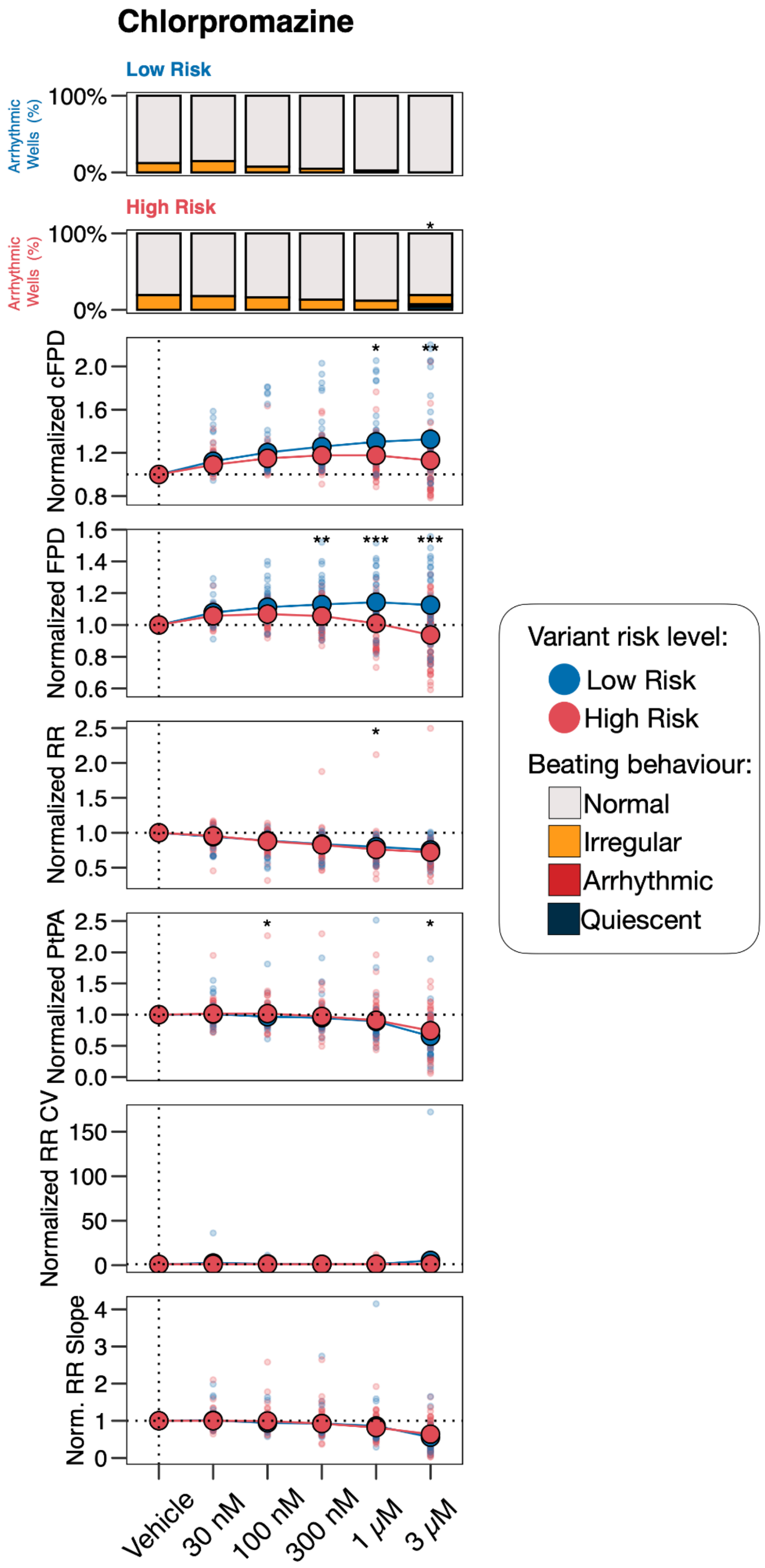

Supplemental Figure 10. Chlorpromazine MEA concentration-response curves of hiPSC-CMs from Low Risk and High Risk groups. Wilcoxon rank-sum test for two group

comparison, Fisher's exact test for categorical variables comparison. $*p \leq 0.05$, $**p \leq 0.01$, $***p \leq 0.001$.

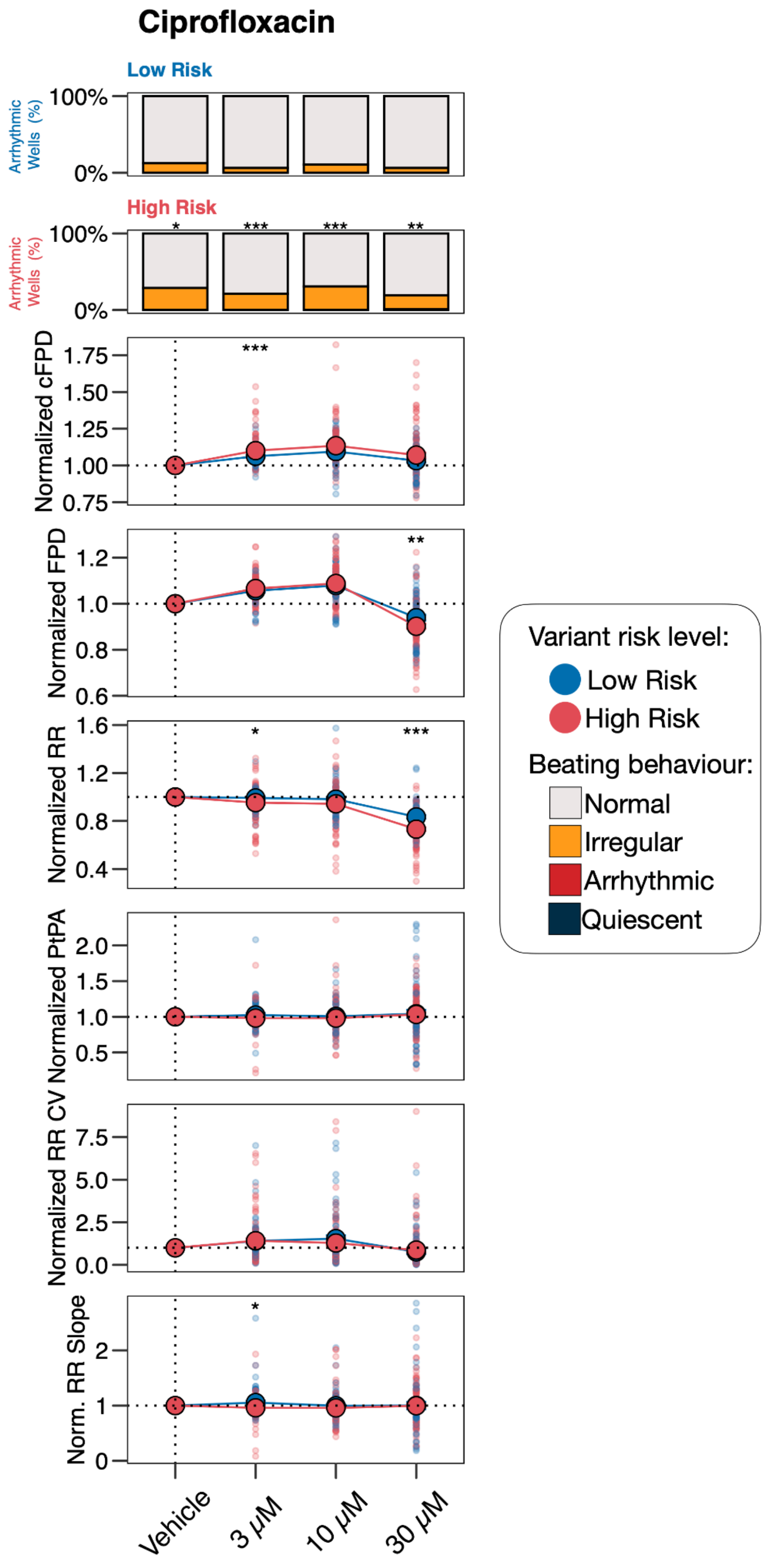

Supplemental Figure 11. Ciprofloxacin MEA concentration-response curves of hiPSC-CMs from Low Risk and High Risk groups. Wilcoxon rank-sum test for two group comparison, Fisher's exact test for categorical variables comparison. $*p \leq 0.05$, $**p \leq 0.01$, $***p \leq 0.001$.

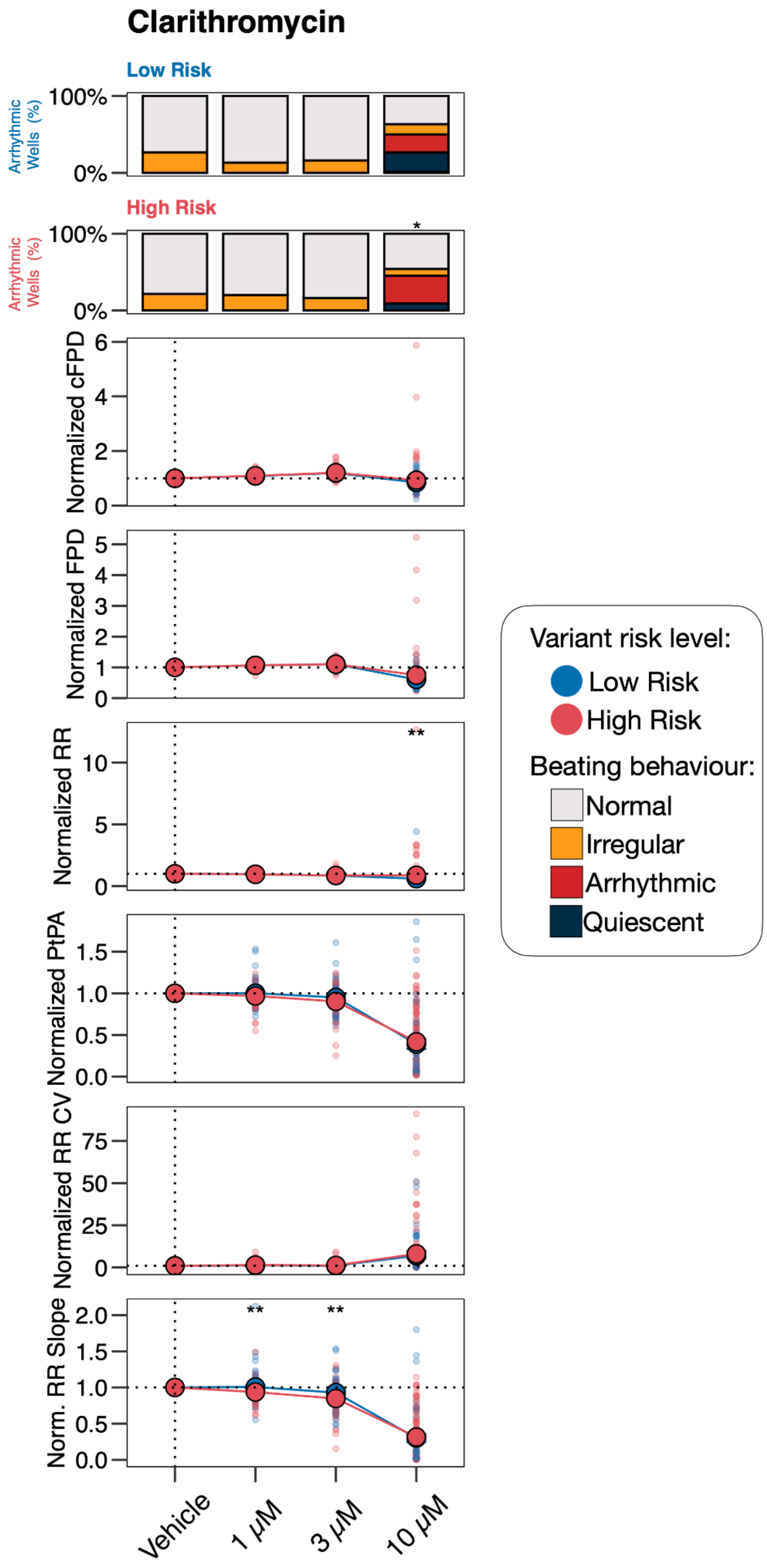

Supplemental Figure 12. Clarithromycin MEA concentration-response curves of hiPSC-CMs from Low Risk and High Risk groups. Wilcoxon rank-sum test for two group

comparison, Fisher's exact test for categorical variables comparison. *$p \leq 0.05$, **$p \leq 0.01$, ***$p \leq 0.001$.

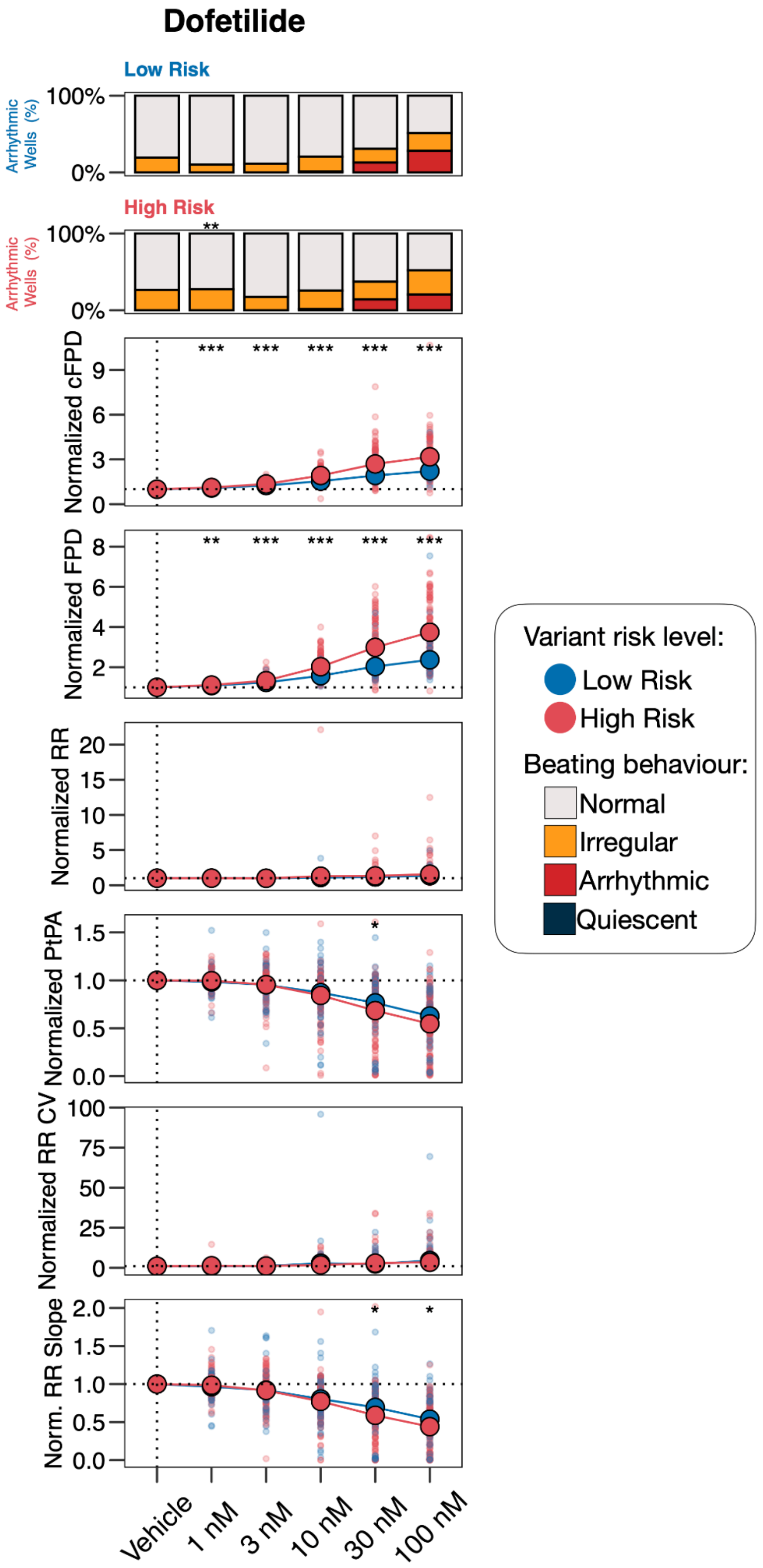

Supplemental Figure 13. Dofetilide MEA concentration-response curves of hiPSC-CMs from Low Risk and High Risk groups. Wilcoxon rank-sum test for two group comparison, Fisher's exact test for categorical variables comparison. *p ≤ 0.05, **p ≤ 0.01, ***p ≤ 0.001.

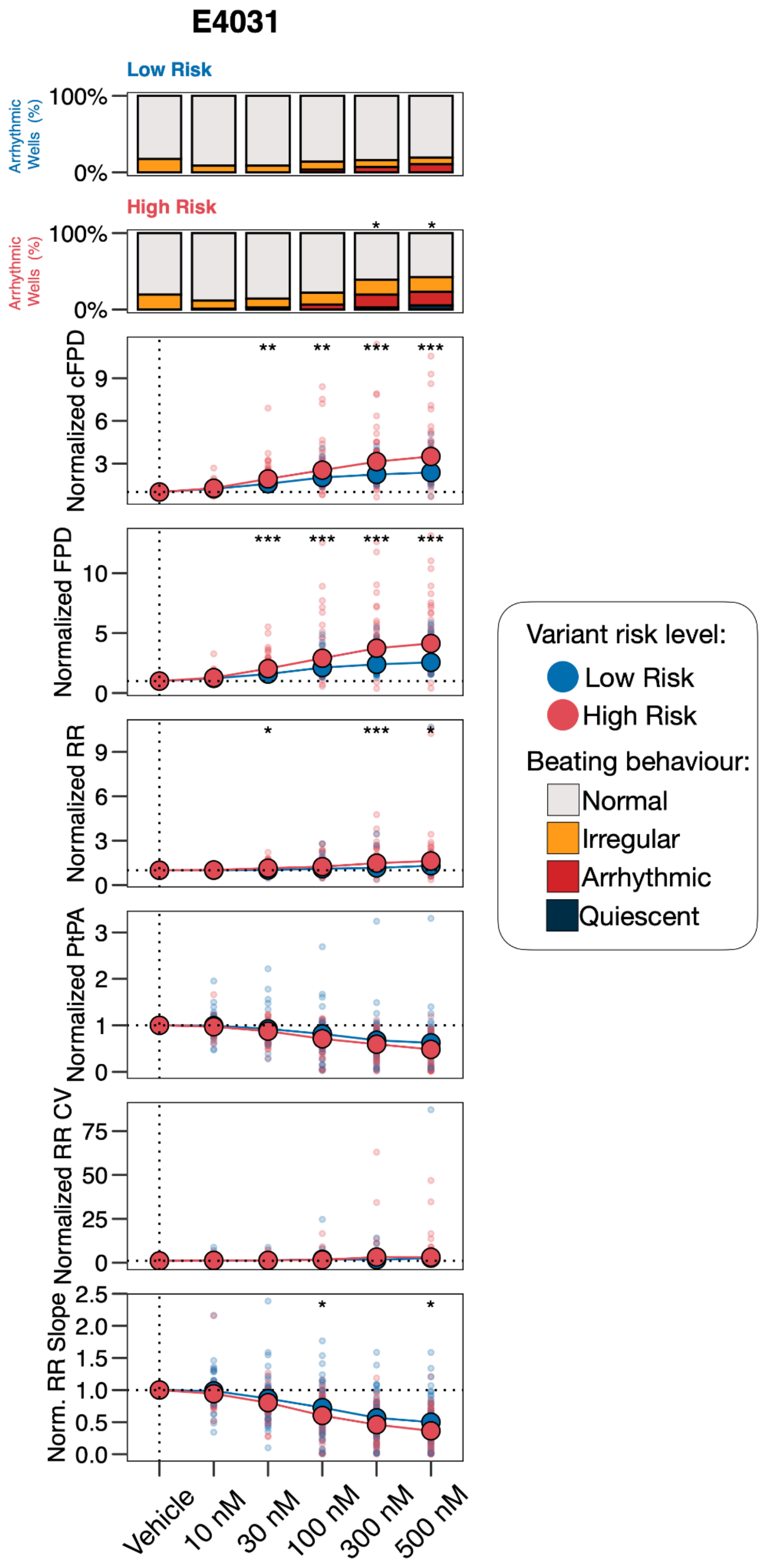

Supplemental Figure 14. E4031 MEA concentration-response curves of hiPSC-CMs from Low Risk and High Risk groups. Wilcoxon rank-sum test for two group comparison, Fisher's exact test for categorical variables comparison. *p ≤ 0.05, **p ≤ 0.01, ***p ≤ 0.001.

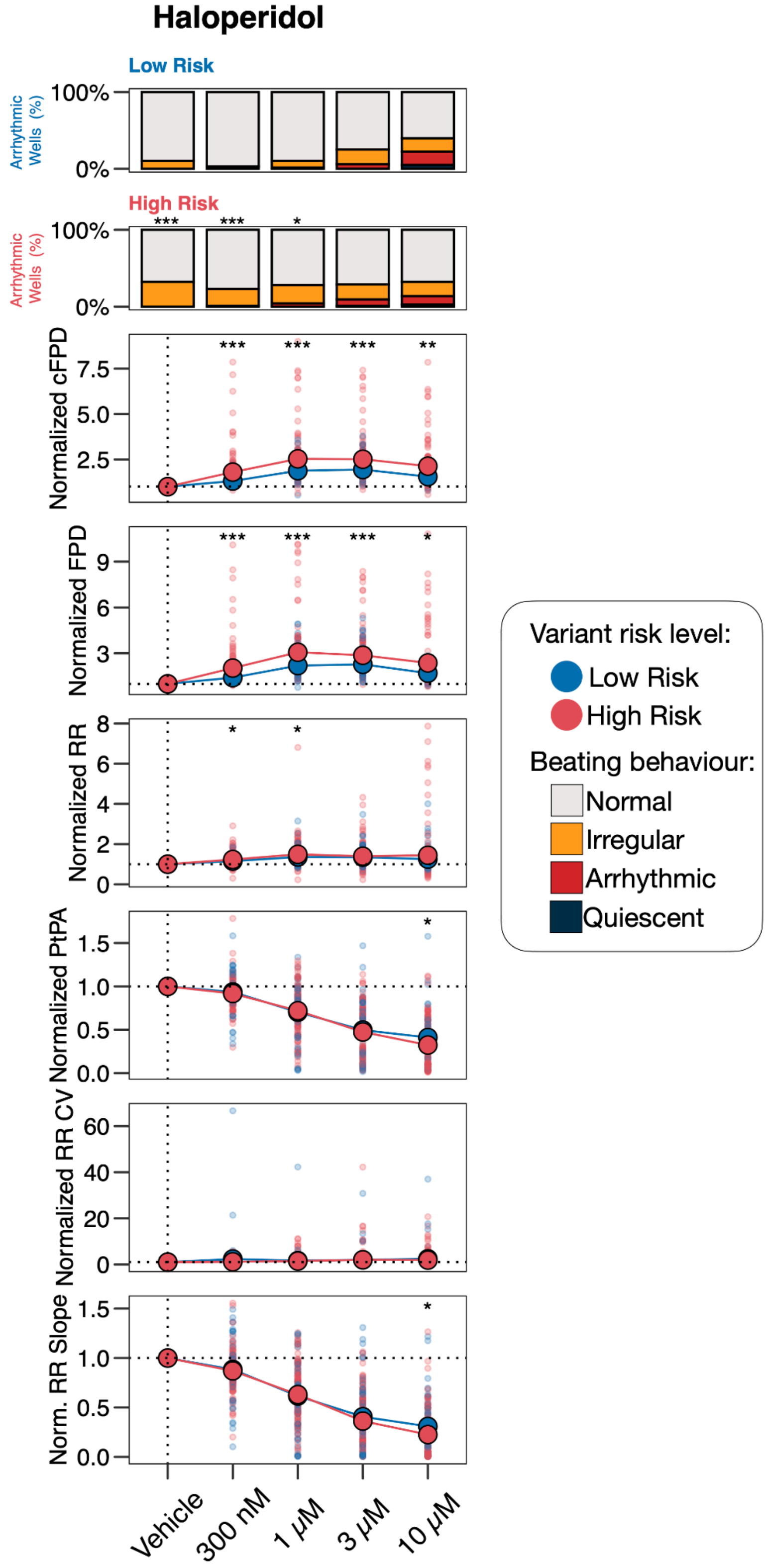

Supplemental Figure 15. Haloperidol MEA concentration-response curves of hiPSC-CMs from Low Risk and High Risk groups. Wilcoxon rank-sum test for two group comparison, Fisher's exact test for categorical variables comparison. $*p \leq 0.05$, $**p \leq 0.01$, $***p \leq 0.001$.

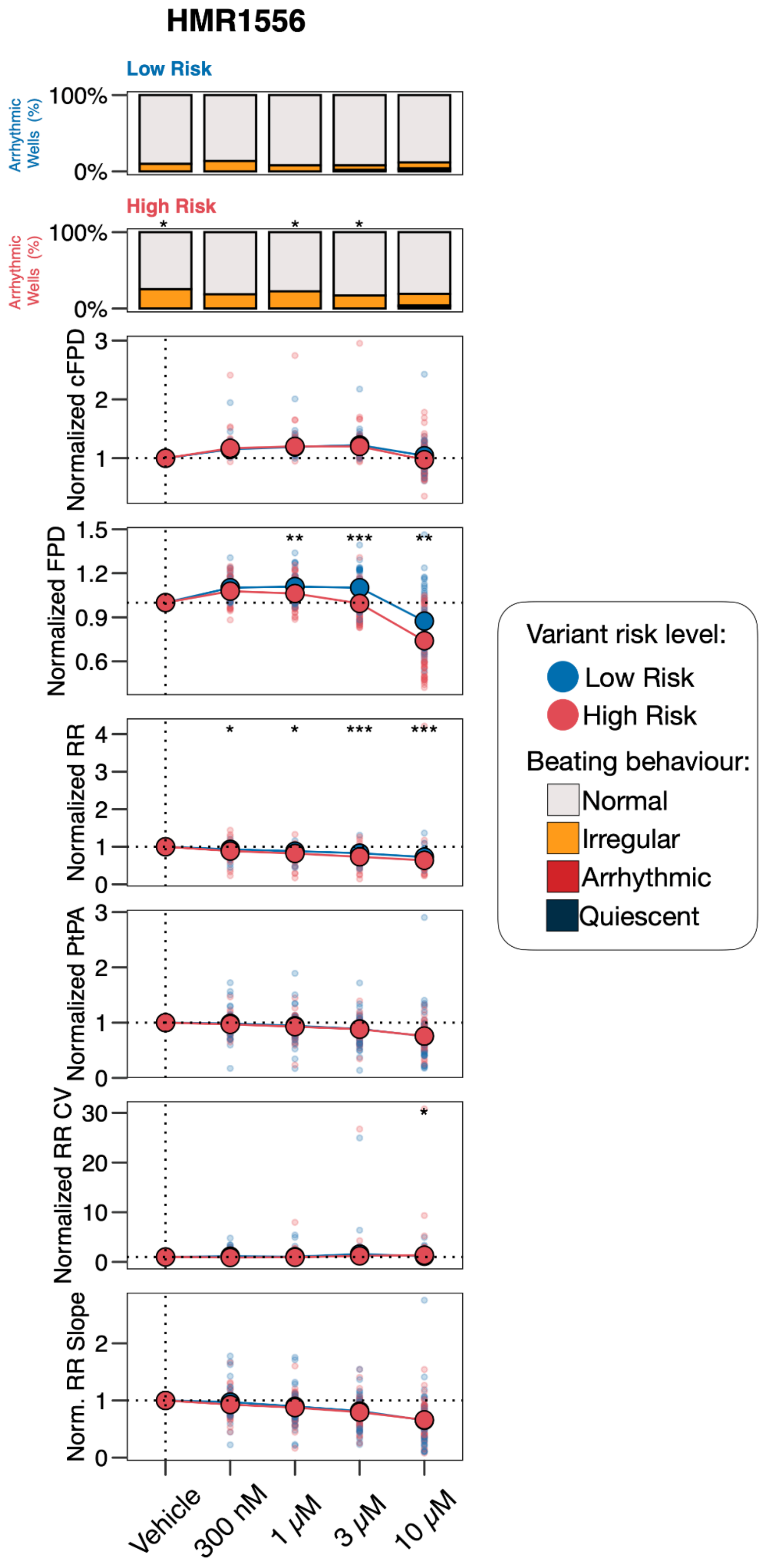

Supplemental Figure 16. HMR1556 MEA concentration-response curves of hiPSC-CMs from Low Risk and High Risk groups. Wilcoxon rank-sum test for two group comparison, Fisher's exact test for categorical variables comparison. $*p \leq 0.05$, $**p \leq 0.01$, $***p \leq 0.001$.

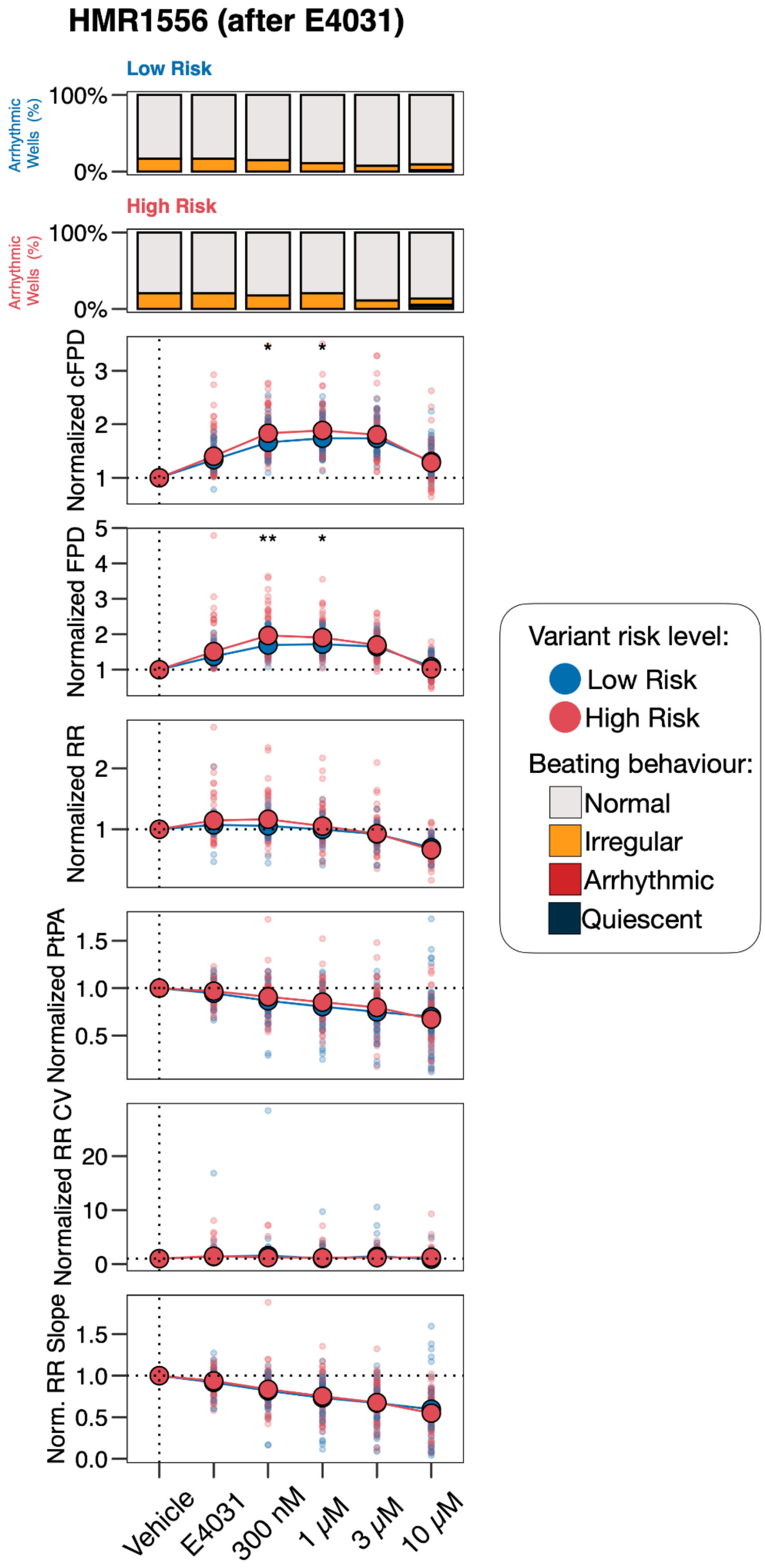

Supplemental Figure 17. HMR1556 after E4031 pretreatment MEA concentration-response curves of hiPSC-CMs from Low Risk and High Risk groups.

Wilcoxon rank-sum test for two group comparison, Fisher's exact test for categorical variables comparison. *$p \le 0.05$, **$p \le 0.01$, ***$p \le 0.001$.

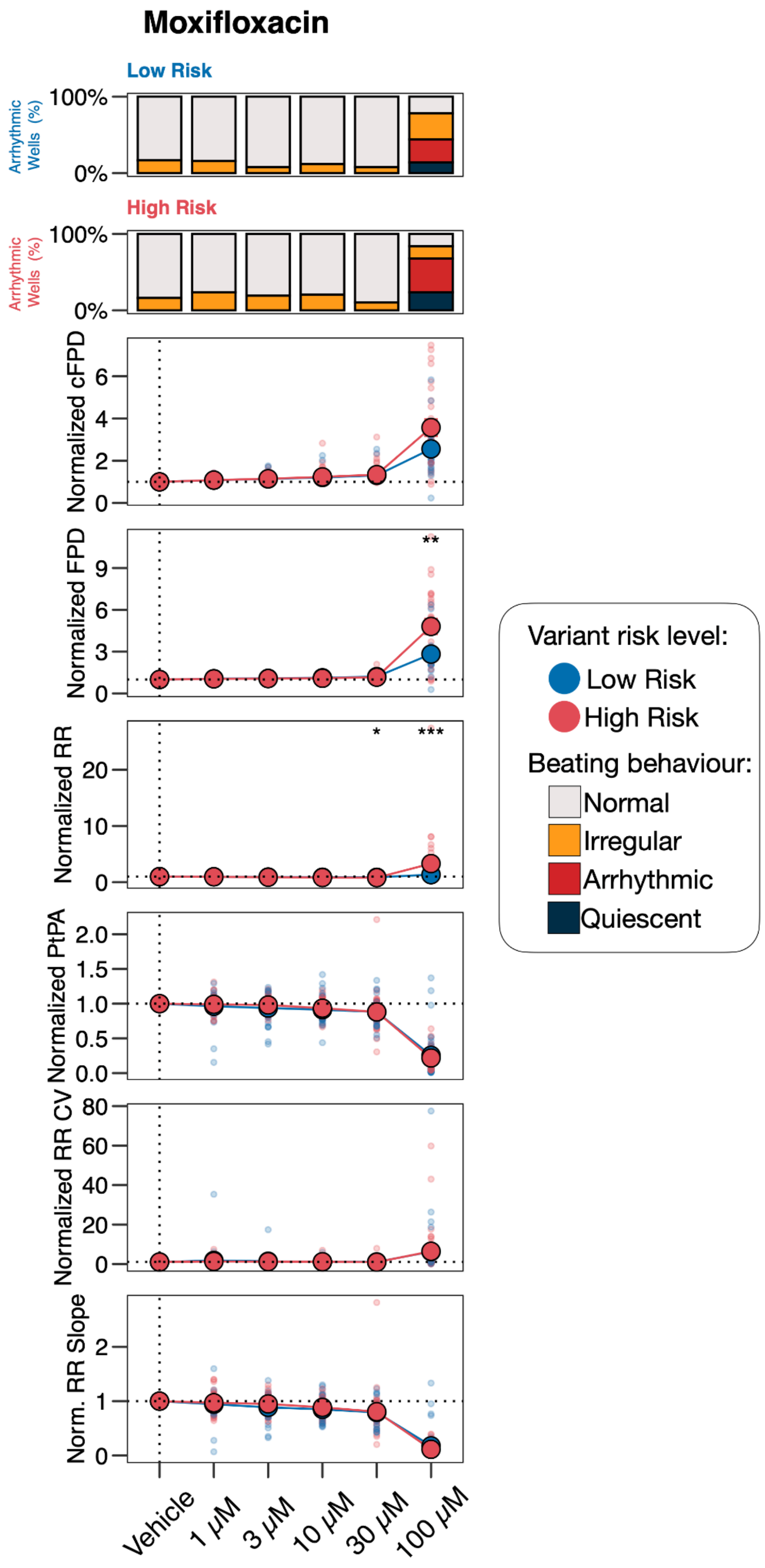

Supplemental Figure 18. Moxifloxacin MEA concentration-response curves of hiPSC-CMs from Low Risk and High Risk groups. Wilcoxon rank-sum test for two group

comparison, Fisher's exact test for categorical variables comparison. *$p \leq 0.05$, **$p \leq 0.01$, ***$p \leq 0.001$.

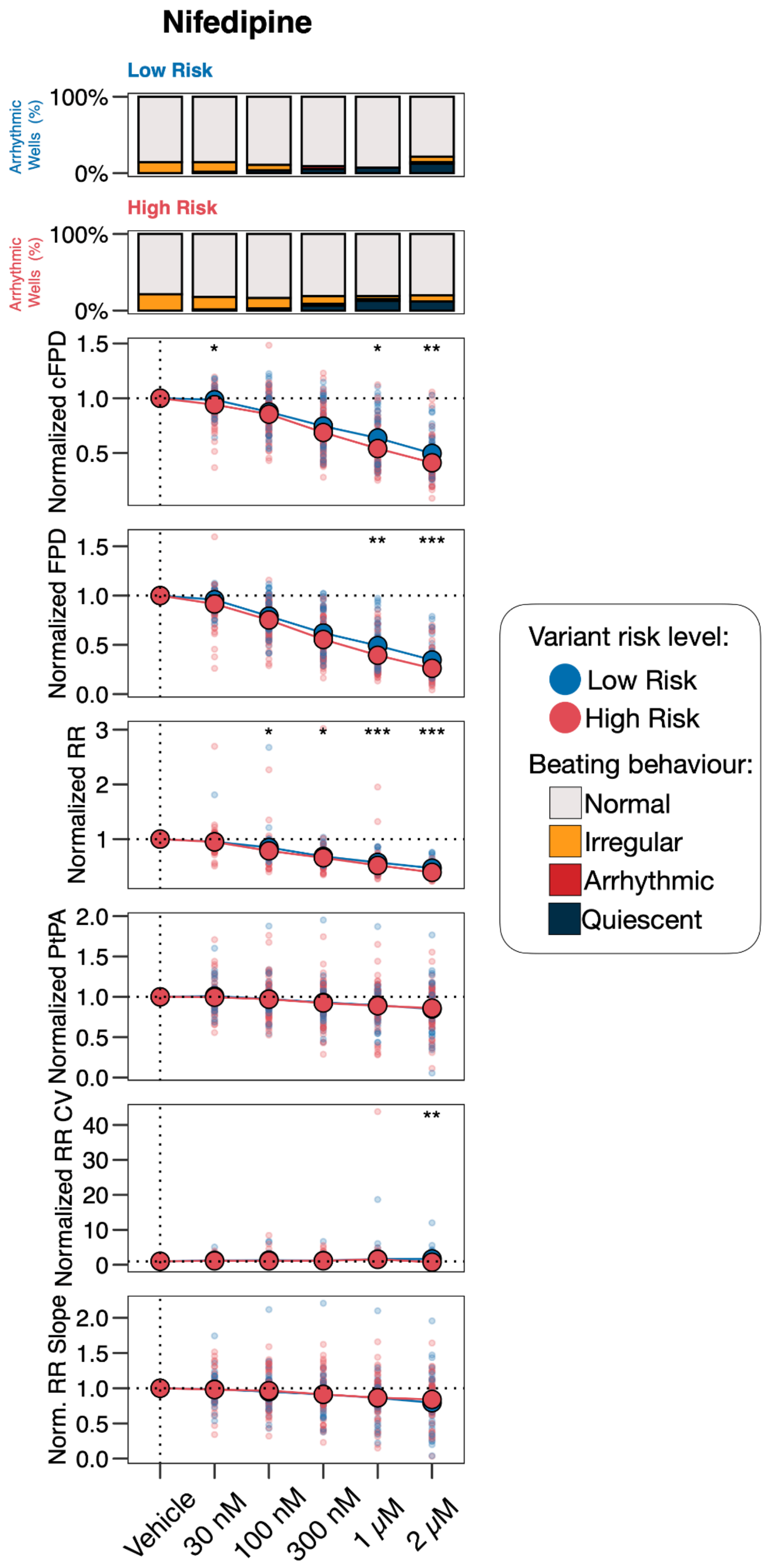

Supplemental Figure 19. Nifedipine MEA concentration-response curves of hiPSC-CMs from Low Risk and High Risk groups. Wilcoxon rank-sum test for two group comparison, Fisher's exact test for categorical variables comparison. *p ≤ 0.05, **p ≤ 0.01, ***p ≤ 0.001.

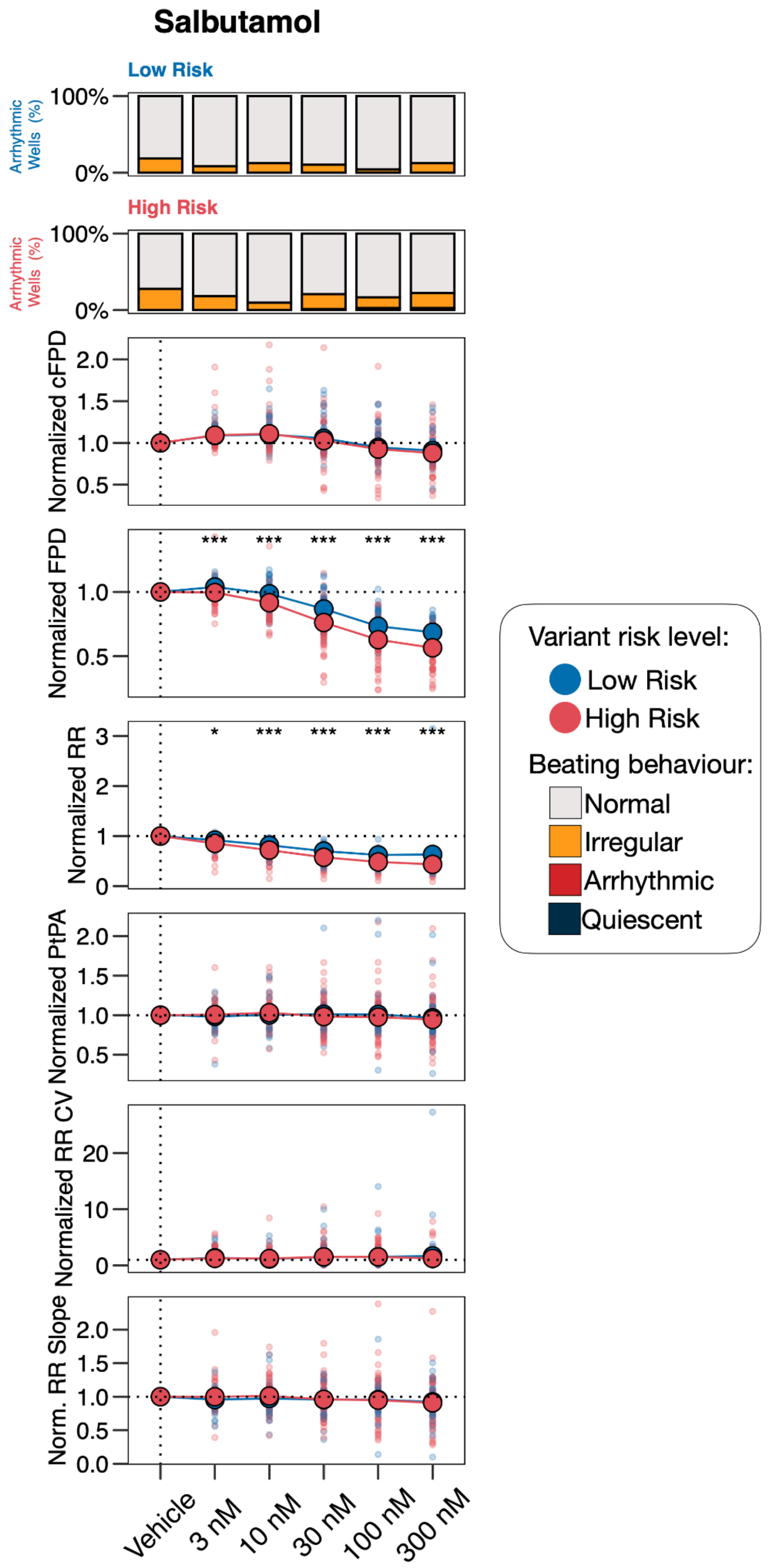

Supplemental Figure 20. Salbutamol MEA concentration-response curves of hiPSC-CMs from Low Risk and High Risk groups. Wilcoxon rank-sum test for two group comparison, Fisher's exact test for categorical variables comparison. *$p \leq 0.05$, **$p \leq 0.01$, ***$p \leq 0.001$.

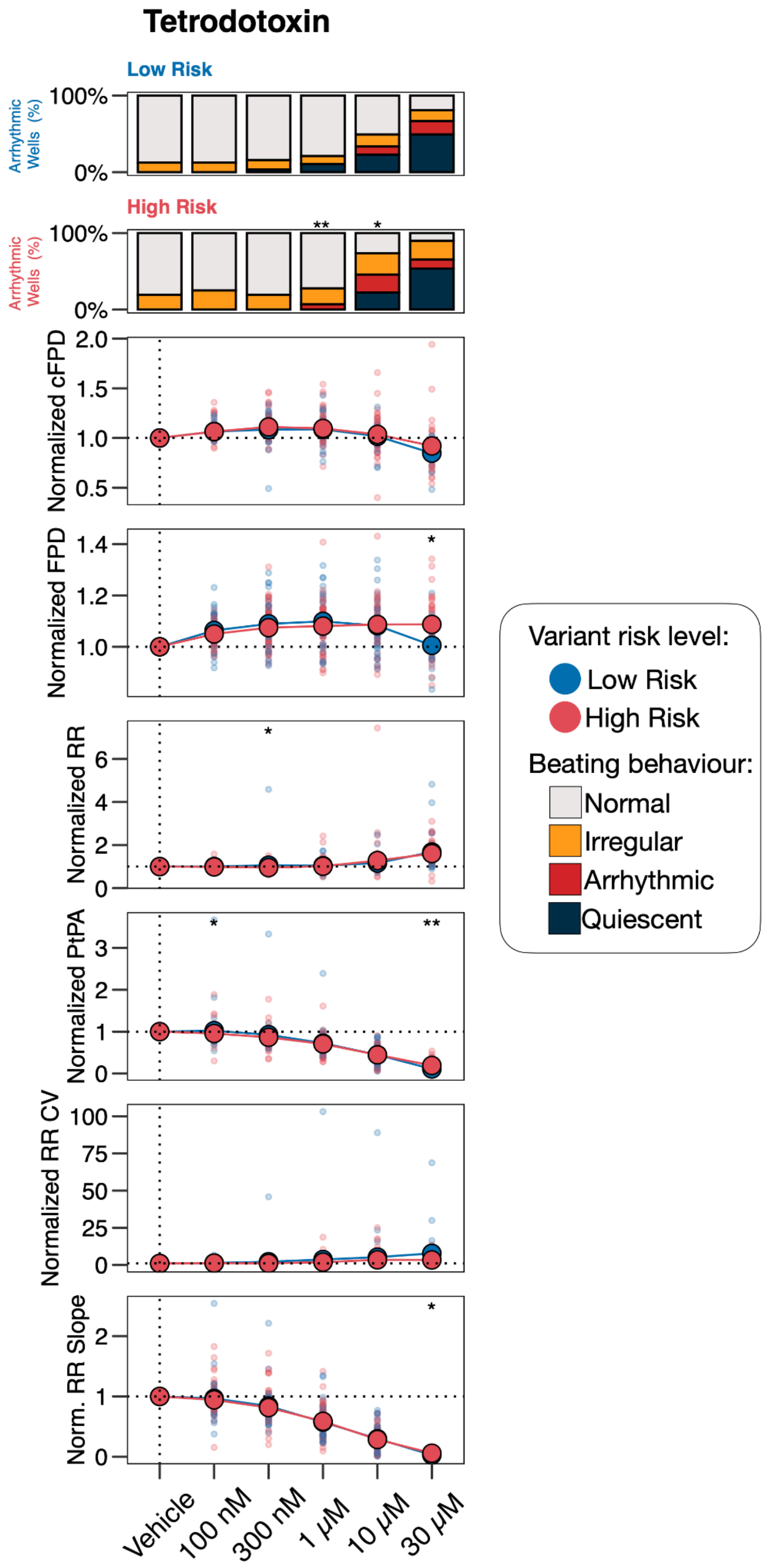

Supplemental Figure 21. Tetrodotoxin MEA concentration-response curves of hiPSC-CMs from Low Risk and High Risk groups. Wilcoxon rank-sum test for two group

comparison, Fisher's exact test for categorical variables comparison. $*p \leq 0.05$, $**p \leq 0.01$, $***p \leq 0.001$.

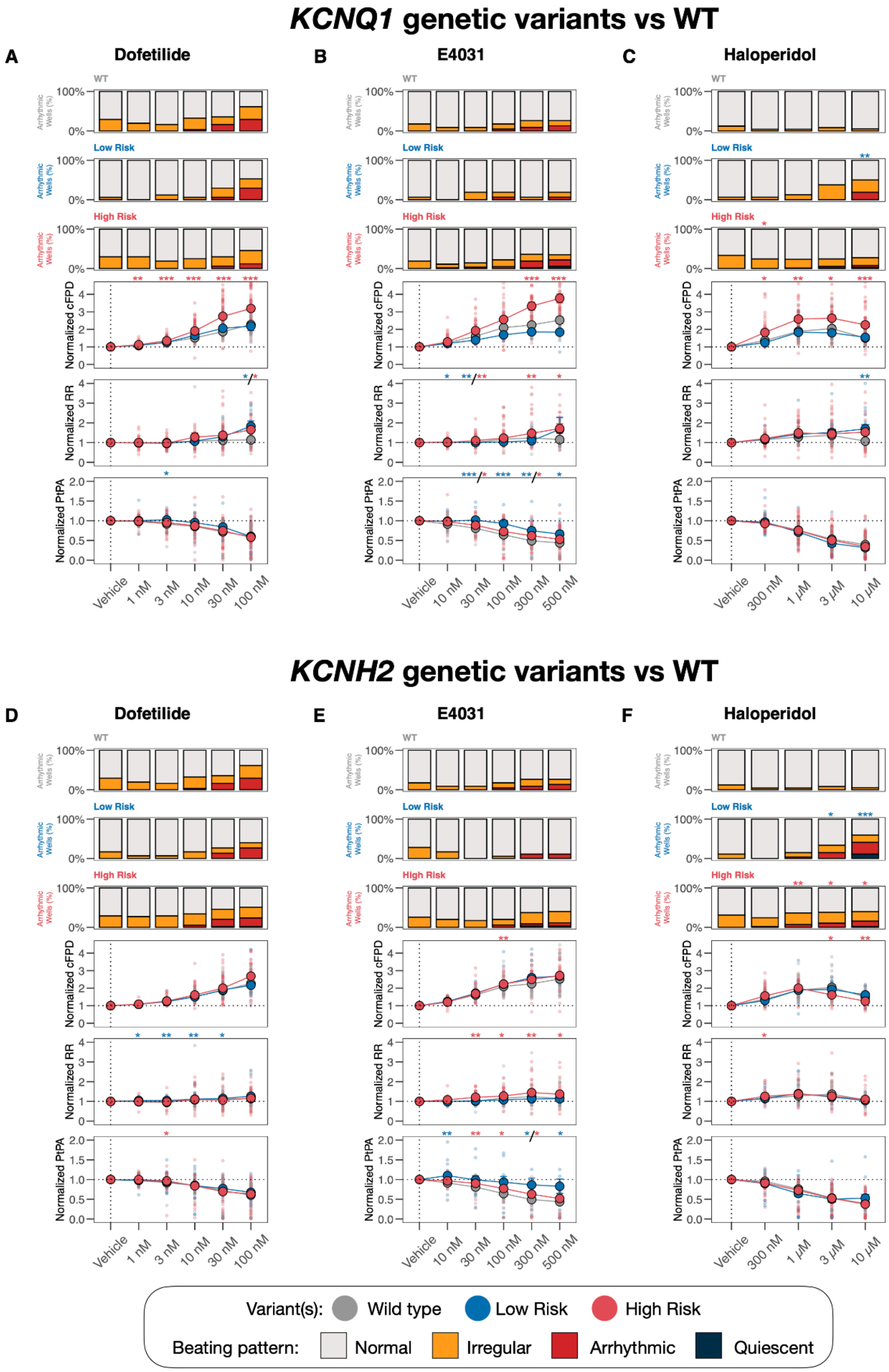
KCNQ1 genetic variants vs WT
A Dofetilide
B E4031
C Haloperidol
KCNH2 genetic variants vs WT
D Dofetilide
E E4031
F Haloperidol
WT
Low Risk
High Risk
Arrhythmic Wells (%)
100%
0%
Normalized cFPD
Normalized RR
Normalized PtPA
Vehicle
1 nM
3 nM
10 nM
30 nM
100 nM
300 nM
500 nM
300 nM
1 µM
3 µM
10 µM
Variant(s): Wild type Low Risk High Risk
Beating pattern: Normal Irregular Arrhythmic Quiescent

Supplemental Figure 22. Drug responses of WT hiPSC-CMs are similar to those of hiPSC-CMs carrying Low Risk *KCNQ1* and *KCNH2* variants. Electrophysiological readouts from hiPSC-CMs corresponding to the Low Risk group, High Risk group, and two WT hiPSC-CMs were pooled to generate Low Risk, High Risk and WT cohorts. A-F: Concentration-dependent effects of dofetilide, E4031, and haloperidol on normalized corrected field potential duration (normalized cFPD), normalized RR interval (normalized RR), and normalized peak-to-peak amplitude (normalized PtPA). Bar plots illustrate the distribution of beating pattern abnormalities. Asterisks indicate statistically significant differences for the High Risk vs WT (red asterisks) and Low Risk vs WT comparisons (blue asterisks). N = 16-101 per variant/group per drug concentration. Wilcoxon rank-sum test for two group comparison. Fisher's exact test for categorical variables comparison. *$p \leq 0.05$, **$p \leq 0.01$, ***$p \leq 0.001$.

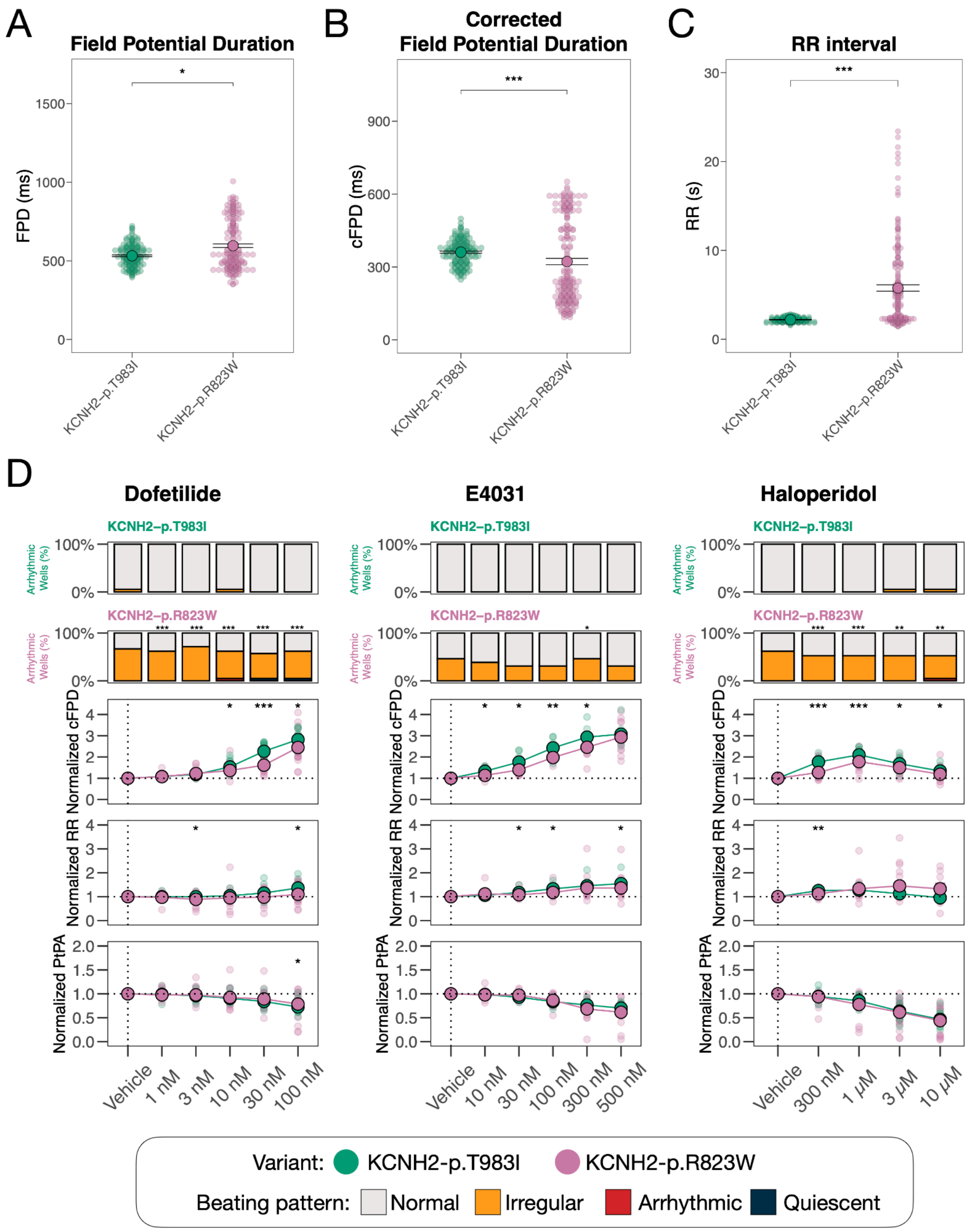

Supplemental Figure 23. Baseline electrophysiology and drug responses of LQT2 hiPSC-CMs carrying *KCNH2* p.T983I and *KCNH2* p.R823W variants. A-C: Baseline field potential duration (FPD), corrected FPD (cFPD), and beat-to-beat interval (RR) measurements of hiPSC-CMs for each variant. N = 143-174. D. Concentration-dependent effects of dofetilide, E4031, and haloperidol on normalized corrected field potential duration (normalized cFPD), normalized RR interval (normalized RR), and normalized peak-to-peak

amplitude (normalized PtPA). Bar plots depict the distribution of beating pattern abnormalities. Asterisks indicate statistically significant differences between variants. N = 9-21 per variant per drug concentration. Statistical analyses were performed the Wilcoxon rank-sum test for two-group comparisons, and Fisher's exact test for categorical variables. *p ≤ 0.05, **p ≤ 0.01, ***p ≤ 0.001.

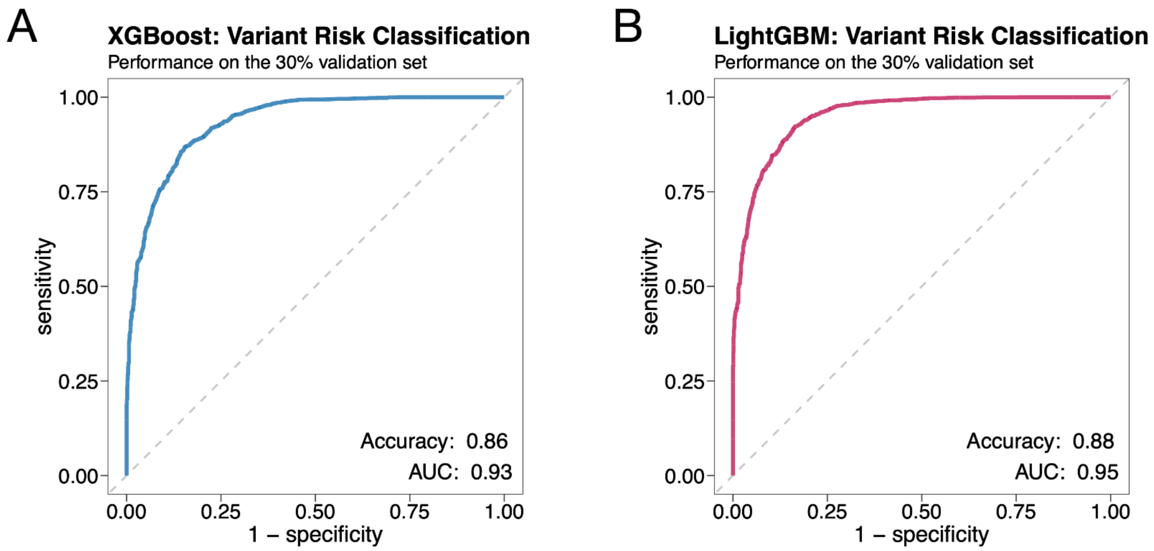

Supplemental Figure 24. Comparison of XGBoost and LightGBM classification models. A. Receiver operating characteristic (ROC) curve for the XGBoost classifier. B. ROC curve for the LightGBM classifier.

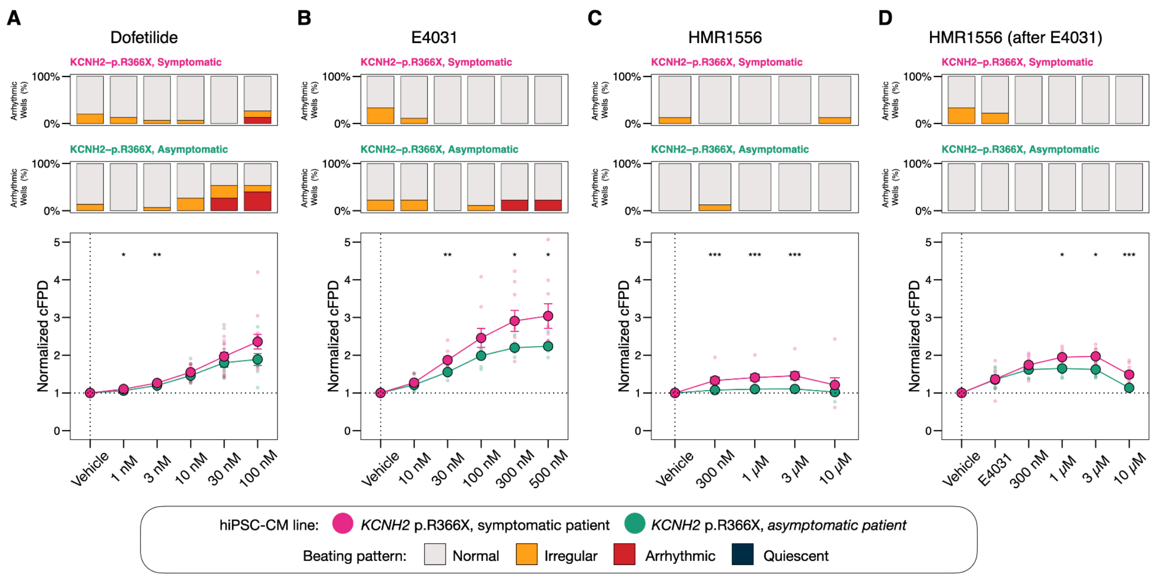

Supplemental Figure 25. HiPSC-CMs carrying *KCNH2* p.R366X variant demonstrate hiPSC-CM line-specific drug responses. A-D: Concentration-dependent response curves on normalized corrected field potential duration (normalized cFPD) for dofetilide, E4031, HMR1556, HMR1556 following E4031 pre-treatment in two hiPSC-CM lines carrying the *KCNH2* p.R366X variant from symptomatic and asymptomatic carriers. Bar plots illustrate the distribution of beating pattern abnormalities. Asterisks indicate statistically significant differences between cell lines. N = 8-15 per line per drug concentration. Wilcoxon rank-sum test for two group comparison, Fisher's exact test for categorical variables comparison. *$p \leq 0.05$, **$p \leq 0.01$, ***$p \leq 0.001$.

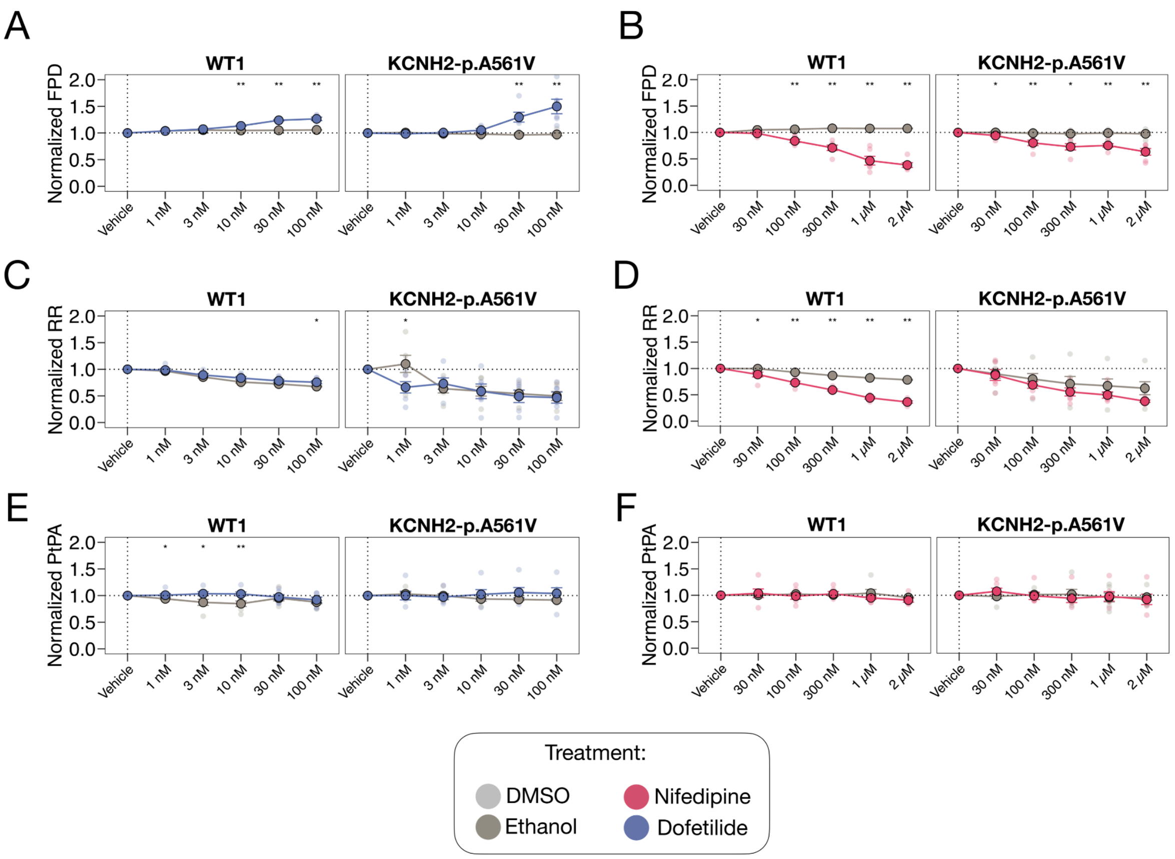

Supplemental Figure 26. Cumulative treatment with DMSO and ethanol vehicles has minimal effects on electrophysiological readouts. A, C, E: Concentration-response curves for dofetilide and the corresponding DMSO vehicle in wild-type hiPSC-CMs (WT) and *KCNH2* p.A561V hiPSC-CMs. B, D, F: Concentration-response curves for nifedipine and the corresponding ethanol vehicle in wild-type hiPSC-CMs (WT1) and *KCNH2* p.A561V hiPSC-CMs. Responses are shown for normalized field potential duration (Normalized FPD), normalized RR interval (Normalized RR), and normalized peak-to-peak amplitude (Normalized PtPA). Asterisks indicate statistically significant differences between drug-treated and vehicle-treated groups. N = 6 per group per drug concentration. Statistical analyses were performed using the Wilcoxon rank-sum test. $*p \leq 0.05$, $**p \leq 0.01$, $***p \leq 0.001$.

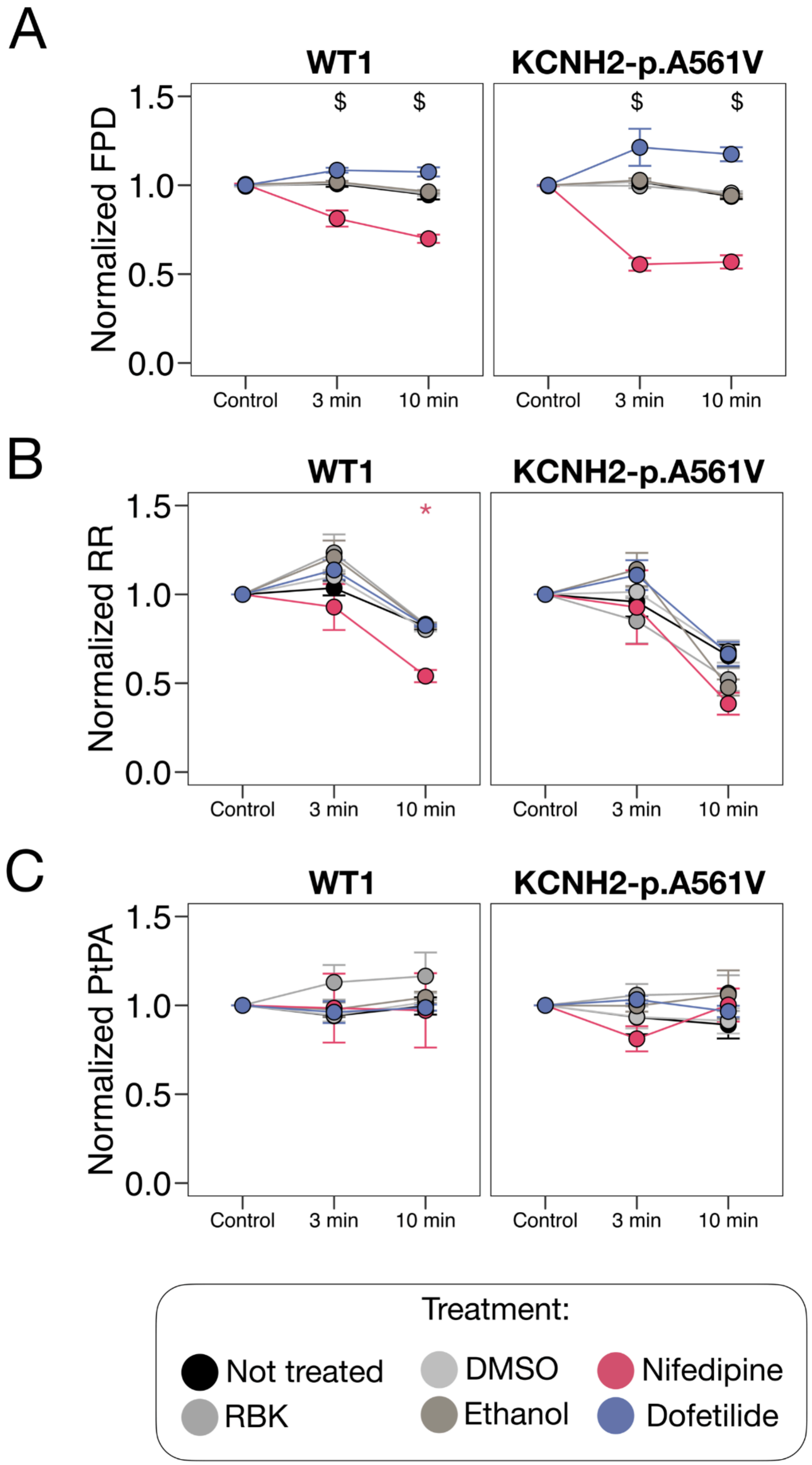
A
WT1
KCNH2-p.A561V
Normalized FPD
1.5
1.0
0.5
0.0
$
$
$
$
Control
3 min
10 min
B
WT1
KCNH2-p.A561V
Normalized RR
*
C
WT1
KCNH2-p.A561V
Normalized PtPA
Treatment:
Not treated
DMSO
Nifedipine
RBK
Ethanol
Dofetilide

Supplemental Figure 27. Electrophysiological readouts exhibit minimal time-dependent effects. Time-course experiments were analyzed at three time points (Control, 3 min, and 10 min after drug addition) to assess potential time-dependent changes. The following conditions were tested: (1) no medium perturbation (not treated), (2) medium control (RBK), (3) ethanol, and (4) DMSO. Two drugs with opposing effects on repolarization were selected: dofetilide and nifedipine. Experiments were performed using hiPSC-CMs from two independent lines: a wild-type (WT1) line and a high-risk *KCNH2* p.A561V line. Statistical analyses (Wilcoxon rank-sum test) compared each drug with its corresponding vehicle (dofetilide vs DMSO and nifedipine vs ethanol). The dollar sign ($) indicates statistical significance ($p \leq 0.05$) for both drug-vehicle comparisons, whereas red asterisks indicate statistical significance only for the nifedipine vs ethanol comparison ($p \leq 0.05$). N = 4 per group per drug per timepoint.

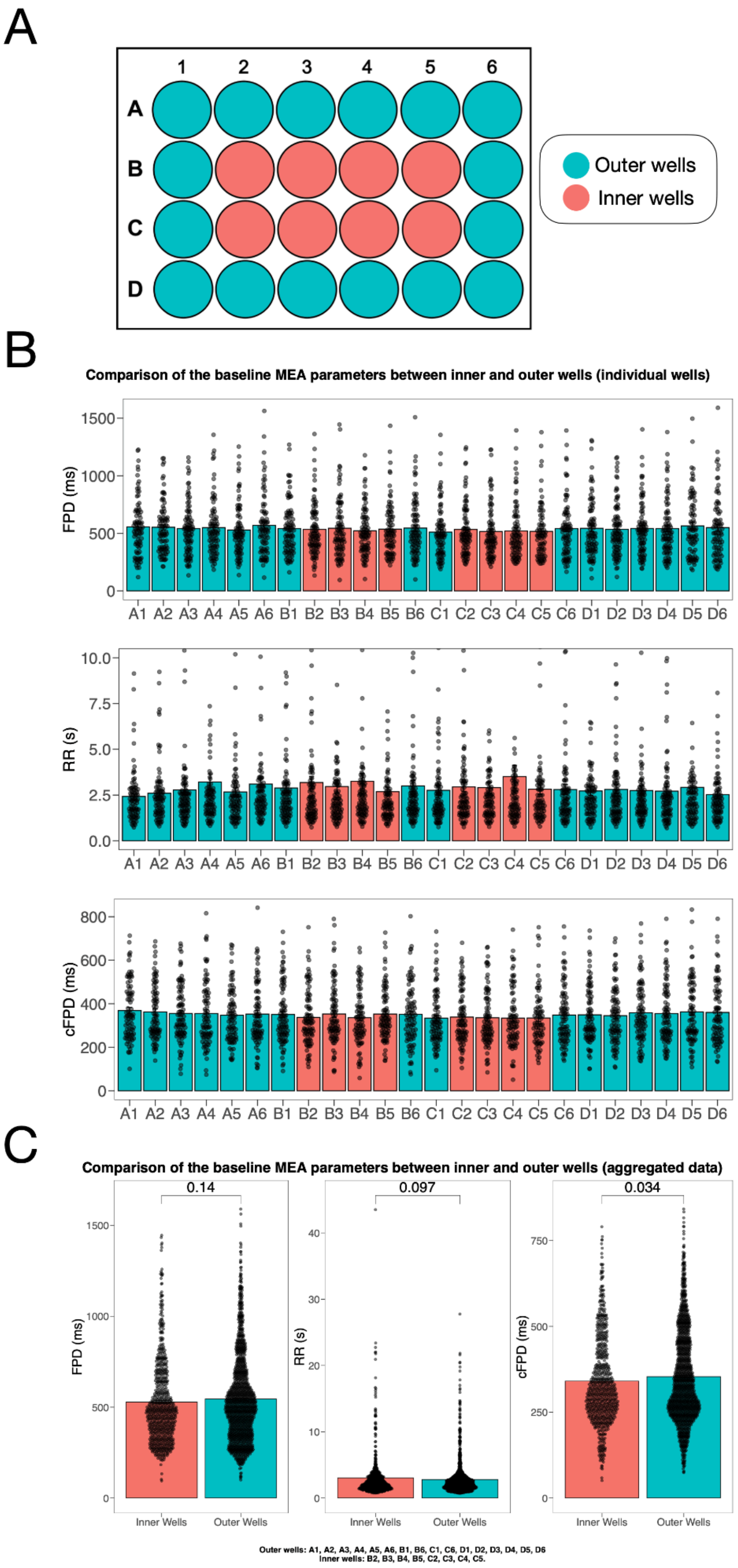
A
1
2
3
4
5
6
A
B
C
D
Outer wells
Inner wells
B
Comparison of the baseline MEA parameters between inner and outer wells (individual wells)
FPD (ms)
RR (s)
cFPD (ms)
A1 A2 A3 A4 A5 A6 B1 B2 B3 B4 B5 B6 C1 C2 C3 C4 C5 C6 D1 D2 D3 D4 D5 D6
C
Comparison of the baseline MEA parameters between inner and outer wells (aggregated data)
0.14
0.097
0.034
Inner Wells
Outer Wells
Outer wells: A1, A2, A3, A4, A5, A6, B1, B6, C1, C6, D1, D2, D3, D4, D5, D6
Inner wells: B2, B3, B4, B5, C2, C3, C4, C5.

Supplemental Figure 28. Electrophysiological readouts do not exhibit well-specific effects. A. Schematic map of a 24-well plate showing well coordinates. B. Field potential duration (FPD), RR interval, and corrected field potential duration (cFPD) measured across individual wells of the 24-well multiwell MEA. Corresponding well coordinates are indicated on the x-axis. C. Aggregated mean values for FPD, RR interval, and cFPD comparing inner wells (red) and outer wells (blue). Statistical analyses were performed using the t-test. N = 77-90 per well.

Supplemental references

1. Tohyama S, Hattori F, Sano M, Hishiki T, Nagahata Y, Matsuura T, Hashimoto H, Suzuki T, Yamashita H, Satoh Y, Egashira T, Seki T, Muraoka N, Yamakawa H, Ohgino Y, Tanaka T, Yoichi M, Yuasa S, Murata M, Suematsu M, Fukuda K. Distinct Metabolic Flow Enables Large-Scale Purification of Mouse and Human Pluripotent Stem Cell-Derived Cardiomyocytes. *Cell Stem Cell* 2013;**12**:127–137.

2. Buikema JW, Lee S, Goodyer WR, Maas RG, Chirikian O, Li G, Miao Y, Paige SL, Lee D, Wu H, Paik DT, Rhee S, Tian L, Galdos FX, Puluca N, Beyersdorf B, Hu J, Beck A, Venkamatran S, Swami S, Wijnker P, Schuldt M, Dorsch LM, Mil A van, Red-Horse K, Wu JY, Geisen C, Hesse M, Serpooshan V, Jovinge S, Fleischmann BK, Doevendans PA, Velden J van der, Garcia KC, Wu JC, Sluijter JPG, Wu SM. Wnt Activation and Reduced Cell-Cell Contact Synergistically Induce Massive Expansion of Functional Human iPSC-Derived Cardiomyocytes. *Cell Stem Cell* 2020;**27**:50-63.e5.

3. Feyen DAM, McKeithan WL, Bruyneel AAN, Spiering S, Hörmann L, Ulmer B, Zhang H, Briganti F, Schweizer M, Hegyi B, Liao Z, Pölönen R-P, Ginsburg KS, Lam CK, Serrano R, Wahlquist C, Kreymerman A, Vu M, Amatya PL, Behrens CS, Ranjbarvaziri S, Maas RGC, Greenhaw M, Bernstein D, Wu JC, Bers DM, Eschenhagen T, Metallo CM, Mercola M. Metabolic Maturation Media Improve Physiological Function of Human iPSC-Derived Cardiomyocytes. *Cell Rep* 2020;**32**:107925.

4. Sala L, Ward-van Oostwaard D, Tertoolen LGJ, Mummery CL, Bellin M. Electrophysiological Analysis of human Pluripotent Stem Cell-derived Cardiomyocytes (hPSC-CMs) Using Multi-electrode Arrays (MEAs). *J Vis Exp* 2017:55587.

5. Sala L, Leonov V, Mura M, Giannetti F, Khudiakov A, Moretti A, Crotti L, Gnecchi M, Schwartz PJ. Use of hiPSC-Derived Cardiomyocytes to Rule Out Proarrhythmic Effects of Drugs: The Case of Hydroxychloroquine in COVID-19. *Front Physiol* 2022;**12**:730127.

6. Mura M, Pisano F, Stefanello M, Ginevrino M, Boni M, Calabrò F, Crotti L, Valente EM, Schwartz PJ, Brink PA, Gnecchi M. Generation of two human induced pluripotent

stem cell (hiPSC) lines from a long QT syndrome South African founder population. *Stem Cell Res* 2019;**39**:101510.

7. Mura M, Pisano F, Stefanello M, Ginevrino M, Boni M, Calabrò F, Crotti L, Valente EM, Schwartz PJ, Brink PA, Gnecchi M. Generation of the human induced pluripotent stem cell (hiPSC) line PSMi007-A from a Long QT Syndrome type 1 patient carrier of two common variants in the NOS1AP gene. *Stem Cell Res* 2019;**36**:101416.

8. Mehta A, Ramachandra CJA, Singh P, Chitre A, Lua CH, Mura M, Crotti L, Wong P, Schwartz PJ, Gnecchi M, Shim W. Identification of a targeted and testable antiarrhythmic therapy for long-QT syndrome type 2 using a patient-specific cellular model. *Eur Heart J* 2018;**39**:1446–1455.

9. Pantazis CB, Yang A, Lara E, McDonough JA, Blauwendraat C, Peng L, Oguro H, Kanaujiya J, Zou J, Sebesta D, Pratt G, Cross E, Blockwick J, Buxton P, Kinner-Bibeau L, Medura C, Tompkins C, Hughes S, Santiana M, Faghri F, Nalls MA, Vitale D, Ballard S, Qi YA, Ramos DM, Anderson KM, Stadler J, Narayan P, Papademetriou J, Reilly L, Nelson MP, Aggarwal S, Rosen LU, Kirwan P, Pisupati V, Coon SL, Scholz SW, Priebe T, Öttl M, Dong J, Meijer M, Janssen LJM, Lourenco VS, Van Der Kant R, Crusius D, Paquet D, Raulin A-C, Bu G, Held A, Wainger BJ, Gabriele RMC, Casey JM, Wray S, Abu-Bonsrah D, Parish CL, Beccari MS, Cleveland DW, Li E, Rose IVL, Kampmann M, Calatayud Aristoy C, Verstreken P, Heinrich L, Chen MY, Schüle B, Dou D, Holzbaur ELF, Zanellati MC, Basundra R, Deshmukh M, Cohen S, Khanna R, Raman M, Nevin ZS, Matia M, Van Lent J, Timmerman V, Conklin BR, Johnson Chase K, Zhang K, Funes S, Bosco DA, Erlebach L, Welzer M, Kronenberg-Versteeg D, Lyu G, Arenas E, Coccia E, Sarrafha L, Ahfeldt T, Marioni JC, Skarnes WC, Cookson MR, Ward ME, Merkle FT. A reference human induced pluripotent stem cell line for large-scale collaborative studies. *Cell Stem Cell* 2022;**29**:1685-1702.e22.

10. Mura M, Lee Y-K, Pisano F, Ginevrino M, Boni M, Calabrò F, Crotti L, Valente EM, Schwartz PJ, Tse H-F, Gnecchi M. Generation of the human induced pluripotent stem cell (hiPSC) line PSMi005-A from a patient carrying the KCNQ1-R190W mutation. *Stem Cell Res* 2019;**37**:101437.

11. Mura M, Lee Y-K, Pisano F, Ginevrino M, Boni M, Calabrò F, Crotti L, Valente EM, Schwartz PJ, Tse H-F, Gnecchi M. Generation of the human induced pluripotent stem cell (hiPSC) line PSMi004-A from a carrier of the KCNQ1-R594Q mutation. *Stem Cell Res* 2019;**37**:101431.

12. Ronchi C, Bernardi J, Mura M, Stefanello M, Badone B, Rocchetti M, Crotti L, Brink P, Schwartz PJ, Gnecchi M, Zaza A. NOS1AP polymorphisms reduce NOS1 activity and interact with prolonged repolarization in arrhythmogenesis. *Cardiovasc Res* 2021;**117**:472–483.

13. Mura M, Lee Y, Ginevrino M, Zappatore R, Pisano F, Boni M, Dagradi F, Crotti L, Valente EM, Schwartz PJ, Tse H-F, Gnecchi M. Generation of the human induced pluripotent stem cell (hiPSC) line PSMi002-A from a patient affected by the Jervell and Lange-Nielsen syndrome and carrier of two compound heterozygous mutations on the KCNQ1 gene. *Stem Cell Res* 2018;**29**:157–161.

14. Mehta A, Sequiera GL, Ramachandra CJA, Sudibyo Y, Chung Y, Sheng J, Wong KY,

Tan TH, Wong P, Liew R, Shim W. Re-trafficking of hERG reverses long QT syndrome 2 phenotype in human iPS-derived cardiomyocytes. *Cardiovasc Res* 2014;**102**:497–506.

15. Thomas D, Wu K, Kathöfer S, Katus HA, Schoels W, Kiehn J, Karle CA. The antipsychotic drug chlorpromazine inhibits HERG potassium channels. *Br J Pharmacol* 2003;**139**:567–574.

16. Blinova K, Dang Q, Millard D, Smith G, Pierson J, Guo L, Brock M, Lu HR, Kraushaar U, Zeng H, Shi H, Zhang X, Sawada K, Osada T, Kanda Y, Sekino Y, Pang L, Feaster TK, Kettenhofen R, Stockbridge N, Strauss DG, Gintant G. International Multisite Study of Human-Induced Pluripotent Stem Cell-Derived Cardiomyocytes for Drug Proarrhythmic Potential Assessment. *Cell Rep* 2018;**24**:3582–3592.

17. Kang J, Wang L, Chen X-L, Triggle DJ, Rampe D. Interactions of a Series of Fluoroquinolone Antibacterial Drugs with the Human Cardiac K^+ Channel HERG. *Mol Pharmacol* 2001;**59**:122–126.

18. Owens RC, Patel KB, Banevicius MA, Quintiliani R, Nightingale CH, Nicolau DP. Oral bioavailability and pharmacokinetics of ciprofloxacin in patients with AIDS. *Antimicrob Agents Chemother* 1997;**41**:1508–1511.

19. Stanat SJC, Carlton CG, Crumb Jr WJ, Agrawal KC, Clarkson CW. Characterization of the inhibitory effects of erythromycin and clarithromycin on the HERG potassium channel. *Mol Cell Biochem* 2003;**254**:1–7.

20. Orvos P, Kohajda Z, Szlovák J, Gazdag P, Árpádffy-Lovas T, Tóth D, Geramipour A, Tálosi L, Jost N, Varró A, Virág L. Evaluation of Possible Proarrhythmic Potency: Comparison of the Effect of Dofetilide, Cisapride, Sotalol, Terfenadine, and Verapamil on hERG and Native *I* Kr Currents and on Cardiac Action Potential. *Toxicol Sci* 2019;**168**:365–380.

21. Zhou Z, Gong Q, Ye B, Fan Z, Makielski JC, Robertson GA, January CT. Properties of HERG Channels Stably Expressed in HEK 293 Cells Studied at Physiological Temperature. *Biophys J* 1998;**74**:230–241.

22. Suessbrich H, Schönherr R, Heinemann SH, Attali B, Lang F, Busch AE. The inhibitory effect of the antipsychotic drug haloperidol on HERG potassium channels expressed in *Xenopus* oocytes. *Br J Pharmacol* 1997;**120**:968–974.

23. Uematsu T, Matsuno H, Sato H, Hirayama H, Hasegawa K, Nakashima M. Steady-State Pharmacokinetics of Haloperidol and Reduced Haloperidol in Schizophrenic Patients: Analysis of Factors Determining their Concentrations in Hair. *J Pharm Sci* 1992;**81**:1008–1011.

24. Bischoff U, Schmidt C, Netzer R, Pongs O. Effects of fluoroquinolones on HERG currents. *Eur J Pharmacol* 2000;**406**:341–343.

25. Stass H, Dalhoff A, Kubitza D, Schühly U. Pharmacokinetics, Safety, and Tolerability of Ascending Single Doses of Moxifloxacin, a New 8-Methoxy Quinolone, Administered to Healthy Subjects. *Antimicrob Agents Chemother* 1998;**42**:2060–2065.

26. Kruizinga MD, Birkhoff WAJ, Van Esdonk MJ, Klarenbeek NB, Cholewinski T,

Nelemans T, Dröge MJ, Cohen AF, Zuiker RGJA. Pharmacokinetics of intravenous and inhaled salbutamol and tobramycin: An exploratory study to investigate the potential of exhaled breath condensate as a matrix for pharmacokinetic analysis. *Br J Clin Pharmacol* 2020;**86**:175–181.

27. Westenskow P, Splawski I, Timothy KW, Keating MT, Sanguinetti MC. Compound Mutations: A Common Cause of Severe Long-QT Syndrome. *Circulation* 2004;**109**:1834–1841.

28. Brink PA, Crotti L, Corfield V, Goosen A, Durrheim G, Hedley P, Heradien M, Geldenhuys G, Vanoli E, Bacchini S, Spazzolini C, Lundquist AL, Roden DM, George AL, Schwartz PJ. Phenotypic Variability and Unusual Clinical Severity of Congenital Long-QT Syndrome in a Founder Population. *Circulation* 2005;**112**:2602–2610.

29. Heijman J, Spätjens RLHMG, Seyen SRM, Lentink V, Kuijpers HJH, Boulet IR, De Windt LJ, David M, Volders PGA. Dominant-Negative Control of cAMP-Dependent I_{Ks} Upregulation in Human Long-QT Syndrome Type 1. *Circ Res* 2012;**110**:211–219.

30. Wang Z, Tristani‐Firouzi M, Xu Q, Lin M, Keating MT, Sanguinetti MC. Functional Effects of Mutations in KvLQT1 that Cause Long QT Syndrome. *J Cardiovasc Electrophysiol* 1999;**10**:817–826.

31. Ficker E, Dennis AT, Obejero-Paz CA, Castaldo P, Taglialatela M, Brown AM. Retention in the Endoplasmic Reticulum as a Mechanism of Dominant-negative Current Suppression in Human Long QT Syndrome. *J Mol Cell Cardiol* 2000;**32**:2327–2337.

32. Anderson CL, Delisle BP, Anson BD, Kilby JA, Will ML, Tester DJ, Gong Q, Zhou Z, Ackerman MJ, January CT. Most LQT2 Mutations Reduce Kv11.1 (hERG) Current by a Class 2 (Trafficking-Deficient) Mechanism. *Circulation* 2006;**113**:365–373.

33. Garg P, Oikonomopoulos A, Chen H, Li Y, Lam CK, Sallam K, Perez M, Lux RL, Sanguinetti MC, Wu JC. Genome Editing of Induced Pluripotent Stem Cells to Decipher Cardiac Channelopathy Variant. *J Am Coll Cardiol* 2018;**72**:62–75.

34. Song L, Bekdash R, Morikawa K, Quejada JR, Klein AD, Aina-Badejo D, Yoshida K, Yamamoto HE, Chalan A, Yang R, Patel A, Sirabella D, Lee TM, Joseph LC, Kawano F, Warren JS, Soni RK, Morrow JP, Yazawa M. Sigma non-opioid receptor 1 is a potential therapeutic target for long QT syndrome. *Nat Cardiovasc Res* 2022;**1**:142–156.

35. Garg P, Oikonomopoulos A, Chen H, Li Y, Lam CK, Sallam K, Perez M, Lux RL, Sanguinetti MC, Wu JC. Genome Editing of Induced Pluripotent Stem Cells to Decipher Cardiac Channelopathy Variant. *J Am Coll Cardiol* 2018;**72**:62–75.

36. Cui J, Kagan A, Qin D, Mathew J, Melman YF, McDonald TV. Analysis of the Cyclic Nucleotide Binding Domain of the HERG Potassium Channel and Interactions with KCNE2. *J Biol Chem* 2001;**276**:17244–17251.

37. Ficker E, Zhao S, Obejero-Paz CA, Brown AM. The Binding Site for Channel Blockers That Rescue Misprocessed Human Long QT Syndrome Type 2 ether-a-gogo-related Gene (HERG) Mutations. *J Biol Chem* 2002;**277**:4989–4998.

38. Akhavan A, Atanasiu R, Noguchi T, Han W, Holder N, Shrier A. Identification of the

cyclic-nucleotide-binding domain as a conserved determinant of ion-channel cell-surface localization. *J Cell Sci* 2005;**118**:2803–2812.
